# Assessment of the phi6 lysis system using genetic complementation and heterologous expression in *Escherichia coli* and *Pseudomonas syringae*

**DOI:** 10.3389/fmicb.2025.1718418

**Published:** 2026-01-07

**Authors:** Devyn Del Curto, Micaela Zamora, Greyson Lasley, Rabia Khan, Katlyn Montalbano, J. Bryce Ricken, Jesse Cahill

**Affiliations:** Environmental Systems Biology, Sandia National Labs, Albuquerque, NM, United States

**Keywords:** cystoviridae, outer membrane disruptor, disruptin, spanin, holin, P10, P5

## Abstract

The RNA cystovirus phi6 represents a unique evolutionary outlier, encoding a holin and endolysin similar to tailed phages, yet lacking an identified gene for outer membrane disruption. In this study, we investigated the phi6 lysis system using genetic complementation experiments and heterologous expression in *Escherichia coli* and *Pseudomonas syringae*. Our results demonstrate that the phi6 endolysin is functional in both bacterial systems, while the phi6 holin does not show activity in either bacterial system when expressed using moderate expression systems. Combinations of lambda lysis gene controls performed as expected in *P. syringae*. The phi6 holin requires overexpression or co-expression with other phi6 genes to exhibit lysis in *E. coli*. Overexpression of plasmids containing entire phi6 S-, M-, and L-segments cDNA in *E. coli* produced lysis profiles and cell morphology consistent with holin-endolysin expression, but outer membrane disruption was not observed. This suggests either phi6 has not evolved to carry an outer membrane disruptor or that the outer membrane disruptor is not active in our *E. coli* testbed. Our findings highlight the unusual nature of the phi6 lysis system.

## 1 Introduction

**Figure 1:**
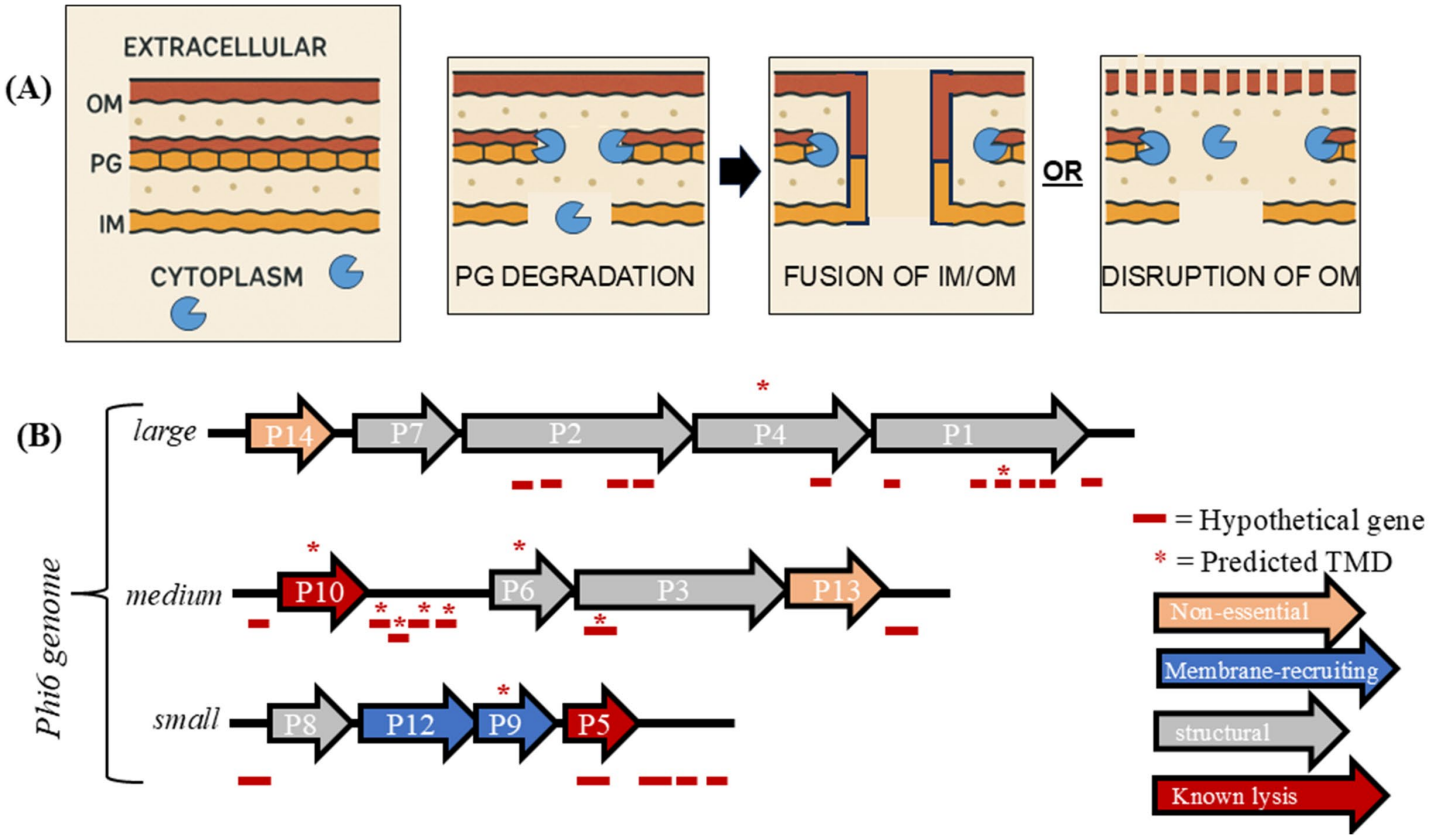
**(A)** Cartoon model of phage lysis of a Gram-negative cellular envelope by multicomponent lysis system. **(B)** The 3 segments of the phi6 genome are shown along with gene product roles identified [see reviews by Gottlieb and Alimova (2023) and Callanan et al. (2018)]. Hypothetical genes are shown as red dashes and TMD-containing genes are shown with asterisks. The identification of hypothetical and TMDs are described in the methods section.

Bacteriophage lysis is an essential process in the phage lifecycle, enabling the release of progeny phages from infected host cells. While filamentous phages extrude progeny through the host cell envelope without lysing the host (Cahill and Young, 2021), most phages carry genes coding for membrane and cell wall disruption systems to destroy the host cell envelope (Cahill and Young, 2019). Tailed phages, such as lambda, employ multicomponent lysis systems that target each layer of the bacterial cell envelope. These systems typically include a holin to permeabilize the inner membrane (IM), an endolysin to degrade the peptidoglycan (PG) layer, and a third component—spanins (Berry et al., 2012) or disruptins (Holt et al., 2022)—to disrupt the outer membrane (OM) in Gram-negative bacteria (Cahill and Young, 2019) (Figure 1A).

Spanins are fusogenic proteins that remove the OM barrier by fusing it to the IM after PG degradation (Rajaure et al., 2015). Spanin candidates are relatively easy to identify in phage genomes due to their signal sequence and transmembrane domain (TMD). However, a significant percentage of *Caudovirales* phages of Gram-negative bacteria (13%) lack identifiable spanins (Kongari et al., 2018), suggesting alternative mechanisms for outer membrane disruption. This led to the discovery of disruptins, such as the archetype disruptin of PhiKT, which is a short amphipathic helix peptide with antimicrobial effects similar to the model antimicrobial peptide LL-37 (Holt et al., 2022). Disruptins are phage-encoded antimicrobial peptides, but their diversity complicates the systematic prediction of disruptin genes in phage genomes. Whether phages lacking spanins encode disruptins or other types of outer membrane disruptors remains an open question.

Most RNA phages, in contrast, often achieve lysis by expressing a single lysis gene that inhibits PG synthesis (Cahill and Young, 2021). DNA phages, however, rely on multicomponent lysis systems, which allow precise regulation of lysis timing via the holin “lysis clock.” This regulation optimizes the number of phages released per cell, allowing the phage to evolve lysis timing specific to environmental conditions and maximizing fitness (Cahill and Young, 2019).

Cystovirus phi6 of *Pseudomonas syringae* is phage with a double stranded RNA genome comprised of three segments (S, M, L) (Gottlieb and Alimova, 2022). Phi6 carries a multicomponent lysis system, representing a fascinating example of convergent evolution. Phi6 encodes genes for a holin (P10) and endolysin (P5), resembling the lysis systems of tailed DNA phages (Young et al., 2005) (Figure 1B). Despite the similarity, the gene responsible for outer membrane disruption in phi6 has not been identified, leaving a critical gap in our understanding of its lysis mechanism.

Here, we sought to identify the outer membrane disruptor of phi6 and further characterize its known lysis genes. While other cystoviruses are known (Mäntynen et al., 2018; Mäntynen et al., 2023), phi6 is the most extensively studied, and its lysis system has been partially investigated. Previous studies have identified P5 and P10 as the phi6 endolysin and holin, respectively, through genomic mutational analyses and *in vitro* enzymatic assays (Johnson et al., 1994; Mindich and Lehman, 1979). However, *in vivo* characterization methods, such as lysis curves with internal controls comparing phi6 genes to well-characterized systems like lambda, have not been reported. To address this, we performed genetic complementation experiments using the lambda holin-endolysin system and systematically tested pairings of phi6 and lambda lysis components in *Escherichia coli* and *P. syringae*. These experiments aimed to evaluate the functionality of phi6 lysis proteins and investigate the lysis system of this unusual RNA phage, which encodes a multicomponent lysis system more commonly associated with tailed DNA phages.

## 2 Materials and methods

### 2.1 Bacterial strains and growth conditions

*Escherichia coli* NEBα (New England Biolabs), *E. coli* Lemo21 (DE3, 13), *P. syringae* (Humphrey et al., 2023) were used in this study. Cultures were maintained in LB medium supplemented with 50 μg/mL spectinomycin (LB-spec) for constructs carrying pJLC140 or its derivatives. L-Rhamnose and 10 μg/mL chloramphenicol were provided at 0.2% to quench expression of genes under control of the T7 promoter in Lemo21 (DE3) cultures. Kanamycin was supplemented at 50 μg/mL to cultures carrying pJLC141. Single colonies were used to inoculate 4 mL or 2 mL of LB supplemented with appropriate antibiotics, and cultures were incubated with aeration at 37 °C for 18 h (*E. coli* NEBα) or at 28 °C for 24 h (*P. syringae*) prior to subculturing in preparation of lysis assays.

### 2.2 Phage genes, shuttle vector, and cloning

The supplementary files (under DNA sequences header) provide the sequences used for all DNA constructs in this study. We used TMHMM (Reddy et al., 2014) to identify genes within phi6 that contain predicted TMDs. Hypothetical genes in phi6 were identified by visual inspection by examining open reading frames that contained a ribosome binding site (Vimberg et al., 2007) 5–13 nucleotides upstream of a translational start codon that coded for a product larger than 25 amino acids (aa). The pJLC140 shuttle vector was constructed using the Golden Gate assembly kit (BsaI, NEB #E1601L) following the manufacturer’s recommended protocols. Assembly involved combining three synthetic DNA gBlocks with an amplicon generated from the pJH1 vector (Ho et al., 2020) (Addgene #154204) using primers 653 and 652. The pJLC140 vector includes a p15A origin of replication (low copy number), a pro1600 replicon (Lu et al., 2002) for compatibility with *Pseudomonas*, and a lambda lysis gene cassette under the control of the P-araBAD promoter. BsmBI restriction sites flank the lambda lysis cassette in the correct orientation, enabling the insertion of gBlocks containing various combinations of phi6 and lambda lysis genes. Synthetic dsDNA gBlocks were produced by Integrated DNA Technologies (IDT), with flanking BsmBI sites to support Golden Gate assembly (NEB #E1602). Phi6 genomic cDNA segments were produced using IDT gblocks and used with NEB’s BsaI-HF v2 kit (NEB #E1602) to produce plasmids pJLC141, pJLC142, and pJLC143, which carry phi6 S, M, and L segment, respectively. To facilitate Golden Gate cloning, we introduced 3,7, and 6 single letter mutations into the S, M, and L segment gblocks. These mutations were silent with respect to the amino acid sequence of phi6, using accession numbers M12921, M17462, M17461 as reference sequences for S, M, and L, respectively.

All three segments were cloned into the pYES1yeast YAC/BAC shuttle vector using GeneArt High-Order Genetic Assembly System (Thermo Fisher Scientific, #A13286) according to manufacturer’s protocols. Primers 642–649 (Supplementary Table 1) were used with pJLC141-143 plasmids to generate phi6 cDNA amplicons with flanking T7 promoter and T7 terminator using Platinum SuperFi II DNA Polymerase (Thermo Fisher Scientific, #12361010) according to manufacturer’s protocols. Phi6 cDNA constructs were verified by whole-plasmid sequencing using Plasmidsaurus nanopore sequencing.

Golden Gate assemblies were transformed into NEBα chemically competent cells following the manufacturer’s protocols and plated on LB-spec agar plates. Colony PCR was performed using NEB Taq Polymerase (M0273L) with primers 181 and 658 (Supplementary Table 1) to confirm successful insertion. Amplicons were sequenced by Plasmidsaurus, and colonies carrying verified constructs were inoculated in LB-spec as described above and stored at −80 °C with 25% glycerol.

Sequence-verified derivatives of pJLC140 carrying lysis gene combinations were purified using the Qiagen miniprep kit according to the manufacturer’s protocols. *P. syringae* cultures were transformed by electroporation using established methods (Choi et al., 2006), except 20% sucrose was used instead of glycerol, and transformants were selected on LB-spec agar plates after incubation at 28 °C for 2 days.

### 2.3 *In vivo* expression of lysis genes in *Pseudomonas syringae and Escherichia coli* for testing lysis phenotype

Prior to plate reader monitoring for lysis, cultures were subcultured 1: 100 (v/v) for 2 h into culture tubes containing LB media supplemented with appropriate antibiotics and 10 mM Mg++ (unless indicated otherwise) and incubated at their respective temperatures. Cation supplementation strengthens the outer membrane of Gram-negative bacteria, allowing differentiation between cultures expressing a complete lysis cassette and those lacking an outer membrane disruptor under aerated conditions (Berry et al., 2012). Cultures were induced with L-arabinose at a final concentration of 0.4% (or left uninduced for the pJLC140 control), and 200 μL of culture was transferred to wells of CellVis plates (Cellvis, formerly *In Vitro* Scientific) in triplicate. To minimize edge effects, 200 μL of sterile water was added to all outer wells of the plate. Lysis was monitored by measuring absorbance at 600 nm every 10 min at the cultures’ respective temperatures using a BioTek Cytation 5 plate reader (Agilent, CA United States). The plate reader maintained continuous shaking between timepoints, and lysis assays were conducted for approximately 24 h.

### 2.4 Microscopy

Imaging of *E. coli* or *P. syringae* cells expressing lysis gene combinations was performed using an EVOS M5000 microscope (Invitrogen) equipped with a 40 × /0.65 numerical aperture objective. 2–3 μL culture were placed on a glass slide and covered with a coverslip. Parallel cultures were run alongside plate reader experiments under identical settings, except samples were collected 4.5 h and ~8 h after induction for *E. coli* and *P. syringae*, respectively.

## 3 Results and discussion

**Figure 2:**
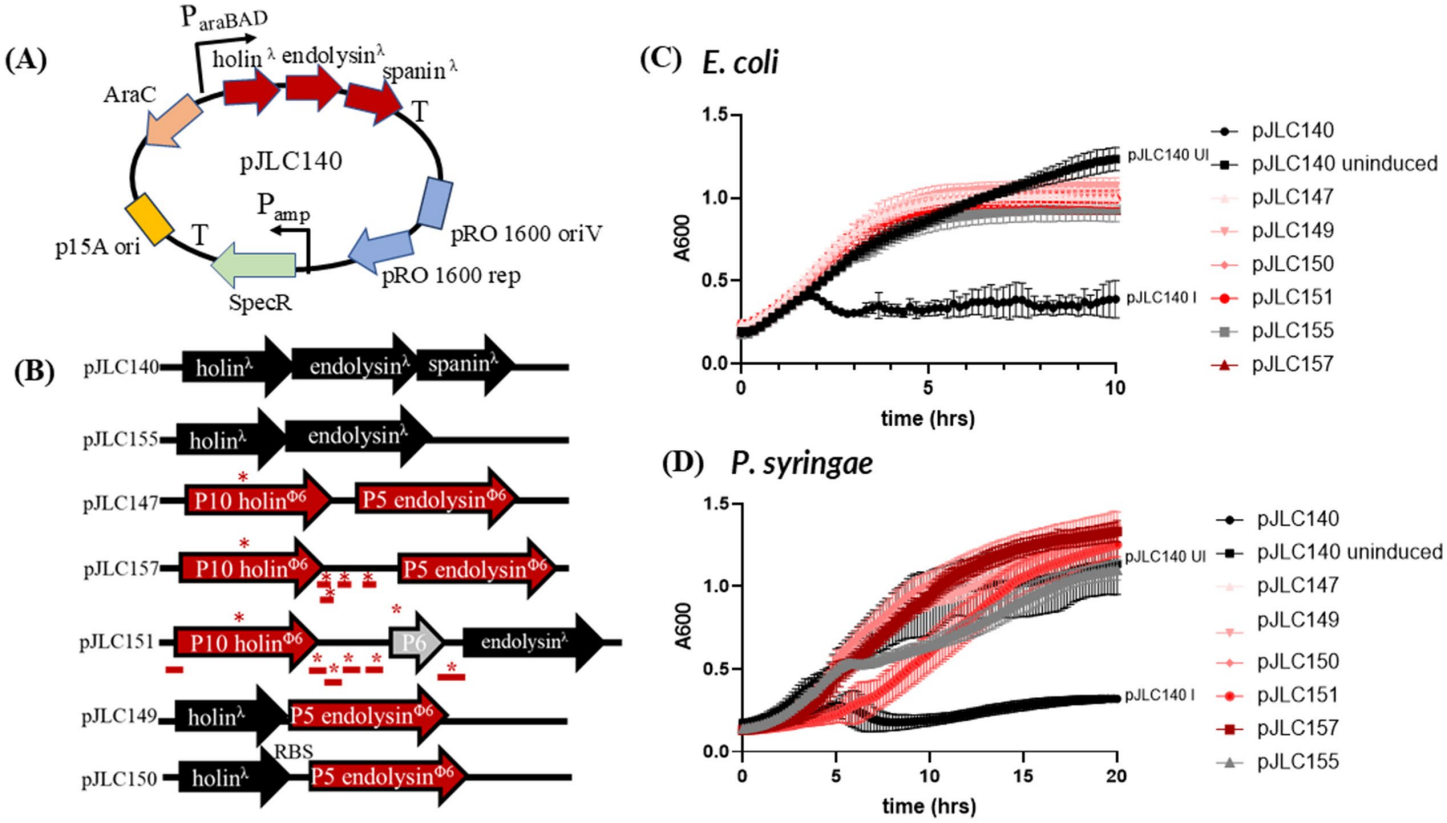
Testing holin/endolysin and control combinations. **(A)** Shuttle vector used to test lysis gene combinations. T = terminator. **(B)** Diagram of different combinations of Phi6 or Lambda holin and endolysin combinations used in panel C and D. Constructs replace the Lambda holin-endolysin-spanin shown in panel A. **(C)** Lysis profile of constructs tested in *E. coli.* **(D)** Lysis profile of constructs tested in *P. syringae.* All constructs were induced at time zero (except the uninduced control) and absorbance at 600 nm (*A*_600_) was monitored every 10 min overnight with shaking. The average of two biological replicates is shown and the error bars represent standard deviation. The graphs were truncated mid-run, as lysis occurred ~3 h after induction for *E. coli* and ~6 h after induction for *P. syringae.* *TMD-containing genes.

We first sought to characterize the previously reported lysis genes of *P. syringae* using isogenic controls derived from the lambda lysis system. The pJLC140 shuttle vector (Figure 2A) contains a low-copy p15A replicon for *E. coli* and a Pro1600 replicon (Lu et al., 2002) for *Pseudomonas*. This vector utilizes an arabinose induction system, which has been shown to function effectively in *Pseudomonas* (McMackin et al., 2021). As a positive control, pJLC140 encodes the complete lambda lysis cassette under arabinose control, with induced and uninduced conditions serving as controls in our system. A second lambda control, pJLC155, expresses only the lambda holin and endolysin, lacking an outer membrane disruptor. In this case, lysis is blocked, and cells transition from a rod to a spherical shape as the PG is degraded, but the cell contents remain bound by the outer membrane (Cahill and Young, 2019; Berry et al., 2012). These cells are physiologically dead due to holin-mediated permeabilization of the IM, as shown previously (Cahill and Young, 2019). Figure 2 demonstrates that we can distinguish three classes of controls for both *P. syringae* and *E. coli:* (1) a fully induced lysis system, (2) uninduced lysis showing normal growth, and (3) the pJLC155 control, which produces an intermediate result in the lysis curve. For both *P. syringae* and *E. coli*, A600 readings after lysis for pJLC155 were lower than the uninduced pJLC140 but easily distinguished from the induced pJLC140, as expected and shown previously for lambda inductions in *E. coli* (Berry et al., 2012; Cahill et al., 2017). We investigated the morphology of cells following induction using phase contrast microscopy, which revealed expected trends for our controls in both cell lines. Induced pJLC140 cultures predominantly displayed rod-shaped “ghost cells,” indicative of complete lysis (Cahill et al., 2024) (Supplementary Figure S1). In contrast, uninduced pJLC140 cultures exhibited live, intact rod-shaped cells, reflecting normal growth. The holin-endolysin control, pJLC155, showed the anticipated spherical cell morphology, consistent with PG degradation (Cahill et al., 2017; Cahill et al., 2024) while the outer membrane remained intact. Taken together, our lysis testbed demonstrates that we can differentiate intact lysis systems from those lacking an outer membrane disruptor.

### 3.1 Holin-endolysin combinations from phi6 and lambda do not produce typical lysis profiles

Building on the characterization of lambda lysis controls, we systematically tested different combinations of phi6 lysis genes alongside lambda lysis genes to evaluate their functionality in *E. coli* and *P. syringae*.

In *E. coli*, constructs pJLC147, pJLC157, pJLC151, pJLC149, and pJLC150 produced lysis curves indistinguishable from the lambda holin-endolysin control (pJLC155), indicating that these constructs encode a defective lysis system (Figure 2C). pJLC147 contains phi6 holin and phi6 endolysin, whereas pJLC157 includes four hypothetical genes downstream of the phi6 holin, which encode products with predicted TMDs, which are described in more detail in the text below. pJLC151 expands the hypothetical gene cluster to include one non-TMD-containing hypothetical upstream of the holin, along with the gene for P6 and the 5′ end of the gene for P3, which carries an embedded hypothetical gene encoding a predicted TMD.

Microscopy revealed that pJLC147, pJLC151, and pJLC157 cultures predominantly displayed intact rod-shaped cells (Supplementary Figure S2), suggesting that phi6 holin, phi6 holin-endolysin, and the genes near the phi6 holin are not sufficient to disrupt the IM or PG Even when the largest gene cluster surrounding the phi6 holin was paired with the lambda endolysin (pJLC151), no morphological evidence of disruption of the IM or PG was observed. Conversely, pJLC149 and pJLC150, which pair the lambda holin with phi6 endolysin (pJLC150 contains an optimized ribosome binding site) produced spherical cells, similar to pJLC155, indicative of PG degradation with the outer membrane remaining intact. These observations indicate that the phi6 endolysin is active in *E. coli* when paired with lambda holin; however, the phi6 holin does not function in *E. coli*.

In *P. syringae*, the lysis curves for pJLC147, pJLC157, pJLC149, and pJLC150 resembled pJLC140 uninduced, showing that these constructs are defective for lysis (Figure 2D). However, pJLC151 showed delayed growth, which might be due to toxicity of expressing numerous TMD-containing proteins (Schlegel et al., 2012). Microscopy revealed that pJLC147 cultures displayed intact rod-shaped cells (Supplementary Figure S3), similar to the uninduced control (pJLC140 uninduced). Lambda-holin/ phi6-endolysin constructs pJLC49 and pJLC150 showed rod-shaped cells with a small population of spherical cells. This was also observed in phi6 holin (lambda or phi6) endolysin pairings that contained the additional genes downstream of the phi6 holin. This suggests that these additional TMD-containing genes that are located near the holin have a role in holin function, similar to what is observed in Gram-positive bacteria that require simultaneous action of more than one holin gene (Fernandes and São-José, 2017; Fernandes and São-José, 2016). Notably, we observe that rod-shaped cells dominate number of spherical cells in *P. syringae*, indicating that the activity of phi6 holin-endolysin components in our testbed is low in the native host.

Although constructs combining phi6 holin and endolysin alone or with hypothetical genes failed to produce the expected lysis profiles in either species, we next sought to identify potential outer membrane disruptors encoded by phi6 that could complement the lambda holin-endolysin system.

### 3.2 Searching the S segment for outer membrane disruptors

The S segment of the phi6 genome contains five hypothetical genes. The first is located in the +1 reading frame of the P8 gene and encodes a 36 aa peptide. The second hypothetical gene is in the +2 reading frame of the P5 (endolysin) gene and encodes a 66 aa product. Three additional hypothetical genes are located downstream of the P5 gene, encoding products of 111, 42, and 39 aas, respectively. None of these hypothetical genes contain predicted TMDs.

**Figure 3:**
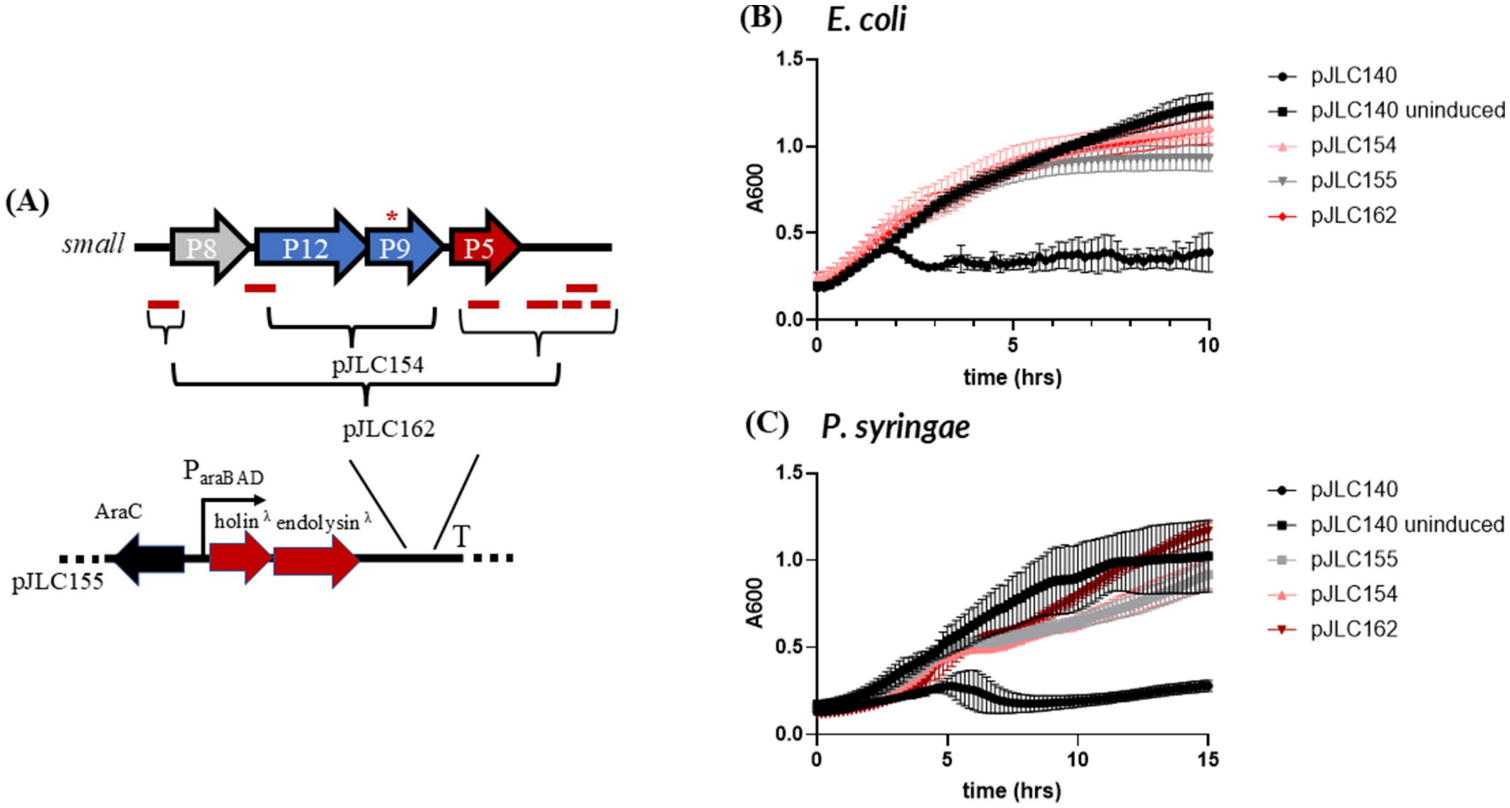
Testing outer membrane disruptor candidates from the small segment of the phi6 genome. **(A)** Shows the different combinations of candidate outer membrane disruptor genes that were cloned downstream of the lambda holin-endolysin vector, pJLC155. **(B)** Lysis profile of constructs tested in *E. coli.* **(C)** Lysis profile of constructs tested in *P. syringae.* All constructs were induced at time zero (except the uninduced control) and absorbance at 600 nm (*A*_600_) was monitored every 10 min overnight with shaking. The average of two biological replicates is shown and the error bars represent standard deviation. *TMD-containing genes.

To test whether any of these hypothetical genes function as outer membrane disruptors, we constructed pJLC162, which carries the S-segment hypothetical genes described above placed downstream of the lambda holin-endolysin cassette (Figure 3A). We also tested pJLC154, which carries P12 and P9 downstream of the lambda holin-endolysin cassette. Among the phi6 S-segment genes, P9 is the only one that encodes a predicted TMD, while P9/P12 expression is linked to membrane recruitment during phi6 assembly (Johnson 3rd and Mindich, 1994). Overexpression of P12 and P9 has been reported to cause vesicle formation (Lyytinen et al., 2019), making them strong candidates for outer membrane disruptors.

Neither pJLC162 nor pJLC154 showed a phenotype consistent with an intact lysis cassette in either strain, like pJLC140 (Figures 3B,C). Instead, both constructs resembled pJLC155 in cell morphology and lysis curves for both strains (Supplementary Figure S4), indicating that none of the hypothetical genes tested in pJLC162 or pJLC154, including P9 and P12, are involved in outer membrane disruption.

### 3.3 Searching the M segment for outer membrane disruptors

The M segment of the phi6 genome contains seven hypothetical genes. The first is located upstream of the holin (P10) and encodes a 41-aa acid peptide without a predicted TMD. Four additional genes encode peptides with predicted TMDs, with lengths of 73, 53, 33, and 48 aa. Given their proximity to the holin in the genome and the presence of predicted TMDs, these genes may function as multi-gene holins (discussed above), antiholins (Cahill and Young, 2019) or are candidates for outer membrane disruptor genes.

**Figure 4:**
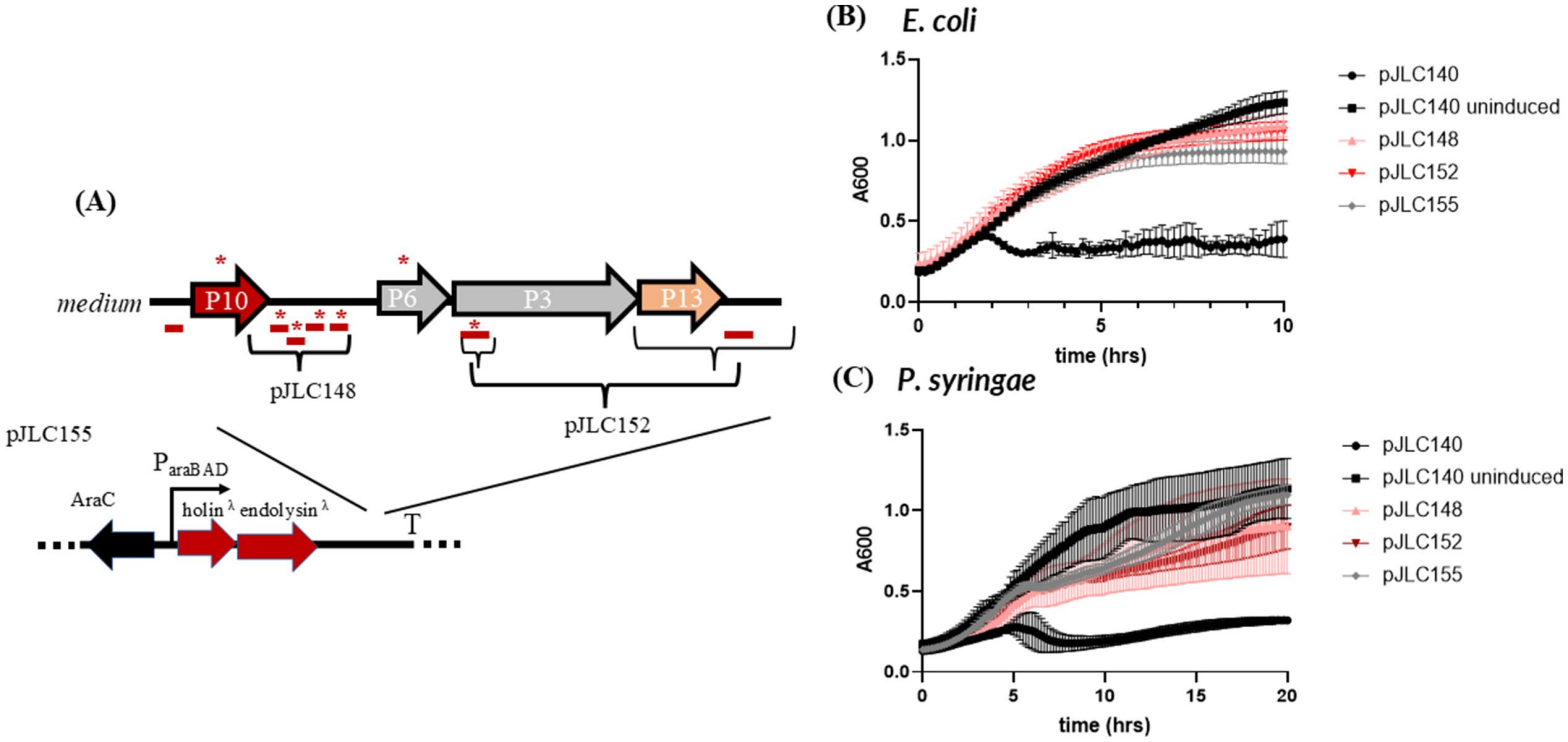
Testing outer membrane disruptor candidates from the medium segment of the phi6 genome. **(A)** Shows the different combinations of candidate outer membrane disruptor genes that were cloned downstream of the lambda holin-endolysin vector, pJLC155. **(B)** Lysis profile of constructs tested in *E. coli.* **(C)** Lysis profile of constructs tested in *P. syringae.* All constructs were induced at time zero (except the uninduced control) and absorbance at 600 nm (*A*_600_) was monitored every 10 min overnight with shaking. The average of two biological replicates is shown and the error bars represent standard deviation. *TMD-containing genes.

Since spanins contain TMDs and are known to mediate outer membrane disruption, it seemed reasonable to hypothesize that the four TMD-containing genes downstream of the holin could encode outer membrane disruptors. To test this, we cloned these genes downstream of the lambda holin-endolysin cassette in pJLC148. Additionally, pJLC152 was constructed to include the 5′ end of the P3 (spike) gene, which contains an embedded gene in the +1 reading frame encoding a predicted 110 aa TMD-containing protein. pJLC152 also includes an 87 aa gene downstream of P13 that does not encode a predicted TMD (Figure 4A).

Neither pJLC148 nor pJLC152 showed a lysis profile or morphology (Supplementary Figure S5, Figures 4B,C) distinct from pJLC155 in either species, indicating that these are not outer membrane disruptor genes.

### 3.4 Searching the L segment for outer membrane disruptors

**Figure 5:**
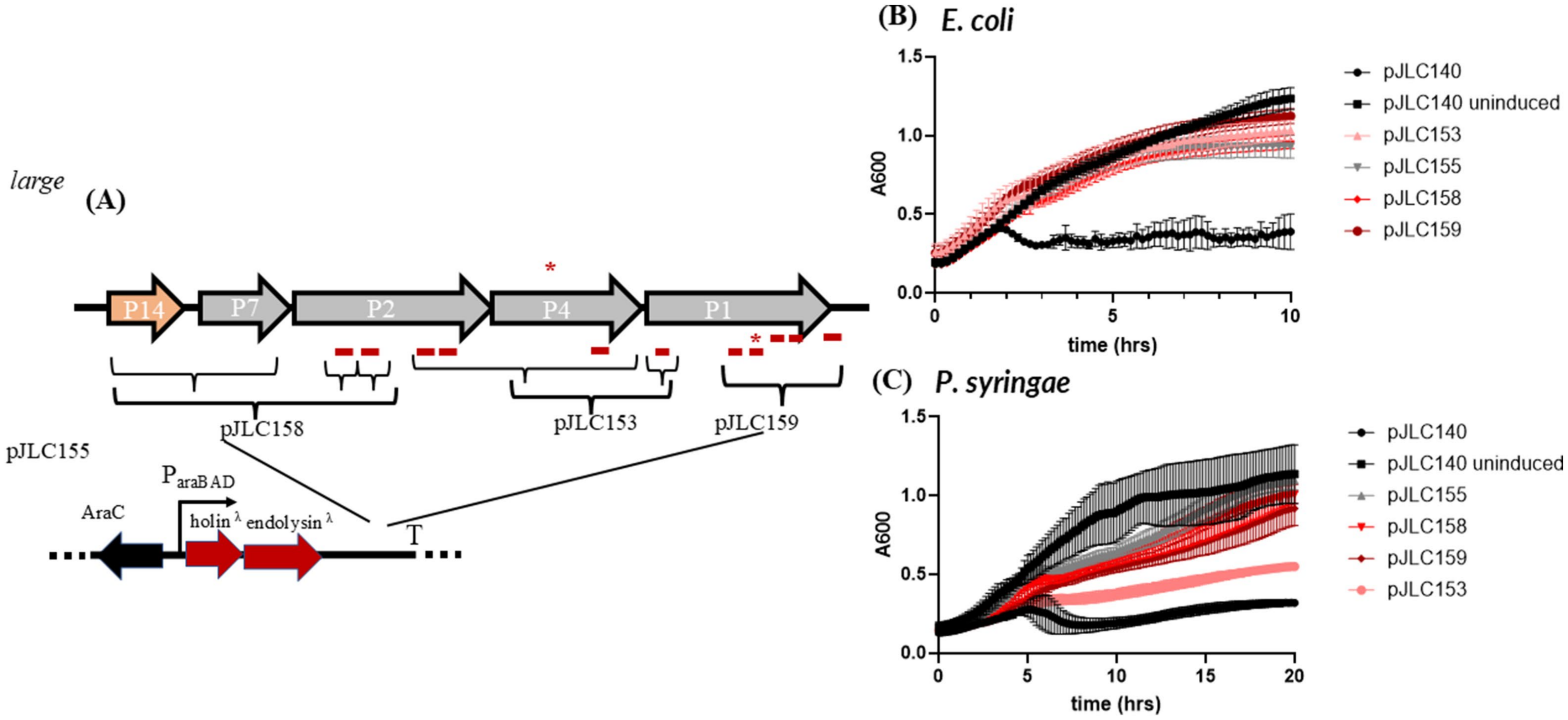
Testing outer membrane disruptor candidates from the large segment of the phi6 genome. **(A)** Shows the different combinations of candidate outer membrane disruptor genes that were cloned downstream of the lambda holin-endolysin vector, pJLC155. **(B)** Lysis profile of constructs tested in *E. coli.* **(C)** Lysis profile of constructs tested in *P. syringae.* All constructs were induced at time zero (except the uninduced control) and absorbance at 600 nm (*A*_600_) was monitored every 10 min overnight with shaking. The average of two biological replicates is shown and the error bars represent standard deviation. *TMD-containing genes.

The L segment of the phi6 genome contains 11 hypothetical genes, only one of which encodes a protein with a predicted TMD. The other TMD-containing protein in the L segment is P4, the packaging motor. The pJLC158 construct includes P14, P7 (a procapsid protein with a weak TMD prediction), and the first two hypothetical genes embedded in the +1 reading frame of P2 (replicase), which encode 66- and 65 aa proteins (Figure 5A).

The pJLC153 construct carries four hypothetical genes, including two embedded within the replicase gene (encoding 29- and 65- aa peptides), the full P4 gene sequence (which includes a 41 aa hypothetical protein), and the 5′ region of the P1 gene (capsid protein), which encodes a 29 aa peptide. At the 3′ end of the P1 gene, there are four hypothetical genes encoding proteins of 80 aa (TMD-containing), 35 aa, 120 aa, and 53 aa. A fifth hypothetical gene downstream of the P1 gene encodes a 25 aa protein; these genes are carried on the pJLC159 construct.

None of these constructs produced lysis profiles consistent with an intact lysis system in *E. coli* or *P. syringae* (Figures 5B,C). Notably, the pJLC153 construct showed evidence of toxicity, however the growth profile was distinguishable from pJLC140, which shows a marked decrease due to expression of an intact lysis cassette. Furthermore, pJLC153 and the other sets showed spherical morphology (like pJLC155) in both strains (Supplementary Figure S6), indicating that these genes are not acting as outer membrane disruptors in our testbed.

### 3.5 Overexpression of the phi6 cDNA segments shows lysis in *Escherichia coli*

Holins are known as the “lysis clock,” setting the precise timing of lysis by preventing PG degradation by the endolysin until a critical concentration is reached (Cahill and Young, 2019). In other words, holins do not form holes in the IM until this accumulation reaches the critical concentration (Cahill et al., 2024). A plausible explanation for the lack of phi6 holin function in our study is that the expression systems used may be underexpressing the holin, preventing it from reaching the critical concentration required for activation.

For *P. syringae*, the available options for inducible expression systems and replicons are relatively limited compared to those for *E. coli*. For the latter, we selected the low-copy p15A replicon paired with the arabinose-inducible promoter based on previous studies demonstrating success with low-copy expression systems paired with tight, inducible promoters (Chamakura et al., 2017). However, a limitation of this approach is the assumption that phi6 holin operates independently of other phi6 components. While holins in *Caudovirales* model systems, such as the lambda lysis system, are known to function independently of virion assembly, phi6 differs significantly from *Caudovirales* due to its status as an enveloped phage. One notable distinction is that phi6 recruits lipids from the IM during virion assembly (Mäntynen et al., 2023; Lyytinen et al., 2019). Our data above indicates low level holin function in *P. syringae* when co-expressed with genes predicted to code for TMD-containing proteins (Figure 2D). It is possible that holin function may be tied to the assembly process. Specifically, the critical concentration dependence of the phi6 holin could be influenced by the activity of assembly components such as P9 and P12, which scavenge the IM for phi6 particle formation (Johnson 3rd and Mindich, 1994; Lyytinen et al., 2019). This interplay between assembly and holin function may explain the lack of observed holin activity in our experiments.

**Figure 6:**
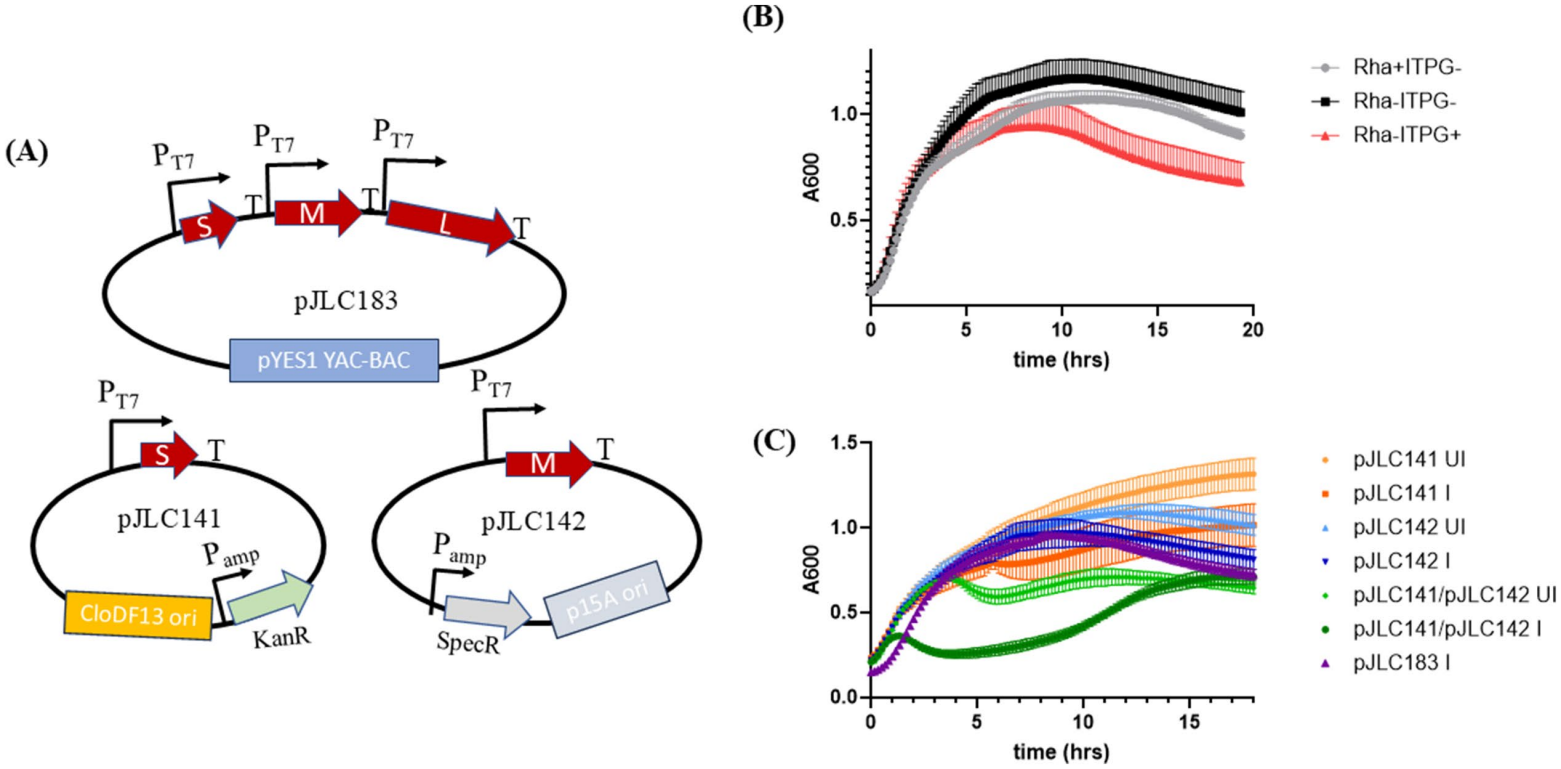
Testing the effect of Phi6 cDNA genomic segment overexpression on lysis in the absence of Mg^++^ supplementation. **(A)** Plasmid maps of constructs used in the lysis curve experiments. **(B)** Lysis profile of construct pJLC183, which carries the Phi6 S, M, and L genomic segments under control of the T7 promoter, expressed in *E. coli* Lemo21(DE3). **(C)** Lysis profiles of Lemo21(DE3) strains carrying plasmids expressing Phi6 S, M, S-M, and S-M-L genomic segments. All constructs were induced at time zero (except the uninduced control), and absorbance at 600 nm (*A*_600_) was monitored every 10 min overnight with shaking. The average of three biological replicates is shown in panel B, and the average of two biological replicates is shown in panel C. Error bars represent standard deviation.

To test whether induction strength affects phi6 holin function, we constructed plasmid pJLC183 (Figure 6A), which contains each segment of the phi6 genome as cDNA under T7 promoters. This approach simultaneously evaluates whether a stronger expression system enables phi6 holin function and whether co-expression of phi6 components influences holin activity. For expression, we used strain Lemo21(DE3), a derivative of BL21(DE3) that allows controlled expression of LysS, an inhibitor of T7 RNA polymerase, via a rhamnose-inducible system. This setup reduces leaky expression of genes under the control of the T7 promoter (Schlegel et al., 2012).

We tested three experimental conditions: (1) cultures supplemented with rhamnose during subculture without IPTG induction, (2) cultures without rhamnose supplementation and uninduced, and (3) cultures without rhamnose supplementation but fully induced with IPTG. For these sets, we withheld magnesium supplementation to media to reduce the stringency of the assay. The role of magnesium in these assays is discussed above and elsewhere (Berry et al., 2012; Cahill et al., 2017; Cahill et al., 2024). The fully induced cultures exhibited improved lysis relative to the other conditions, although the lysis profile was less sharp compared to the lambda system observed above (Figure 6B). Microscopy revealed a significant increase in the number of ghost cells (Cahill et al., 2024) relative to live cells (Supplementary Figure S7), confirming that the observed lysis profile reflects true lysis rather than toxicity.

**Figure 7:**
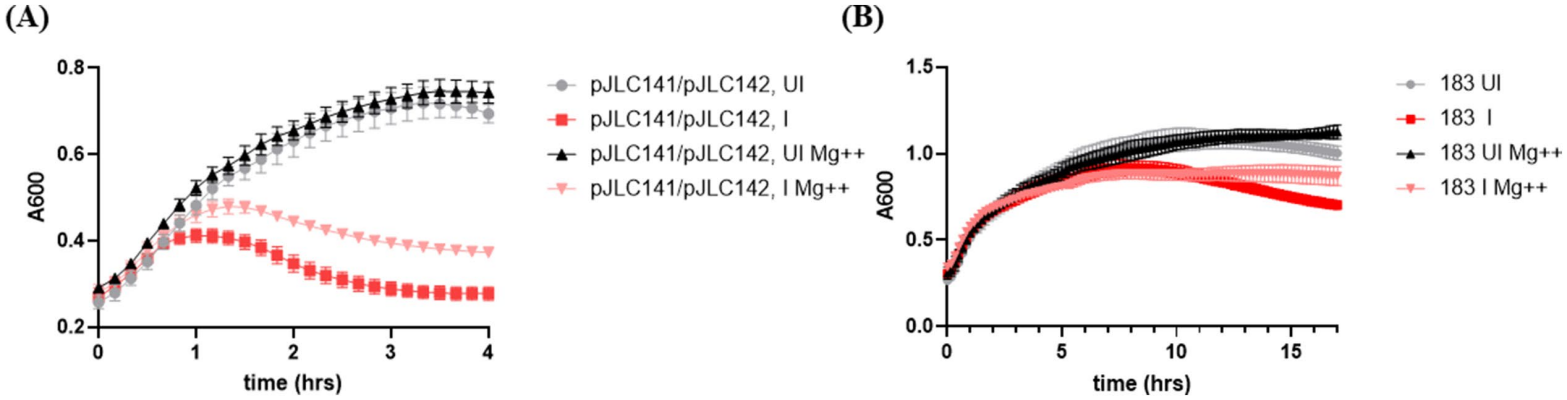
Effect of Phi6 cDNA genomic segment overexpression and Mg^++^ supplementation on lysis. Lysis profiles of *E. coli* Lemo21(DE3) strains carrying plasmids expressing the indicated Phi6 genomic segments. Cultures with magnesium supplementation are denoted as “Mg^++^.” Induction status is indicated as follows: UI = uninduced (no rhamnose in subculture, no IPTG induction), I = induced (no rhamnose supplementation in subculture, IPTG induction at time zero). **(A)** Lysis profiles of strains overexpressing the Phi6 S and M genomic segments. **(B)** Lysis profiles of strains overexpressing the Phi6 S, M, and L genomic segments. The average of two independent biological replicates is shown, and error bars represent standard deviation.

To further investigate the role of Phi6 genome segments in lysis, we tested combinations of plasmids carrying the S segment (pJLC141), M segment (pJLC142), or both. Our data indicate that both the S and M segments are required for lysis (Figure 6C), consistent with their encoding of holin-endolysin components. When cultures were supplemented with Mg++ and tested under identical conditions, we observed complete lysis in cultures without Mg++ supplementation (Figure 7). In contrast, cultures with Mg++ supplementation did not exhibit complete lysis, both in the case of S-M segment overexpression (Figure 7A) and overexpression of the entire Phi6 genome (S-M-L) (Figure 7B). These cultures displayed phenotypes and cell morphology (Supplementary Figure S8) consistent with holin-endolysin activity in the absence of an outer membrane disruptor.

Together, these findings suggest that the Phi6 lysis system requires high expression levels and/or accessory components encoded within the Phi6 genome to exhibit lysis activity, at least in *E. coli*. Furthermore, the incomplete lysis phenotype observed in magnesium-supplemented media indicates that either the outer membrane disruptor is not active in *E. coli* or Phi6 does not encode an outer membrane disruptor.

## 4 Conclusion and future directions

In this report, we investigate the lysis system of RNA phage phi6, a member of *Cystoviridae* family that encodes a holin and endolysin, resembling the lysis systems of tailed DNA phages. Using genetic complementation experiments and systematic testing of phi6 and lambda lysis components in *E. coli* and *P. syringae*, our data indicate that the phi6 endolysin is active in both bacterial systems, whereas the phi6 holin does not activate unless overexpressed or co-expressed with other phi6 genes (Figures 2D, 6). Overexpression is typically avoided in physiological studies, especially in the case of toxic and TMD-containing proteins (Schlegel et al., 2012; Wagner et al., 2008), like lysis proteins. However, we resorted to overexpression after more conventional approaches failed to identify an outer membrane disruptor or show phi6 holin function. Our data indicate that overexpression is required for lysis by phi6 components in our *E. coli* testbed. Future work could leverage our phi6 cDNA genome overexpression platforms to systematically delete phi6 genes and identify if there are other genes required for holin function.

We observed that overexpression of the S, M, and L segments (pJLC183) showed a much greater interval of time between induction and lysis. Additionally, the lysis profile of pJLC183 did not show a sharp reduction in A600 during lysis, compared to overexpression of S-M segments (Figure 6C). The simplest explanation for the difference is the addition of the L segment to S-M overexpression (pJLC183) imposes a burden on the cell that results in reduced expression of the M segment, which carries the holin gene. Such a scenario would reduce holin accumulation over time and if phi6 holin operates like model holin systems, would delay the time that the holin reaches a critical concentration (Cahill et al., 2024).

Our overexpression data (Figures 6, 7) indicates that the outer membrane disruptor is either absent from the Phi6 genome or not active in the *E. coli* overexpression testbed. If the Phi6 genome contained an active outer membrane disruptor, the lysis profiles of cation-supplemented cultures would be expected to match those of cation-free cultures, as demonstrated previously (Berry et al., 2012). Instead, we observed incomplete lysis in the presence of Mg++ (Figure 7), suggesting that the Phi6 genomic cDNA platforms do not encode an active outer membrane disruptor.

It is intriguing to consider the possibility that Phi6 has not evolved an outer membrane disruptor. In this respect, Phi6 would resemble other RNA phages, which do not carry a dedicated lysis gene for outer membrane disruption. For most known RNA phages, inhibition of peptidoglycan synthesis eventually leads to lysis and release of phage progeny. Phi6 may employ a hybrid strategy, targeting the IM and PG layer while relying on the eventual destabilization of the OM for release. However, as discussed above, this approach likely incurs fitness costs, compared to lysis systems that carry an outer membrane disruptor.

We also wish to note that we attempted to move our phi6 cDNA genome overexpression platform into *P. syringae.* However, this presented several challenges, and we report four key observations and suppositions based on these preliminary studies, which may serve as valuable insights for future investigations: (1) The pLM1086 plasmid, which has been shown to express cDNA components of phi6 under the control of the T7 promoter in previous studies (Yang, 2024), is a large plasmid, and we could not introduce it into *P. syringae* through standard electroporation protocols. (2) Conjugal transfer of pLM1086 from *E. coli* MFDPir (Jackson et al., 2020) to *P. syringae* was successful and robust; however, we were unable to introduce pJLC183 into MFDPir by electroporation, despite comparable plasmid sizes (23.16 kbp for pJLC183, and 23.06 kbp for pLM1086) and equivalent concentrations of fresh plasmid preps performed on days when pLM1086 transformation was successful. (3) Even if pJLC183 and pLM1086 could be combined to produce phi6 from a cDNA platform, pJLC183 would likely be unstable due to the simultaneous production of phi6 particles and lysis gene expression. This instability could potentially be circumvented using a suicide vector approach. Interestingly, previous reports indicated that three plasmids lacking a *P. syringae* replicon were successfully transformed simultaneously into a strain carrying pLM1086, resulting in plaque formation; however, supporting data for this observation was not provided (Sun et al., 2004). (4) To validate pLM1086 in *P. syringae*, we constructed a shuttle vector with T7-controlled GFP. While GFP expression was observed in *E. coli* strains containing the reporter/T7 system combination and in *P. syringae*, the 2-plasmid system was highly toxic to *P. syringae*. Therefore, this approach would not be successful for lysis gene overexpression in *P. syringae.* This suggests that an orthogonal strong inducer system should be explored for future studies.

## Data Availability

The datasets presented in this study will be made available by the authors upon request. The DNA sequences used in this study can be found in the article.
