# Supplementary material for: Assessment of the phi6 lysis system using genetic complementation and heterologous expression in Escherichia coli and Pseudomonas syringae
Source: Front Microbiol. 2026 Jan 7;16:1718418. doi: 10.3389/fmicb.2025.1718418 (PMC12819732; doi:10.3389/fmicb.2025.1718418)
Supplement: Supplementary file 1 [file Data_Sheet_1.pdf]

# Supplementary Figures

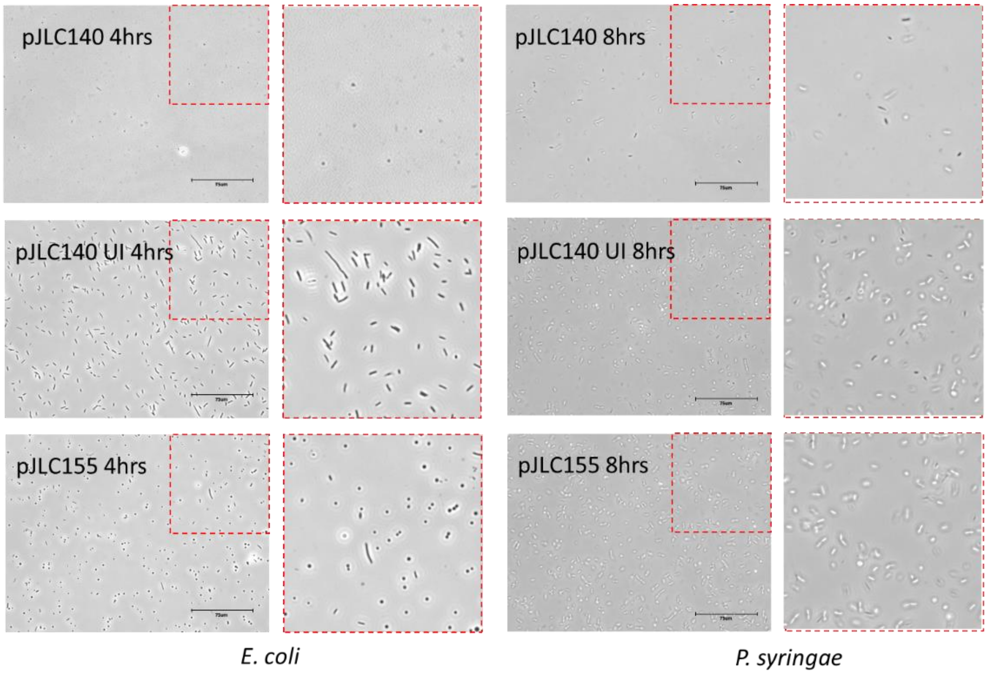


Figure S1. Micrographs of *E. coli* and *P. syringae* controls imaged at the indicated time after induction

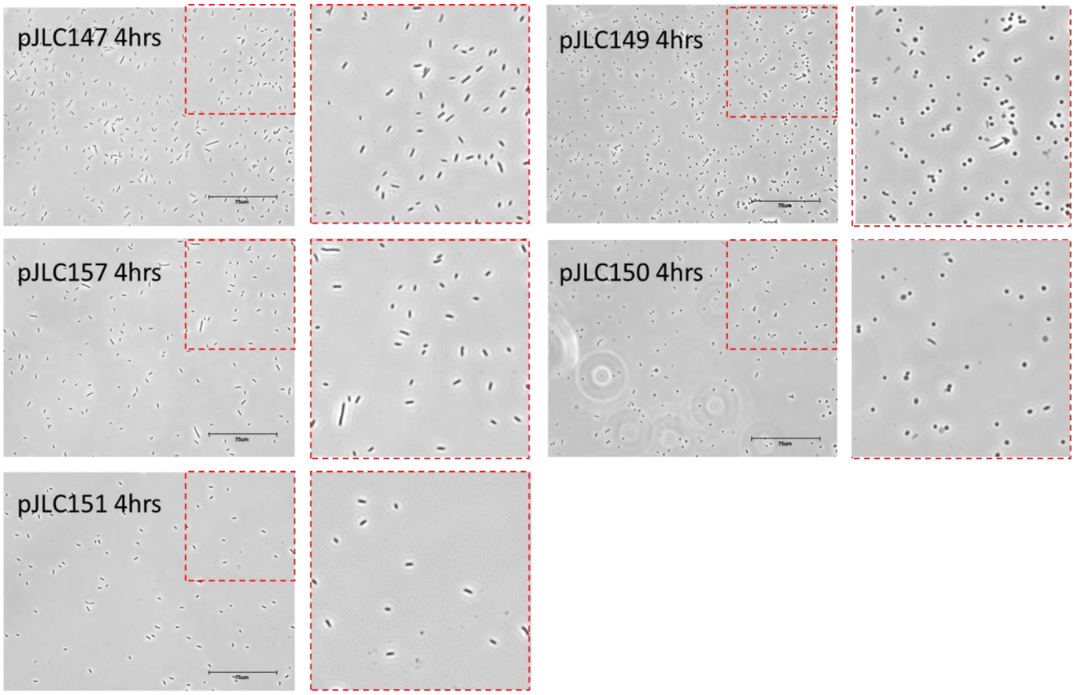


Figure S2. Micrographs of *E. coli* cultures imaged at the indicated time after induction

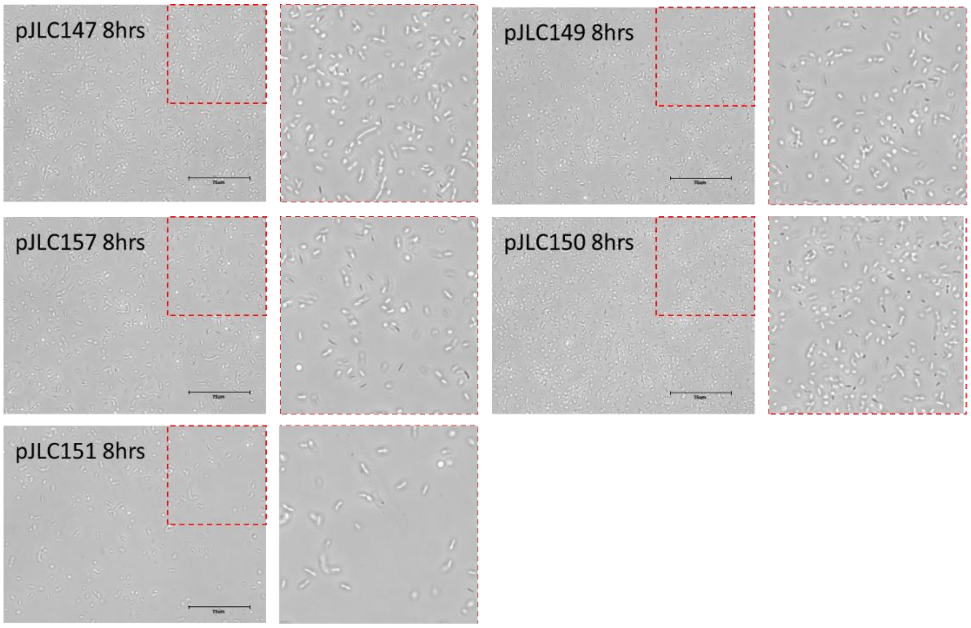


Figure S3. Micrographs of *P. syringae* cultures imaged at the indicated time after induction.

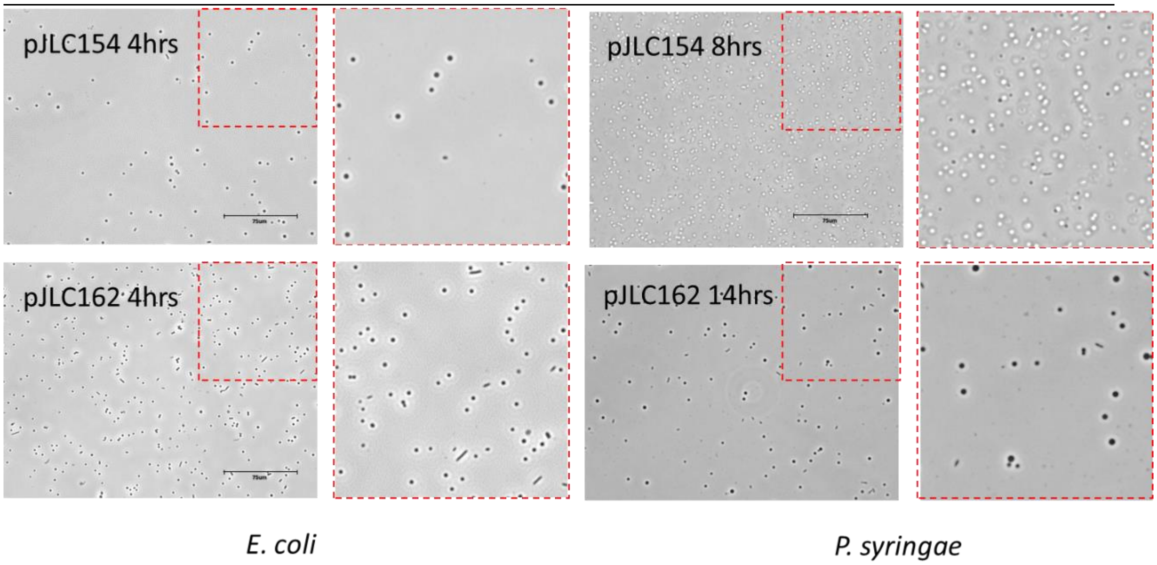


Figure S4. Micrographs of *E. coli* and *P. syringae* cultures imaged at the indicated time after induction. Note that pJLC162 was collected at 14 hours after induction, however the spherical cell morphology indicates unambiguously this construct does not carry an outer membrane disruptor.

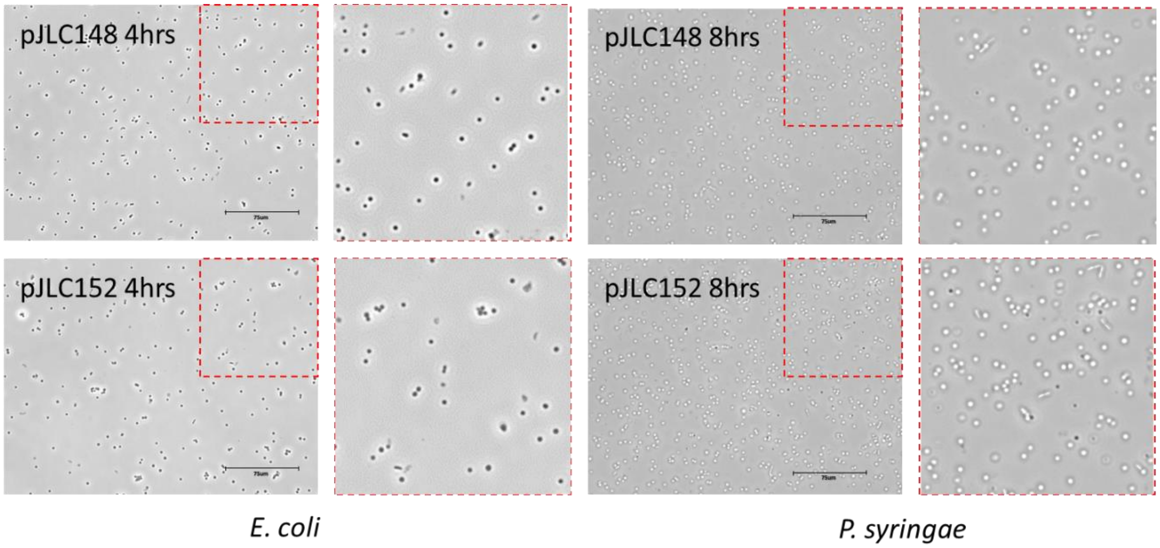


Figure S5. Micrographs of *E. coli* and *P. syringae* cultures imaged at the indicated time after induction.

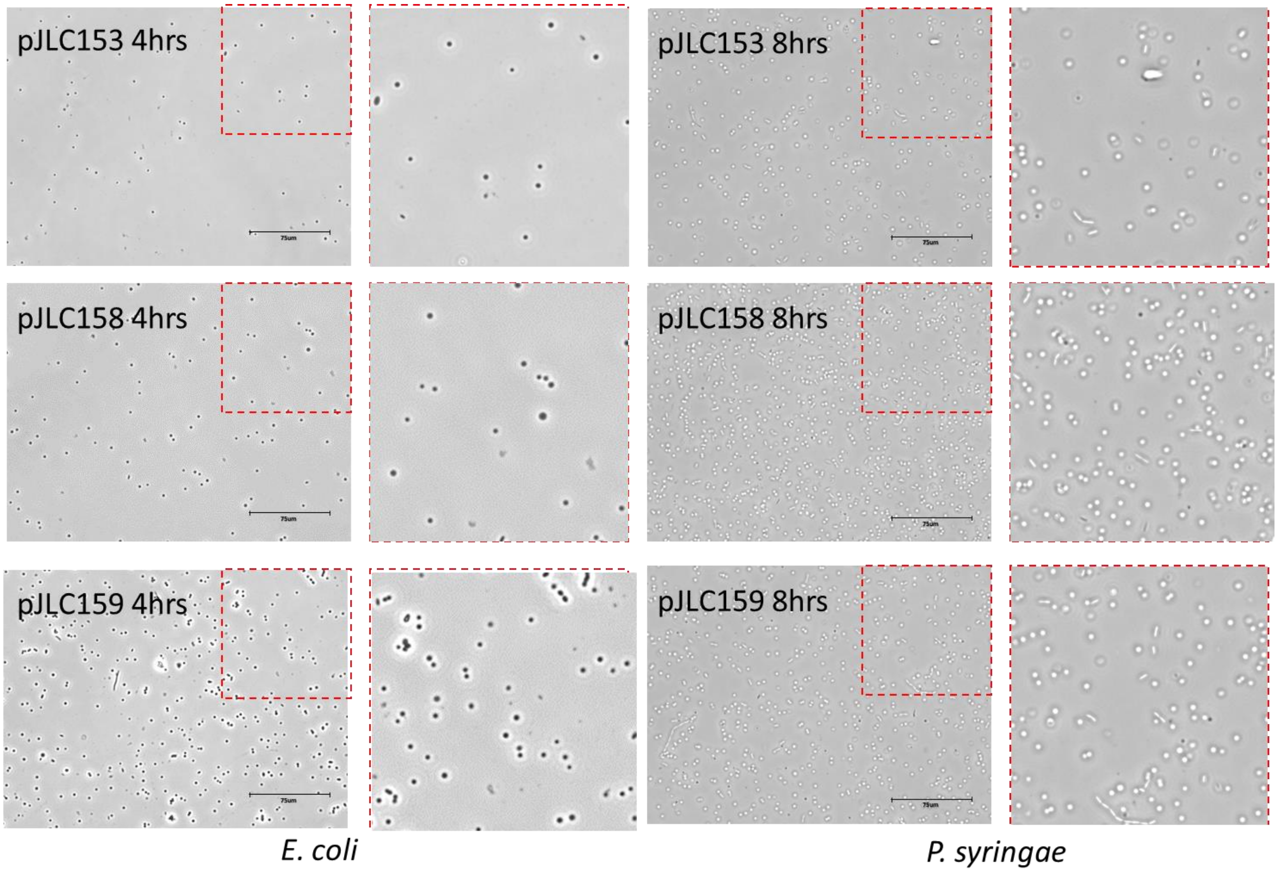


Figure S6. Micrographs of *E. coli* and *P. syringae* cultures imaged at the indicated time after induction.

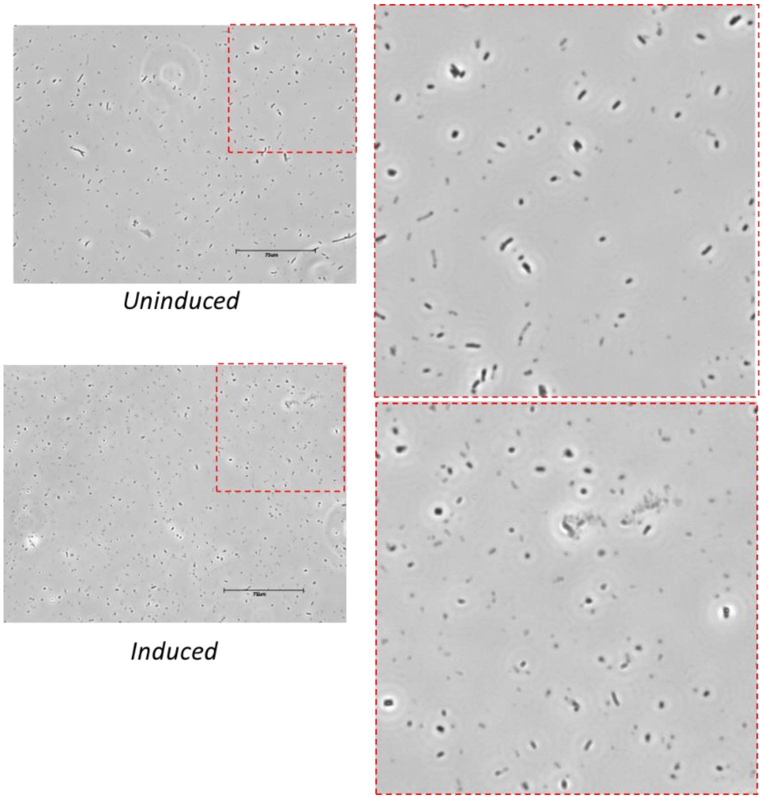


**Figure S7.** Micrographs of Lemo21(DE3) expressing pJLC183, carrying the phi6 cDNA genome segments under control of the T7 promoter.

## Supplementary Table 1. Primer names and sequences

| primer name | sequence |
|---|---|
| #642 pYES1L_FW | ACGAATTGTTAGACACCTCGCCGCAGTTAATTAAAGTCA |
| #643 pYES1L_RV | TATTACCGGATGTCCGCGGCGGTATCAGCGCGGCCGCCG |
| #644 L_FW | CGCTGATACCGCCGCGGACATCCGGTAATACGACTCACT |
| #645 L_RV | CACCCTGAATTGACTCACACAGGAAACAGCTATGACCAT |
| #646 M_FW | GCTGTTTCCTGTGTGAGTCAATTCAGGGTGGTGAATGGA |
| #647 M_RV | CTAGAGCGGGCGCAAAGTCAATTCAGGGTGGTGAATGGA |
| #648 S_FW | CACCCTGAATTGACTTTGCGCCCGCTCTAGATAATACGT |
| #649 S_RV | TTAACTGCGGCGAGGTGTCTAACAATTCGTTCAAGCCGA |
| #181 P-ara for | GTCCACATTGATTATTTGCACGGC |
| #658 Pro1600 oriV rev | GGTATCGAGGCGGATTCACATGATG |

## DNA sequences of constructs used in this study

**>pJLC140 (shuttle vector containing lysis cassette and BsmBI restriction sites enabling Golden Gate Cloning)**

Amp promoter, SpecR resistance gene, **T7 terminator**, p15A ori, *araC*, AraBAD promoter, 5’ BsmBI restriction-cut site, **lambda lysis cassette**, 3’ BsmBI restriction-cut site, BBa_B0014 double terminator, Pro1600 replicon,

CTGAGTTTGTTTATTTTTCTAAATACATTCAAATATGTATCCGCTCATGAGACAATAACCCTGA
TAAATGCTTCAATAATATTGAAAAAGGAAGAGTATGAGGGAAGCGGTGATCGCCGAAGTATCGA
CTCAACTATCAGAGGTAGTTGGCGTCATCGAGCGCCATCTCGAACCGACGTTGCTGGCCGTACA
TTTGTACGGCTCCGCAGTGGATGGCGGCCTGAAGCCACACAGTGATATTGATTTGCTGGTTACG
GTGACCGTAAGGCTTGATGAAACAACGCGGCGAGCTTTGATCAACGACCTTTTGGAAACTTCGG
CTTCCCCTGGAGAGAGCGAGATTCTCCGCGCTGTAGAAGTCACCATTGTTGTGCACGACGACAT
CATTCCGTGGCGTTATCCAGCTAAGCGCGAACTGCAATTTGGAGAATGGCAGCGCAATGACATT
CTTGCAGGTATCTTCGAGCCAGCCACGATCGACATTGATCTGGCTATCTTGCTGACAAAAGCAA
GAGAACATAGCGTTGCCTTGGTAGGTCCAGCGGCGGAGGAACTCTTTGATCCGGTTCCTGAACA
GGATCTATTTGAGGCGCTAAATGAAACCTTAACGCTATGGAACTCGCCGCCCGACTGGGCTGGC
GATGAGCGAAATGTAGTGCTTACGTTGTCCCGCATTTGGTACAGCGCAGTAACCGGCAAAATCG
CGCCGAAGGATGTCGCTGCCGACTGGGCAATGGAGCGCCTGCCGGCCCAGTATCAGCCCGTCAT
ACTTGAAGCTAGACAGGCTTATCTTGGACAAGAAGAAGATCGCTTGGCCTCGCGCGCAGATCAG
TTGGAAGAATTTGTCCACTACGTGAAAGGCGAGATCACCAAGGTAGTCGGCAAATAAT**CAAAAA**
**ACCCCTCAAGACCCGTTTAGAGGCCCCAAGGGGTTATGCTAG**CAAGCACTAGTAACAACTTATA
TCGTATGGGGCTGACTTCAGGTGCTACATTTGAAGAGATAAATTGCACTGAAATCTAGTAATAT
TTTATCTGATTAATAAGATGATCTTCTTGAGATCGTTTTGGTCTGCGCGTAATCTCTTGCTCTG
AAAACGAAAAAACCGCCTTGCAGGGCGGTTTTTCGAAGGTTCTCTGAGCTACCAACTCTTTGAA
CCGAGGTAACTGGCTTGGAGGAGCGCAGTCACCAAAACTTGTCCTTTCAGTTTAGCCTTAACCG
GCGCATGACTTCAAGACTAACTCCTCTAAATCAATTACCAGTGGCTGCTGCCAGTGGTGCTTTT
GCATGTCTTTCCGGGTTGGACTCAAGACGATAGTTACCGGATAAGGCGCAGCGGTCGGACTGAA
CGGGGGGTTCGTGCATACAGTCCAGCTTGGAGCGAACTGCCTACCCGGAACTGAGTGTCAGGCG
TGGAATGAGACAAACGCGGCCATAACAGCGGAATGACACCGGTAAACCGAAAGGCAGGAACAGG
AGAGCGCACGAGGGAGCCGCCAGGGGGAAACGCCTGGTATCTTTATAGTCCTGTCGGGTTTCGC
CACCACTGATTTGAGCGTCAGATTTCGTGATGCTTGTCAGGGGGGCGGAGCCTATGGAAAAACG
GCTTTGCCGCGGCCCTCTCACTTCCCTGTTAAGTATCTTCCTGGCATCTTCCAGGAAATCTCCG
CCCCGTTCGTAAGCCATTTCCGCTCGCCGCAGTCGAACGACCGAGCGTAGCGAGTCAGTGAGCG
AGGAAGCGGAATATATCCCTAGGTCTAGGGCGGCGGATGAAGCGTTAAGGT*TTATGACAACTTG*
*ACGGCTACATCATTCACTTTTTCTTCACAACCGGCACGGAACTCGCTCGGGCTGGCCCCGGTGC*
*ATTTTTTAAATACCCGCGAGAAATAGAGTTGATCGTCAAAACCAACATTGCGACCGACGGTGGC*
*GATAGGCATCCGGGTGGTGCTCAAAAGCAGCTTCGCCTGGCTGATACGTTGGTCCTCGCGCCAG*
*CTTAAGACGCTAATCCCTAACTGCTGGCGGAAAAGATGTGACAGACGCGACGGCGACAAGCAAA*
*CATGCTGTGCGACGCTGGCGATATCAAAATTGCTGTCTGCCAGGTGATCGCTGATGTACTGACA*
*AGCCTCGCGTACCCGATTATCCATCGGTGGATGGAGCGACTCGTTAATCGCTTCCATGCGCCGC*
*AGTAACAATTGCTCAAGCAGATTTATCGCCAGCAGCTCCGAATAGCGCCCTTCCCCTTGCCCGG*
*CGTTAATGATTTGCCCAAACAGGTCGCTGAAATGCGGCTGGTGCGCTTCATCCGGGCGAAAGAA*
*CCCCGTATTGGCAAATATTGACGGCCAGTTAAGCCATTCATGCCAGTAGGCGCGCGGACGAAAG*
*TAAACCCACTGGTGATACCATTCGCGAGCCTCCGGATGACGACCGTAGTGATGAATCTCTCCTG*
*GCGGGAACAGCAAAATATCACCCGGTCGGCAAACAAATTCTCGTCCCTGATTTTTCACCACCCC*
*CTGACCGCGAATGGTGAGATTGAGAATATAACCTTTCATTCCCAGCGGTCGGTCGATAAAAAAA*
*TCGAGATAACCGTTGGCCTCAATCGGCGTTAAACCCGCCACCAGATGGGCATTAAACGAGTATC*

*CCGGCAGCAGGGGATCATTTTGCGCTTCAGCCAT*ACTTTTCATACTCCCGCCATTCAGAGAAGA
AACCAATTGTCCATATTGCATCAGACATTGCCGTCACTGCGTCTTTTACTGGCTCTTCTCGCTA
ACCAAACCGGTAACCCCGCTTATTAAAAGCATTCTGTAACAAAGCGGGACCAAAGCCATGACAA
AAACGCGTAACAAAAGTGTCTATAATCACGGCAGAAAAGTCCACATTGATTATTTGCACGGCGT
CACACTTTGCTATGCCATAGCATTTTTATCCATAAGATTAGCGGATCCTACCTGACGCTTTTTA
TCGCAACTCTCTACTGTTTCTCCATTTGGATCAACAGGATT**GATCCGAGACGT**AATTAAATAGA
GCAAATCCCCTTATTGGGGGTAAGAC**ATGAAGATGCCAGAAAAACATGACCTGTTGGCCGCCAT**
**TCTCGCGGCAAAGGAACAAGGCATCGGGGCAATCCTTGCGTTTGCAATGGCGTACCTTCGCGGC**
**AGATATAATGGCGGTGCGTTTACAAAAACAGTAATCGACGCAACGATGTGCGCCATTATCGCCT**
**GGTTCATTCGTGACCTTCTCGACTTCGCCGGACTAAGTAGCAATCTCGCTTATATAACGAGCGT**
**GTTTATCGGCTACATCGGTACTGACTCGATTGGTTCGCTTATCAAACGCTTCGCTGCTAAAAAA**
**GCCGGAGTAGAAGATGGTAGAAATCAATAATCAACGTAAGGCGTTCCTCGATATGCTGGCGTGG**
**TCGGAGGGAACTGATAACGGACGTCAGAAAACCAGAAATCATGGTTATGACGTCATTGTAGGCG**
**GAGAGCTATTTACTGATTACTCCGATCACCCTCGCAAACTTGTCACGCTAAACCCAAAACTCAA**
**ATCAACAGGCGCCGGACGCTACCAGCTTCTTTCCCGTTGGTGGGATGCCTACCGCAAGCAGCTT**
**GGCCTGAAAGACTTCTCTCCGAAAAGTCAGGACGCTGTGGCATTGCAGCAGATTAAGGAGCGTG**
**GCGCTTTACCTATGATTGATCGTGGTGATATCCGTCAGGCAATCGACCGTTGCAGCAATATCTG**
**GGCTTCACTGCCGGGCGCTGGTTATGGTCAGTTCGAGCATAAGGCTGACAGCCTGATTGCAAAA**
**TTCAAAGAAGCGGGCGGAACGGTCAGAGAGATTGATGTATGAGCAGAGTCACCGCGATTATCTC**
**CGCTCTGGTTATCTGCATCATCGTCTGCCTGTCATGGGCTGTTAATCATTACCGTGATAACGCC**
**ATTACCTACAAAGCCCAGCGCGACAAAAATGCCAGAGAACTGAAGCTGGCGAACGCGGCAATTA**
**CTGACATGCAGATGCGTCAGCGTGATGTTGCTGCGCTCGATGCAAAATACACGAAGGAGTTAGC**
**TGATGCTAAAGCTGAAAATGATGCTCTGCGTGATGATGTTGCCGCTGGTCGTCGTCGGTTGCAC**
**ATCAAAGCAGTCTGTCAGTCAGTGCGTGAAGCCACCACCGCCTCCGGCGTGGATAATGCAGCCT**
**CCCCCCGACTGGCAGACACCGCTGAACGGGATTATTTCACCCTCAGAGAGAGGCTGATCACTAT**
**GCAAAAACAACTGGAAGGAACCCAGAAGTATATTAATGAGCAGTGCAGATAG**CGTCTCATAAAT
CAATAATCACACTGGCTCACCTTCGGGTGGGCCTTTCTGCGTTTATATACTAGAGAGAGAATAT
AAAAAGCCAGATTATTAATCCGGCTTTTTTATTATTTCCCATCATGTGAATCCGCCTCGATACC
CTGACTACTCGCTTCCTGCGCCCTCTCAGGCGGCGATAGGGGACTGGTAAAACGGGGATTGCCC
AGACGCCTCCCCCGCCCCTTCAGGGGCACAAATGCGGCCCCAACGGGGCCACGTAGTGGTGCGT
TTTTTGCGTTTCCACCCTTTTCTTCCTTTTCCCTTTTAAACCTTTTAGGACGTCTACAGGCCAC
GTAATCCGTGGCCTGTAGAGTTTAAAAAGGGACGGATTTGTTGCCATTAAGGGACGGATTTGTT
GTTAAGAAGGGACGGATTTGTTGTTGTAAAGGGACGGATTTGTTGTATTGTGGGACGCAGATAC
AGTGTCCCCTTATACACAAGGAATGTCGAACGTGGCCTCACCCCCAATGGTTTACAAAAGCAAT
GCCCTGGTCGAGGCCGCGTATCGCCTCAGTGTTCAGGAACAGCGGATCGTTCTGGCCTGTATTA
GCCAGGTGAAGAGGAGCGAGCCTGTCACCGATGAAGTGATGTATTCAGTGACGGCGGAGGACAT
AGCGACGATGGCGGGTGTCCCTATCGAATCTTCCTACAACCAGCTCAAAGAAGCGGCCCTGCGC
CTGAAACGGCGGGAAGTCCGGTTAACCCAAGAGCCCAATGGCAAGGGGAAAAGACCGAGTGTGA
TGATTACCGGCTGGGTGCAAACAATCATCTACCGGGAGGGTGAGGGCCGTGTAGAACTCAGGTT
CACCGAAGACATGCTGCCGTACCTGACGGAACTCACCAAACAGTTCACCAAATACGCCTTGGCT
GACGTGGCCAAGATGGACAGCACCCACGCGATCAGGCTTTACGAGCTGCTCATGCAATGGGACA
GCATCGGCCAGCGCGAAATAGAAATTGACCAGCTGCGAAAGTGGTTTCAACTGGAAGGCCGGTA
TCCCTCGATCAAGGACTTCAAGTTGCGAGTGCTTGATCCAGCCGTGACGCAGATCAACGAGCAC
AGCCCGCTACAGGTGGAGTGGGCGCAGCGAAAGACCGGGCGCAAGGTCACACATCTGTTGTTCA
GTTTTGGACCGAAGAAGCCCGCCAAGGCGGTGGGTAAGGCCCCAGCGAAGCGCAAGGCCGGGAA
GATTTCAGATGCTGAGATCGCGAAACAGGCTCGCCCTGGTGAGACATGGGAAGCGGCCCGCGCT
CGACTAACCCAGATGCCGCTGGATCTGGCCTAGAGGCCGTGGCCACCACGGCCCGGCCTGCCTT

TCAGGCTGCGCAACTGTTGGGAAGGGCGATCGGTGCGGGCCTCTTCGCTATTACGCGTCGACCG
ATGCCCTTGAGAGCCTTCAACCCAGTCAGCTCCTTCCGGTGGGCGCGGGGCATGACTATCGTCG
CCGCACTTATGACTGTCTTCTTTATCATGCAACTCGTAGGACAGGTGCCGGCAGCGCTCTGGGT
CATTTTCGGCGAGGACCGCTTTCGCTGGAGCGCGACGATGATCGGCCTGTCGCTTGCGGTATTC
GGAATCTTGCACGCCCTCGCTCAAGCCTTCGTCACTGGTCCCGCCACCAAACGTTTCGGCGAGA
AGCAGGCCATTATCGCCT

**>pJLC147 gblock insert**

TCATAGGTCTCCGTAGGGTTCTATCAGTAATCGACCTTATTCCCGTCTCTGATCCTAATTAAAT
AGAGCAAATCCCCTTATTGGGGGTAAGACAATAAGAGATCCATTCAATGGACAACATCCTCGAT
CCCCTTAAGGCTCCGTTTTCTTCGGAAGCCGCCGCGAAAACCACCGCTGCCAAAATCGCTGTGG
TATACGCGTTGGTCGGTCTGGTTGGCGGTCTGCTGCTCACCAAGTAATGGCCTAACTCCTCGTG
TCCAAGGATAGCGCCTTCGCAGTGCAATACTCGCTGCGCGCCCTGGGACAAAAGGTGCGGGCAG
ACGGGGTAGTGGGCTCTGAAACCCGTGCCGCGCTGGATGCGCTGCCCGAGAATCAGAAGAAAGC
GATTGTAGAGTTGCAAGCACTCCTACCGAAAGCACAGTCGGTCGGCAACAACCGTGTGAGGTTC
ACAACAGCTGAAGTCGACTCGGCGGTGGCGCGGATCTCGCAAAAGATAGGTGTTCCGGCTTCCT
ACTACCAGTTCCTGATTCCGATCGAGAACTTCGTGGTGGCCGGTGGTTTCGAAACCACCGTTTC
TGGTTCCTTCCGTGGGTTGGGCCAGTTCAACCGGCAGACGTGGGATAGACTCCGTCGTTTAGGC
CGTAACCTTCCTGCATTTGAGGAGGGTTCGGCACAACTGAACGCTTCTCTTTATGCAATCGGGT
TCTTGTATCTTGAGAACAAGAGAGCGTACGAGGCGTCGTTCAAAGGCCGCGTTTTCACTCACGA
AATCGCGTATTTGTATCACAACCAAGGCGCTCCAGCTGCCGAACAGTACCTGACTTCGGGTCGG
CTCGTTTACCCGAAGCAAAGCGAGGCCGCTGTCGCGGGCGTTGCGGCTGCGAGAAACCAGCATG
TCAAAGAGAGTTGGGCTTAGGCTAGTAGGGGTCTTCCACCGCGATTATCTCCGCTCTGGTTATG
AAGACCACAATATAGATAAAGAGACGCAATAGAGACCGTCA

**>pJLC148 gblock insert**

TCATAGGTCTCCGTAGGGTTCTATCAGTAATCGACCTTATTCCCGTCTCTGATCCTAATTAAAT
AGAGCAAATCCCCTTATTGGGGGTAAGACATGAAGATGCCAGAAAAACATGACCTGTTGGCCGC
CATTCTCGCGGCAAAGGAACAAGGCATCGGGGCAATCCTTGCGTTTGCAATGGCGTACCTTCGC
GGCAGATATAATGGCGGTGCGTTTACAAAAACAGTAATCGACGCAACGATGTGCGCCATTATCG
CCTGGTTCATTCGTGACCTTCTCGACTTCGCCGGACTAAGTAGCAATCTCGCTTATATAACGAG
CGTGTTTATCGGCTACATCGGTACTGACTCGATTGGTTCGCTTATCAAACGCTTCGCTGCTAAA
AAAGCCGGAGTAGAAGATGGTAGAAATCAATAATCAACGTAAGGCGTTCCTCGATATGCTGGCG
TGGTCGGAGGGAACTGATAACGGACGTCAGAAAACCAGAAATCATGGTTATGACGTCATTGTAG
GCGGAGAGCTATTTACTGATTACTCCGATCACCCTCGCAAACTTGTCACGCTAAACCCAAAACT
CAAATCAACAGGCGCCGGACGCTACCAGCTTCTTTCCCGTTGGTGGGATGCCTACCGCAAGCAG
CTTGGCCTGAAAGACTTCTCTCCGAAAAGTCAGGACGCTGTGGCATTGCAGCAGATTAAGGAGC
GTGGCGCTTTACCTATGATTGATCGTGGTGATATCCGTCAGGCAATCGACCGTTGCAGCAATAT
CTGGGCTTCACTGCCGGGCGCTGGTTATGGTCAGTTCGAGCATAAGGCTGACAGCCTGATTGCA
AAATTCAAAGAAGCGGGCGGAACGGTCAGAGAGATTGATGTATGAGCTAATGAGCTAGTAGGGG
TCTTCGAGAGATTGATGTATGCATGACACGCGACCGCTCCGAACCGAGAGGCCCATGGCCAGCA
AGAATACGAATGACCGGGTTTTCGACCGGTTTCATTCTTCTCTCGTTCGGCTGTGGCATTCTGC
AAACCAGCGCATGCGCGGTTCTTTCTCCGTCGTGGTTCGGGTCGAGCACTCTTTAGTGCTCCTC
ATCGGCTACACGGTGGTAGGCGCGACTGTCGCACACTTCGTGAGGTGACTATGTTAGCTTTCGT
AGCGCGAGCGGTCGTACTTTACTCTGCTGGTGTAGTCGTGGGCATCGCCTACGATCACGTCACA

GGAAGGAAACGTCGCCAATGACTAAGTGGAAGATGTACATCGCCGGCGTCGTTCTGGTCATCGT
AGGGGCAGTTACTCATGCTCCACAGCTGATGGTCCAGGGCATGACTACGCTCGCGACTCAAGCG
GCCGCAGACGCGGCCGATGGTGGAGGTGCTCAGTGAGAAGACCACAATATAGATAAATGAGACG
CAATAGAGACCGTCA

**>pJLC149 gblock insert**

TCATAGGTCTCCGTAGGGTTCTATCAGTAATCGACCTTATTCCCGTCTCTGATCCTAATTAAAT
AGAGCAAATCCCCTTATTGGGGGTAAGACATGAAGATGCCAGAAAAACATGACCTGTTGGCCGC
CATTCTCGCGGCAAAGGAACAAGGCATCGGGGCAATCCTTGCGTTTGCAATGGCGTACCTTCGC
GGCAGATATAATGGCGGTGCGTTTACAAAAACAGTAATCGACGCAACGATGTGCGCCATTATCG
CCTGGTTCATTCGTGACCTTCTCGACTTCGCCGGACTAAGTAGCAATCTCGCTTATATAACGAG
CGTGTTTATCGGCTACATCGGTACTGACTCGATTGGTTCGCTTATCAAACGCTTCGCTGCTAAA
AAAGCCGGAGTAGAAGATGGTAGAAATCAATAATGGCCTAACTCCTCGTGTCCAAGGATAGCGC
CTTCGCAGTGCAATACTCGCTGCGCGCCCTGGGACAAAAGGTGCGGGCAGACGGGGTAGTGGGC
TCTGAAACCCGTGCCGCGCTGGATGCGCTGCCCGAGAATCAGAAGAAAGCGATTGTAGAGTTGC
AAGCACTCCTACCGAAAGCACAGTCGGTCGGCAACAACCGTGTGAGGTTCACAACAGCTGAAGT
CGACTCGGCGGTGGCGCGGATCTCGCAAAAGATAGGTGTTCCGGCTTCCTACTACCAGTTCCTG
ATTCCGATCGAGAACTTCGTGGTGGCCGGTGGTTTCGAAACCACCGTTTCTGGTTCCTTCCGTG
GGTTGGGCCAGTTCAACCGGCAGACGTGGGATAGACTCCGTCGTTTAGGCCGTAACCTTCCTGC
ATTTGAGGAGGGTTCGGCACAACTGAACGCTTCTCTTTATGCAATCGGGTTCTTGTATCTTGAG
AACAAGAGAGCGTACGAGGCGTCGTTCAAAGGCCGCGTTTTCACTCACGAAATCGCGTATTTGT
ATCACAACCAAGGCGCTCCAGCTGCCGAACAGTACCTGACTTCGGGTCGGCTCGTTTACCCGAA
GCAAAGCGAGGCCGCTGTCGCGGGCGTTGCGGCTGCGAGAAACCAGCATGTCAAAGAGAGTTGG
GCTTAGGCTAGTAGGGGTCTTCCACCGCGATTATCTCCGCTCTGGTTATGAAGACCACAATATA
GATAAATGAGACGCAATAGAGACCGTCA

**>pJLC150 gblock insert**

TCATAGGTCTCCGTAGGGTTCTATCAGTAATCGACCTTATTCCCGTCTCTGATCCTAATTAAAT
AGAGCAAATCCCCTTATTGGGGGTAAGACATGAAGATGCCAGAAAAACATGACCTGTTGGCCGC
CATTCTCGCGGCAAAGGAACAAGGCATCGGGGCAATCCTTGCGTTTGCAATGGCGTACCTTCGC
GGCAGATATAATGGCGGTGCGTTTACAAAAACAGTAATCGACGCAACGATGTGCGCCATTATCG
CCTGGTTCATTCGTGACCTTCTCGACTTCGCCGGACTAAGTAGCAATCTCGCTTATATAACGAG
CGTGTTTATCGGCTACATCGGTACTGACTCGATTGGTTCGCTTATCAAACGCTTCGCTGCTAAA
AAAGCCGGAGTAGAAGATGGTAGAAATCAATAAAGGAGGTATACAATGTGTCCAAGGATAGCGC
CTTCGCAGTGCAATACTCGCTGCGCGCCCTGGGACAAAAGGTGCGGGCAGACGGGGTAGTGGGC
TCTGAAACCCGTGCCGCGCTGGATGCGCTGCCCGAGAATCAGAAGAAAGCGATTGTAGAGTTGC
AAGCACTCCTACCGAAAGCACAGTCGGTCGGCAACAACCGTGTGAGGTTCACAACAGCTGAAGT
CGACTCGGCGGTGGCGCGGATCTCGCAAAAGATAGGTGTTCCGGCTTCCTACTACCAGTTCCTG
ATTCCGATCGAGAACTTCGTGGTGGCCGGTGGTTTCGAAACCACCGTTTCTGGTTCCTTCCGTG
GGTTGGGCCAGTTCAACCGGCAGACGTGGGATAGACTCCGTCGTTTAGGCCGTAACCTTCCTGC
ATTTGAGGAGGGTTCGGCACAACTGAACGCTTCTCTTTATGCAATCGGGTTCTTGTATCTTGAG
AACAAGAGAGCGTACGAGGCGTCGTTCAAAGGCCGCGTTTTCACTCACGAAATCGCGTATTTGT
ATCACAACCAAGGCGCTCCAGCTGCCGAACAGTACCTGACTTCGGGTCGGCTCGTTTACCCGAA
GCAAAGCGAGGCCGCTGTCGCGGGCGTTGCGGCTGCGAGAAACCAGCATGTCAAAGAGAGTTGG

GCTTAGGCTAGTAGGGGTCTTCCACCGCGATTATCTCCGCTCTGGTTATGAAGACCACAATATA
GATAAATGAGACGCAATAGAGACCGTCA

**>pJLC151 gblock insert**

TCATAGGTCTCCGTAGGGTTCTATCAGTAATCGACCTTATTCCCGTCTCTGATCCTAATTAAAT
AGAGCAAATACCATGAGCGCGGTCTATTTGAGAGTGTCGCTCTTCTGCCTACGCGCTCATTCGT
TCCCTCGAGTTGACGCTTCAAGCAGGTGGACACCTCCTCAACCCATAATAAGAGATCCATTCAA
TGGACAACATCCTCGATCCCCTTAAGGCTCCGTTTTCTTCGGAAGCCGCCGCGAAAACCACCGC
TGCCAAAATCGCTGTGGTATACGCGTTGGTCGGTCTGGTTGGCGGTCTGCTGCTCACCAAGTAA
GGTGTAGTATGCATGACACGCGACCGCTCCGAACCGAAAGACCCATGGCCAGCAAGAATACGAA
TGACCGGGTTTTCGACCGGTTTCATTCTTCTCTCGTTCGGCTGTGGCATTCTGCAAACCAGCGC
ATGCGCGGTTCTTTCTCCGTCGTGGTTCGGGTCGAGCACTCTTTAGTGCTCCTCATCGGCTACA
CGGTGGTAGGCGCGACTGTCGCACACTTCGTGAGGTGACTATGTTAGCTTTCGTAGCGCGAGCG
GTCGTACTTTACTCTGCTGGTGTAGTCGTGGGCATCGCCTACGATCACGTCACAGGAAGGAAAC
GTCGCCATGACTAAGTGGAAGATGTACATCGCCGGCGTCGTTCTGGTCATCGTAGGGGCAGTTA
CTCATGCTCCACAGCTGATGGTCCAGGGCATGACTACGCTCGCGACTCAAGCGGCCGCAGACGC
GGCCGATGGTGGAGGTGCTCA
GTGAGTATCTTCTCCTCGTTGTTCAAGGTCATCAAGAAGGTAATCTCGAAGGTGGTCGCCACCC
TTAAGAAAATCTTCAAGAAGATCTGGCCGTTGCTACTTATTGTGGCAATTATCTACTTCGCTCC
CTACCTCGCCGGGTTCTTCACTTCCGCCGGGTTCACTGGGATCGGAGGGATCTTCTCCTCTATC
GCAACCACCATCACGCCTACGCTGACGTCGTTCCTGTCGACTGCGTGGTCTGGTGTGGGCTCTC
TTGCCTCCACGGCTTGGTCTGGGTTCCAATCTCTCGGGATGGGTACTCAGCTCGCTGTCGTGAG
TGGCGCGGCTGCTCTGATTGCACCTGAGGAAACGGCTCAACTGGTTACCGAAATCGGTACCACC
GTAGGTGATATCGCCGGTACGATTATCGGCGGTGTCGCCAAGGCACTCCCGGGTTGGATCTGGA
TCGCCGCAGGCGGTCTTGCCGTCTGGGCCCTCTGGCCGTCATCTGACAGTAAGGAGTAGCAAAT
GCGCTACCAAGGCATCAACGAGTGGCTGGGTGGAGCCAAGAAACTCACCACCGCAAACGGTGAG
ATTGGCGCTATCTACCTCTCCGCTGCTCCTCCCACCGACGCCGCACGTGCGGACGCTAAGGCGG
TGGATTTTACTGCTGGTTGGCCAAGCGCGATCGTTGACTGCGCTGATGCCACTCGTGCCAAGCA
GAACTACCTGTGGGTTGGCGATAACGTTGTGCACATCGGGGCTAAACACGTTCCACTCCTCGAT
CTGTGGGGCGGGACAGGTGATGCCTGGCAGCAGTTCGTTGGCTATGCCTGCCCAATGCTCGACC
TTTGTCGTGCGTGGGGCCTGGGTTATGCCAGCGCTTCTGTAACCACCGGCTCGTTGCAGGGCTA
TCAGCCATCGGCGTTCTTGGACGAGTAGAAGATGGTAGAAATCAATAATCAACGTAAGGCGTTC
CTCGATATGCTGGCGTGGTCGGAGGGAACTGATAACGGACGTCAGAAAACCAGAAATCATGGTT
ATGACGTCATTGTAGGCGGAGAGCTATTTACTGATTACTCCGATCACCCTCGCAAACTTGTCAC
GCTAAACCCAAAACTCAAATCAACAGGCGCCGGACGCTACCAGCTTCTTTCCCGTTGGTGGGAT
GCCTACCGCAAGCAGCTTGGCCTGAAAGACTTCTCTCCGAAAAGTCAGGACGCTGTGGCATTGC
AGCAGATTAAGGAGCGTGGCGCTTTACCTATGATTGATCGTGGTGATATCCGTCAGGCAATCGA
CCGTTGCAGCAATATCTGGGCTTCACTGCCGGGCGCTGGTTATGGTCAGTTCGAGCATAAGGCT
GACAGCCTGATTGCAAAATTCAAAGAAGCGGGCGGAACGGTCAGAGAGATTGATGTATGAGCTA
GTAGGGGTCTTCCACCGCGATTATCTCCGCTCTGGTTATGAAGACCACAATATAGATAAATGAG
ACGCAATAGAGACCGTCA

**>pJLC152 gblock insert**

TCATAGGTCTCCGTAGGGTTCTATCAGTAATCGACCTTATTCCCGTCTCTGATCCTAATTAAAT
AGAGCAAATCCCCTTATTGGGGGTAAGACATGAAGATGCCAGAAAAACATGACCTGTTGGCCGC

CATTCTCGCGGCAAAGGAACAAGGCATCGGGGCAATCCTTGCGTTTGCAATGGCGTACCTTCGC
GGCAGATATAATGGCGGTGCGTTTACAAAAACAGTAATCGACGCAACGATGTGCGCCATTATCG
CCTGGTTCATTCGTGACCTTCTCGACTTCGCCGGACTAAGTAGCAATCTCGCTTATATAACGAG
CGTGTTTATCGGCTACATCGGTACTGACTCGATTGGTTCGCTTATCAAACGCTTCGCTGCTAAA
AAAGCCGGAGTAGAAGATGGTAGAAATCAATAATCAACGTAAGGCGTTCCTCGATATGCTGGCG
TGGTCGGAGGGAACTGATAACGGACGTCAGAAAACCAGAAATCATGGTTATGACGTCATTGTAG
GCGGAGAGCTATTTACTGATTACTCCGATCACCCTCGCAAACTTGTCACGCTAAACCCAAAACT
CAAATCAACAGGCGCCGGACGCTACCAGCTTCTTTCCCGTTGGTGGGATGCCTACCGCAAGCAG
CTTGGCCTGAAAGACTTCTCTCCGAAAAGTCAGGACGCTGTGGCATTGCAGCAGATTAAGGAGC
GTGGCGCTTTACCTATGATTGATCGTGGTGATATCCGTCAGGCAATCGACCGTTGCAGCAATAT
CTGGGCTTCACTGCCGGGCGCTGGTTATGGTCAGTTCGAGCATAAGGCTGACAGCCTGATTGCA
AAATTCAAAGAAGCGGGCGGAACGGTCAGAGAGATTGATGTATGAGCTAGTAGGGGTCTTCAAG
GCATCAACGAGTGGCTGGGTGGAGCCAAGAAACTCACCACCGCAAACGGTGAGATTGGCGCTAT
CTACCTCTCCGCTGCTCCTCCCACCGACGCCGCACGTGCGGACGCTAAGGCGGTGGATTTTACT
GCTGGTTGGCCAAGCGCGATCGTTGACTGCGCTGATGCCACTCGTGCCAAGCAGAACTACCTGT
GGGTTGGCGATAACGTTGTGCACATCGGGGCTAAACACGTTCCACTCCTCGATCTGTGGGGCGG
GACAGGTGATGCCTGGCAGCAGTTCGTTGGCTATGCCTGCCCAATGCTCGACCTTTGTCGTGCG
TGGGGCCTGGGTTATGCCAGCGCTTCTGTAATCCTCAATCTCATCGGGAAGTGACCATGGTACC
GCTAAAAATTAGCACGCTGGAGTCCCAGCTGCAACCGCTTGTTAAGTTGGTTGCAACCGAAACC
CCCGGTGCCCTCGTAGCGTATGCTCGAGGGTTATCGAGTGCCGACCGCTCGCGGTTGTACAGAC
TGCTTCGTTCTTTGGAGCAGGCCATCCCGAAGCTGTCGTCGGCTGTCGTTTCGGCCACGACGTT
GGCAGCGCGAGGTCTTTAATGGAAACCAACCCGCTGCTTCAGCTTGAGTCGCTGTCGTTACGCT
TGCGAGACATGCCTCGTTCGCGCCTTTCTGCGCTGATGAAGAACATGTCGTATGAGCAGCTGCA
GTCGTTGTATAGCACCAGCGTAAAAGTTGGCGCTGTGCTCGATAGCGTTTCAATGCAGTTGCTT
GAGGCGTCACAAACCGCTCAATCGGGAACTCGACTGATGACACCGCAGGAGTACGTCGCTGCTG
GTGGAGGTCGTGTGTACGTTAAATAAGTCCTTAGATTTCTAAGGCGAGACTCGCTTTGCGAGCA
TCCAATAGGATGGCCCCTTCGGGGGCTCTCTCTCTGAAGACCACAATATAGATAAATGAGACGC
AATAGAGACCGTCA

**>pJLC153 gblock insert**

TCATAGGTCTCCGTAGGGTTCTATCAGTAATCGACCTTATTCCCGTCTCTGATCCTAATTAAAT
AGAGCAAATCCCCTTATTGGGGGTAAGACATGAAGATGCCAGAAAAACATGACCTGTTGGCCGC
CATTCTCGCGGCAAAGGAACAAGGCATCGGGGCAATCCTTGCGTTTGCAATGGCGTACCTTCGC
GGCAGATATAATGGCGGTGCGTTTACAAAAACAGTAATCGACGCAACGATGTGCGCCATTATCG
CCTGGTTCATTCGTGACCTTCTCGACTTCGCCGGACTAAGTAGCAATCTCGCTTATATAACGAG
CGTGTTTATCGGCTACATCGGTACTGACTCGATTGGTTCGCTTATCAAACGCTTCGCTGCTAAA
AAAGCCGGAGTAGAAGATGGTAGAAATCAATAATCAACGTAAGGCGTTCCTCGATATGCTGGCG
TGGTCGGAGGGAACTGATAACGGACGTCAGAAAACCAGAAATCATGGTTATGACGTCATTGTAG
GCGGAGAGCTATTTACTGATTACTCCGATCACCCTCGCAAACTTGTCACGCTAAACCCAAAACT
CAAATCAACAGGCGCCGGACGCTACCAGCTTCTTTCCCGTTGGTGGGATGCCTACCGCAAGCAG
CTTGGCCTGAAAGACTTCTCTCCGAAAAGTCAGGACGCTGTGGCATTGCAGCAGATTAAGGAGC
GTGGCGCTTTACCTATGATTGATCGTGGTGATATCCGTCAGGCAATCGACCGTTGCAGCAATAT
CTGGGCTTCACTGCCGGGCGCTGGTTATGGTCAGTTCGAGCATAAGGCTGACAGCCTGATTGCA
AAATTCAAAGAAGCGGGCGGAACGGTCAGAGAGATTGATGTATGACTAGTAGGGGTCTTCCTAC
GAGCACGGTGGCGCCTTCCTTGGTGACATCCTGCTTTACGACTCGCGTCGTGAGCCTGGCTCTG
CCATCTTCGTTGGTAACATCAACTCAATGCTGAACAACCAGTTCAGCCCTGAGTACGGTGTCCA

ATCGGGCGTTCGCGACCGATCTAAGCGCAAACGGCCGTTCCCCGGTCTTGCTTGGGCGTCGATG
AAAGATACCTACGGTGCCTGTCCGATCTACTCTGATGTGCTGGAGGCGATCGAGCGTTGCTGGT
GGAACGCGTTCGGTGAGTCGTACCGTGCGTATCGTGAAGATATGCTTAAACGCGACACTCTCGA
ACTATCACGCTACGTTGCGTCGATGGCTCGTCAAGCCGGGCTGGCTGAACTCACTCCCATTGAT
TTGGAGGTGCTTGCTGACCCGAACAAACTCCAGTATAAGTGGACCGAGGCCGATGTCTCGGCGA
ATATCCACGAGGTACTGATGCATGGCGTATCGGTCGAAAAGACTGAGCGCTTTCTCCGTTCTGT
AATGCCGAGGTAATCTGCCGATTGTCGTAACTCAAGCGCATATTGATCGTGTCGGCATCGCCGC
CGATCTGCTCGATGCGTCTCCTGTGTCGCTTCAAGTTCTTGGTCGCCCTACCGCGATCAACACT
GTCGTCATCAAGACGTACATCGCTGCTGTTATGGAGCTCGCCTCCAAGCAAGGTGGTTCGTTGG
CCGGTGTGGATATTCGTCCTTCGGTTCTGCTGAAAGACACCGCTATCTTCACCAAGCCGAAGGC
GAAGTCCGCTGACGTCGAATCTGATGTCGACGTACTGGACACGGGGATTTACTCCGTTCCTGGA
CTGGCTCGCAAGCCTGTCACCCACCGTTGGCCATCAGAGGGTATCTACTCTGGTGTCACAGCTC
TGATGGGCGCTACCGGTTCCGGTAAGTCGATCACGCTGAACGAAAAGCTCCGTCCAGACGTCCT
GATTCGTTGGGGCGAGGTGGCTGAAGCTTACGATGAGCTGGATACCGCCGTCCACATCTCGACT
CTGGATGAGATGTTGATTGTGTGTATTGGCCTGGGTGCACTCGGGTTCAACGTCGCTGTTGACT
CGGTTCGTCCTCTGCTGTTCCGTCTCAAAGGCGCCGCCTCTGCGGGGGGTATTGTGGCTGTGTT
CTACAGCCTGTTGACCGATATCTCGAACTTGTTCACACAATACGATTGCTCTGTCGTCATGGTC
GTTAACCCGATGGTTGACGCTGAGAAGATCGAGTACGTGTTCGGTCAGGTCATGGCTTCGACTG
TCGGTGCGATCTTGTGTGCTGATGGCAACGTGTCTAGAACGATGTTCCGGACCAACAAAGGTCG
TATTTTCAACGGTGCGGCCCCTCTTGCTGCTGACACTCACATGCCTAGCATGGATCGTCCTACC
AGCATGAAGGCCCTCGATCATACCTCGATCGCCTCTGTCGCACCGCTGGAGCGTGGCTCCGTGG
ATACCGACGATCGCAATTCCGCTCCGCGCCGTGGCGCTAACTTCTCTCTGTAACCAGCGAGTTG
CTTTGGGAAGTGGGCAAGGGCAACATCGACCCAGTGATGTACGCTCGTCTGTTTTTCCAGTACG
CGCAAGCTGGCGGCGCTCTGTCCGTTGAGAAGACCACAATATAGATAAAGAGACGCAATAGAGA
CCGTCA

**>pJLC154 gblock insert**

TCATAGGTCTCCGTAGGGTTCTATCAGTAATCGACCTTATTCCCGTCTCTGATCCTAATTAAAT
AGAGCAAATCCCCTTATTGGGGGTAAGACATGAAGATGCCAGAAAAACATGACCTGTTGGCCGC
CATTCTCGCGGCAAAGGAACAAGGCATCGGGGCAATCCTTGCGTTTGCAATGGCGTACCTTCGC
GGCAGATATAATGGCGGTGCGTTTACAAAAACAGTAATCGACGCAACGATGTGCGCCATTATCG
CCTGGTTCATTCGTGACCTTCTCGACTTCGCCGGACTAAGTAGCAATCTCGCTTATATAACGAG
CGTGTTTATCGGCTACATCGGTACTGACTCGATTGGTTCGCTTATCAAACGCTTCGCTGCTAAA
AAAGCCGGAGTAGAAG*A*TGGTAGAAATCAATAATCAACGTAAGGCGTTCCTCGATATGCTGGCG
TGGTCGGAGGGAACTGATAACGGACGTCAGAAAACCAGAAATCATGGTTATGACGTCATTGTAG
GCGGAGAGCTATTTACTGATTACTCCGATCACCCTCGCAAACTTGTCACGCTAAACCCAAAACT
CAAATCAACAGGCGCCGGACGCTACCAGCTTCTTTCCCGTTGGTGGGATGCCTACCGCAAGCAG
CTTGGCCTGAAAGACTTCTCTCCGAAAAGTCAGGACGCTGTGGCATTGCAGCAGATTAAGGAGC
GTGGCGCTTTACCTATGATTGATCGTGGTGATATCCGTCAGGCAATCGACCGTTGCAGCAATAT
CTGGGCTTCACTGCCGGGCGCTGGTTATGGTCAGTTCGAGCATAAGGCTGACAGCCTGATTGCA
AAATTCAAAGAAGCGGGCGGAACGGTCAGAGAGATTGATGTATGAATGAGCTAGTAGGGGTCTT
CGAGAGATTGATGTATGGTTATCGGGCTCCTGAAGTATCTCACGCCTGCCGTTAAGGTGCAGAT
GGCTGCTCGCGCGTTGGGCCTGTCCCCCGCCGAAGTCGCTGCAATTGACGGCACGTTGGGTCGT
GTCTCTGCGATGCCAGCGGTCGCGGTCGTGCTGGGAGGGAAACCTCTCTCTCTGGCCACGATCG
CGTCAGTTGTGTCTGATGCAAACCCCAGTGCCACTGTTGGCGCGCTTATGCCTGCTGTACAGGG

CATGGTGAGTTCCGACGAAGGCGCGAGTGCGTTGGCTAAGACCGTGGTAGGCTTCATGGAGTCC
GACCCCAACAGCGATGTCCTGGTTCAACTGCTCCACAAGGTGTCAAACTTGCCGATTGTCGGCT
TTGGTGACACGCAGTATGCAGACCCAGCTGACTTCTTGGCCAAGGGAGTTTTCCCTCTGATCAG
GAAGCCAGAAGTAGAGGTTCAAGCTGCGCCTTTCACCTGTCGTCAGTGTGATCATGTTGATCAC
ATCACTGATGTACCTCAAACTTCGACCTTTGTTCACAAATGCACTTCGTGCGGCTTTGTGCAGA
TGGTCCACCGTAAGGATGTTCCGTAAATGCCATTTCCTCTGGTAAAGCAAGACCCAACCTCGAA
GGCTTTCACTGAAGCCAGTGAACGCTCCACCGGCACCCAGATCCTGGACGTCGTCAAGGCCCCT
ATCGGCCTGTTCGGCGACGATGCCAAACACGAGTTCGTGACCCGTCAGGAACAAGCCGTTTCCG
TCGTCAGCTGGGCAGTTGCTGCCGGTCTGATCGGCGAGCTGATCGGCTACCGTGGTGCGCGTTC
GGGTCGCAAAGCGATCCTGGCCAACATCCCTTTTCTGGCCTAAGAAGACCACAATATAGATAAA
TGAGACGCAATAGAGACCGTCA

**>pJLC155 gblock insert**

TCATAGGTCTCCGTAGGTTCTATCAGTAATCGACCTTATTCCCGTCTCTGATCCTAATTAAATA
GAGCAAATCCCCTTATTGGGGGTAAGACATGAAGATGCCAGAAAAACATGACCTGTTGGCCGCC
ATTCTCGCGGCAAAGGAACAAGGCATCGGGGCAATCCTTGCGTTTGCAATGGCGTACCTTCGCG
GCAGATATAATGGCGGTGCGTTTACAAAAACAGTAATCGACGCAACGATGTGCGCCATTATCGC
CTGGTTCATTCGTGACCTTCTCGACTTCGCCGGACTAAGTAGCAATCTCGCTTATATAACGAGC
GTGTTTATCGGCTACATCGGTACTGACTCGATTGGTTCGCTTATCAAACGCTTCGCTGCTAAAA
AAGCCGGAGTAGAAGATGGTAGAAATCAATAATCAACGTAAGGCGTTCCTCGATATGCTGGCGT
GGTCGGAGGGAACTGATAACGGACGTCAGAAAACCAGAAATCATGGTTATGACGTCATTGTAGG
CGGAGAGCTATTTACTGATTACTCCGATCACCCTCGCAAACTTGTCACGCTAAACCCAAAACTC
AAATCAACAGGCGCCGGACGCTACCAGCTTCTTTCCCGTTGGTGGGATGCCTACCGCAAGCAGC
TTGGCCTGAAAGACTTCTCTCCGAAAAGTCAGGACGCTGTGGCATTGCAGCAGATTAAGGAGCG
TGGCGCTTTACCTATGATTGATCGTGGTGATATCCGTCAGGCAATCGACCGTTGCAGCAATATC
TGGGCTTCACTGCCGGGCGCTGGTTATGGTCAGTTCGAGCATAAGGCTGACAGCCTGATTGCAA
AATTCAAAGAAGCGGGCGGAACGGTCAGAGAGATTGATGTATGAGCTAGTAGGGTCTTCCACCG
CGATTATCTCCGCTCTGGTTATGAAGACCACAATATAGATAAATGAGACGCAATAGAGACCGTC
A

**>pJLC157 gblock insert**

TCATAGGTCTCCGTAGGGTTCTATCAGTAATCGACCTTATTCCCGTCTCTGATCCTAATTAAAT
AGAGCAAATCCCCTTATTGGGGGTAAGACAATAAGAGATCCATTCAATGGACAACATCCTCGAT
CCCCTTAAGGCTCCGTTTTCTTCGGAAGCCGCCGCGAAAACCACCGCTGCCAAAATCGCTGTGG
TATACGCGTTGGTCGGTCTGGTTGGCGGTCTGCTGCTCACCAAGTAAGGTGTAGTATGCATGAC
ACGCGACCGCTCCGAACCGAGAGGCCCATGGCCAGCAAGAATACGAATGACCGGGTTTTCGACC
GGTTTCATTCTTCTCTCGTTCGGCTGTGGCATTCTGCAAACCAGCGCATGCGCGGTTCTTTCTC
CGTCGTGGTTCGGGTCGAGCACTCTTTAGTGCTCCTCATCGGCTACACGGTGGTAGGCGCGACT
GTCGCACACTTCGTGAGGTGACTATGTTAGCTTTCGTAGCGCGAGCGGTCGTACTTTACTCTGC
TGGTGTAGTCGTGGGCATCGCCTACGATCACGTCACAGGAAGGAAACGTCGCCATGACTAAGTG
GAAGATGTACATCGCCGGCGTCGTTCTGGTCATCGTAGGGGCAGTTACTCATGCTCCACAGCTG
ATGGTCCAGGGCATGACTACGCTCGCGACTCAAGCGGCCGCAGACGCGGCCGATGGTGGAGGTG
CTCAGTGATGGCCTAACTCCTCGTGTCCAAGGATAGCGCCTTCGCAGTGCAATACTCGCTGCGC

GCCCTGGGACAAAAGGTGCGGGCAGACGGGGTAGTGGGCTCTGAAACCCGTGCCGCGCTGGATG
CGCTGCCCGAGAATCAGAAGAAAGCGATTGTAGAGTTGCAAGCACTCCTACCGAAAGCACAGTC
GGTCGGCAACAACCGTGTGAGGTTCACAACAGCTGAAGTCGACTCGGCGGTGGCGCGGATCTCG
CAAAAGATAGGTGTTCCGGCTTCCTACTACCAGTTCCTGATTCCGATCGAGAACTTCGTGGTGG
CCGGTGGTTTCGAAACCACCGTTTCTGGTTCCTTCCGTGGGTTGGGCCAGTTCAACCGGCAGAC
GTGGGATAGACTCCGTCGTTTAGGCCGTAACCTTCCTGCATTTGAGGAGGGTTCGGCACAACTG
AACGCTTCTCTTTATGCAATCGGGTTCTTGTATCTTGAGAACAAGAGAGCGTACGAGGCGTCGT
TCAAAGGCCGCGTTTTCACTCACGAAATCGCGTATTTGTATCACAACCAAGGCGCTCCAGCTGC
CGAACAGTACCTGACTTCGGGTCGGCTCGTTTACCCGAAGCAAAGCGAGGCCGCTGTCGCGGGC
GTTGCGGCTGCGAGAAACCAGCATGTCAAAGAGAGTTGGGCTTAGGCTAGTAGGGGTCTTCCAC
CGCGATTATCTCCGCTCTGGTTATGAAGACCACAATATAGATAAATGAGACGCAATAGAGACCG
TCA

**>pJLC158 gblock insert**

TCATAGGTCTCCGTAGGGTTCTATCAGTAATCGACCTTATTCCCGTCTCTGATCCTAATTAAAT
AGAGCAAATCCCCTTATTGGGGGTAAGACATGAAGATGCCAGAAAAACATGACCTGTTGGCCGC
CATTCTCGCGGCAAAGGAACAAGGCATCGGGGCAATCCTTGCGTTTGCAATGGCGTACCTTCGC
GGCAGATATAATGGCGGTGCGTTTACAAAAACAGTAATCGACGCAACGATGTGCGCCATTATCG
CCTGGTTCATTCGTGACCTTCTCGACTTCGCCGGACTAAGTAGCAATCTCGCTTATATAACGAG
CGTGTTTATCGGCTACATCGGTACTGACTCGATTGGTTCGCTTATCAAACGCTTCGCTGCTAAA
AAAGCCGGAGTAGAAGATGGTAGAAATCAATAATCAACGTAAGGCGTTCCTCGATATGCTGGCG
TGGTCGGAGGGAACTGATAACGGACGTCAGAAAACCAGAAATCATGGTTATGACGTCATTGTAG
GCGGAGAGCTATTTACTGATTACTCCGATCACCCTCGCAAACTTGTCACGCTAAACCCAAAACT
CAAATCAACAGGCGCCGGACGCTACCAGCTTCTTTCCCGTTGGTGGGATGCCTACCGCAAGCAG
CTTGGCCTGAAAGACTTCTCTCCGAAAAGTCAGGACGCTGTGGCATTGCAGCAGATTAAGGAGC
GTGGCGCTTTACCTATGATTGATCGTGGTGATATCCGTCAGGCAATCGACCGTTGCAGCAATAT
CTGGGCTTCACTGCCGGGCGCTGGTTATGGTCAGTTCGAGCATAAGGCTGACAGCCTGATTGCA
AAATTCAAAGAAGCGGGCGGAACGGTCAGAGAGATTGATGTATGAGCTAGTAGGGGTCTTCAAT
CCCTGGAGATAACCAATGGCTACATTACAAGATGTGCATCTACGGGTGAATGACCGGGTAACAC
CG*GTGTACTTCACTGCTCGCTCGTTTCTGCTCGTTTCTCCGAAACGTGCGGGGCAAGCAACGTT*
*CCTCGCTCGCGAGGAGGGTACTGACAATCCTGTCGTTACCTGTCATGTATCCGACTTTTATAAG*
*GACGGTGTGTAA*TGACTTTGTACCTGGTCCCTCCGCTGGATTCGGCGGACAAAGAGTTGCCTGC
TCTGGCTTCCAAAGCTGGGGTAACGCTTCTCGAGATCGAGTTTCTTCACGAGCTCTGGCCTCAC
CTCAGTGGTGGTCAGATCGTGATCGCCGCTCTCAACGCCAACAATCTGGCCATCCTCAACCGTC
ACATGTCCACTCTGTTGGTCGAGTTGCCGGTTGCTGTGATGGCCGTTCCCGGTGCTAGCTATCG
TTCCGATTGGAACATGATCGCTCACGCACTCCCGTCTGAGGATTGGATCACTTTGTCCAACAAG
ATGCTGAAAAGCGGCTTGCTGGCGAACGATACCGTCCAGGGCGAGAAGCGCTCCGGCGCTGAGC
CGCTGTCGCCGAACGTGTACACCGATGCGCTCTCGCGACTCGGTATCGCGACGGCCCATGCTAT
CCCCGTTGAACCCGAACAACCGTTCGATGTCGATGAGGTAAGCGCCTGAGCTGCAAGGTAAGTT
TGATGACGCCTACCAGCTCCACCAAATGGGTGGTGCCTATTACGTCGTGTATCGTGCACAATCG
ACCGATGCTATCACACTCGACCCTAAGACCGGAAAATTCGTGTCAAAGGATCGTATGGTCGCTG
ACTTCGAATACGCAGTCACGGGCGGTGAGCAAGGCTCGCTGTTCGCTGCTTCGAAGGATGCCTC
TCGTTTGAGCGGGATCTCATCTGTGATGAACTGCTCAACATGGGGTACGCTCCGTGGTGGGTTA
AGTTGTTCGAAACCTCGCTCAAACTGCCCGTTTACGTGGGCGCTCCTGCTCCTGAGCAGGGCCA
CACGTTGTTGGGTGATCCGTCCAACCCTGATCTCGAAGTTGGGCTCTCGTCCGGACAAGGGGCG

ACCGACCTCATGGGCACGTTGCTCATGAGAAGACCACAATATAGATAAATGAGACGCAATAGAG
ACCGTCA

**>pJLC159 gblock insert**

TCATAGGTCTCCGTAGGGTTCTATCAGTAATCGACCTTATTCCCGTCTCTGATCCTAATTAAAT
AGAGCAAATCCCCTTATTGGGGGTAAGACATGAAGATGCCAGAAAAACATGACCTGTTGGCCGC
CATTCTCGCGGCAAAGGAACAAGGCATCGGGGCAATCCTTGCGTTTGCAATGGCGTACCTTCGC
GGCAGATATAATGGCGGTGCGTTTACAAAAACAGTAATCGACGCAACGATGTGCGCCATTATCG
CCTGGTTCATTCGTGACCTTCTCGACTTCGCCGGACTAAGTAGCAATCTCGCTTATATAACGAG
CGTGTTTATCGGCTACATCGGTACTGACTCGATTGGTTCGCTTATCAAACGCTTCGCTGCTAAA
AAAGCCGGAGTAGAAGATGGTAGAAATCAATAATCAACGTAAGGCGTTCCTCGATATGCTGGCG
TGGTCGGAGGGAACTGATAACGGACGTCAGAAAACCAGAAATCATGGTTATGACGTCATTGTAG
GCGGAGAGCTATTTACTGATTACTCCGATCACCCTCGCAAACTTGTCACGCTAAACCCAAAACT
CAAATCAACAGGCGCCGGACGCTACCAGCTTCTTTCCCGTTGGTGGGATGCCTACCGCAAGCAG
CTTGGCCTGAAAGACTTCTCTCCGAAAAGTCAGGACGCTGTGGCATTGCAGCAGATTAAGGAGC
GTGGCGCTTTACCTATGATTGATCGTGGTGATATCCGTCAGGCAATCGACCGTTGCAGCAATAT
CTGGGCTTCACTGCCGGGCGCTGGTTATGGTCAGTTCGAGCATAAGGCTGACAGCCTGATTGCA
AAATTCAAAGAAGCGGGCGGAACGGTCAGAGAGATTGATGTATGAGCTAGTAGGGGTCTTCTGG
GCCAGCCGAGCCATGTTGTGGTCTACGAAGACTGGCAGTTTGCCAAGGAGATCACCGCTTTCAC
TCCTGTCAAGCTGGCCAACAACTCGAATCAGCGTTTCCTGGACGTTGAGCCTGGTATCTCTGAT
CGTATGTCGGCTACGCTGGCACCAATCGGCAACACGTTCGCGGTTTCGGCGTTCGTCAAGAACC
GCACCGCCGTTTACGAGGCTGTTTCGCAGCGTGGTACAGTCAACAGCAACGGCGCGGAGATGAC
CCTCGGGTTCCCTTCCGTTGTTGAACGCGACTACGCTCTCGACCGTGACCCTATGGTCGCGATC
GCTGCTCTGCGCACTGGTATCGTCGATGAAAGTCTCGAGGCTCGCGCTTCGAACGATCTGAAAC
GGTCGATGTTCAACTACTACGCGGCTGTGATGCATTACGCTGTTGCTCACAATCCTGAAGTTGT
TGTTTCGGAGCACCAAGGTGTTGCCGCCGAACAAGGTTCGCTCTACCTGGTGTGGAACGTCCGC
ACTGAGCTGCGAATCCCTGTTGGTTACAACGCCATCGAGGGCGGTTCGATCCGTACCCCTGAGC
CGTTGGAGGCGATCGCCTACAACAAGCCGATCCAACCGTCCGAGGTGCTGCAAGCCAAGGTACT
GGATTTGGCTAACCACACAACCTCGATTCACATCTGGCCGTGGCATGAGGCTTCGACCGAGTTC
GCGTACGAAGACGCCTACTCTGTCACCATCCGCAACAAACGCTACACCGCCGAAGTCAAGGAGT
TCGAACTCCTCGGGCTCGGTCAACGTCGCGAACGTGTACGGATCCTCAAGCCTACGGTAGCCCA
CGCTATCATCCAGATGTGGTATTCCTGGTTCGTCGAGGACGACCGCACTTTGGCAGCTGCCCGT
CGCACGTCTCGCGATGACGCCGAGAAGCTTGCCATCGACGGTCGTCGTATGCAAAACGCTGTGA
CCTTGCTTCGCAAGATCGAGATGATTGGGACAACCGGTATCGGTGCGTCTGCCGTCCACCTCGC
GCAGTCGCGCATCGTGGATCAGATGGCCGGTCGAGGGCTCATCGACGACAGCTCCGATCTCCAT
GTCGGTATCAACCGTCACCGTATCCGCATCTGGGCCGGCCTCGCCGTTCTCCAGATGATGGGGC
TCTTGAGCCGCTCCGAAGCGGAAGCTCTCACCAAGGTCCTTGGTGATAGCAACGCTCTGGGCAT
GGTTGTCGCCACAACCGACATTGATCCATCCCTGTAACTCTCGTAAGCTCTCATAAGAAGACCA
CAATATAGATAAATGAGACGCAATAGAGACCGTCA

**>pJLC162 gblock insert**

TCATAGGTCTCCGTAGGGTTCTATCAGTAATCGACCTTATTCCCGTCTCTGATCCTAATTAAAT
AGAGCAAATCCCCTTATTGGGGGTAAGACATGAAGATGCCAGAAAAACATGACCTGTTGGCCGC
CATTCTCGCGGCAAAGGAACAAGGCATCGGGGCAATCCTTGCGTTTGCAATGGCGTACCTTCGC
GGCAGATATAATGGCGGTGCGTTTACAAAAACAGTAATCGACGCAACGATGTGCGCCATTATCG

CCTGGTTCATTCGTGACCTTCTCGACTTCGCCGGACTAAGTAGCAATCTCGCTTATATAACGAG
CGTGTTTATCGGCTACATCGGTACTGACTCGATTGGTTCGCTTATCAAACGCTTCGCTGCTAAA
AAAGCCGGAGTAGAAGATGGTAGAAATCAATAATCAACGTAAGGCGTTCCTCGATATGCTGGCG
TGGTCGGAGGGAACTGATAACGGACGTCAGAAAACCAGAAATCATGGTTATGACGTCATTGTAG
GCGGAGAGCTATTTACTGATTACTCCGATCACCCTCGCAAACTTGTCACGCTAAACCCAAAACT
CAAATCAACAGGCGCCGGACGCTACCAGCTTCTTTCCCGTTGGTGGGATGCCTACCGCAAGCAG
CTTGGCCTGAAAGACTTCTCTCCGAAAAGTCAGGACGCTGTGGCATTGCAGCAGATTAAGGAGC
GTGGCGCTTTACCTATGATTGATCGTGGTGATATCCGTCAGGCAATCGACCGTTGCAGCAATAT
CTGGGCTTCACTGCCGGGCGCTGGTTATGGTCAGTTCGAGCATAAGGCTGACAGCCTGATTGCA
AAATTCAAAGAAGCGGGCGGAACGGTCAGAGAGATTGATGTATGAGCTAGTAGGGGTCTTCTGT
AGTAGCCCGTGCGGCCGTCCCTGCTATTGAGAGTGCCATTGCGGCTACTCCTGGCCTGGTTTCC
CGAATCGCAGCCGCGATCGGTTCCAAGGTCAGCCCTTCCGCCATTTTGGCGGCGGTCAAGAGCA
ACCCGGTCGTCGCAGGTCTGACACTGAGAACAAGAGAGCGTACGAGGCGTCGTTCAAAGGCCGC
GTTTTCACTCACGAAATCGCGTATTTGTATCACAACCAAGGCGCTCCAGCTGCCGAACAGTACC
TGACTTCGGGTCGGCTCGTTTACCCGAAGCAAAGCGAGGCCGCTGTCGCGGCGGTTGCGGCTGC
GAGAAACCAGCATGTCAAAGAGAGTTGGGCTTAGCCCTGAACTGCATCGTGAACTGAAAATGTT
CCCAGATGTCACGAAGGGTGGCACGTTCGACATAACCATCCGGTCGACTACCGAGAACGGTGCT
TTTTGGGCGAACTACGAAGGTAGAACGTCCTTGGTCACCGTCCCGGACGTGAAGACAGCTATCG
AGTTTTTGATTAAACTCTGCCGTCGACACAAGTTGTCCAATCAGGTGAACACGCGAACGCTTCT
CCGCGATTTGCAACGAACGTTGCAGGAATGTGAATGCCAGTCTCATCATGTGCCGTTGTCCAGC
CCCTTCATGCATCTCAGATTTGCGTAAAGCTGATCGGAAGCTATGAAAGTAAGCTGAGCGACAC
GGAAGTTATTGAAGCAGCTATCAAGCTCTCATAGGCTTGGAAAGCCCGGCATCGATGTCGTTCC
ATGTCGCGCCAGACGCGGCCACCGATATGTATCTTGATCTGATCGAAATCTACTCCCCGTCGTC
AGTCGGGATACATCTCGTCCTGCCATAAGCGCTGTCTGTAGCGTGCATAAACAGATAGATCGCC
TTTTTAGGAAGACCACAATATAGATAAATGAGACGCAATAGAGACCGTCA

**>pJLC183 (phi6 cDNA genome under control of 3 T7 promoters on pYES1 BAC/YAC shuttle vector)**

CCTCGCCGCAGTTAATTAAAGTCAGTGAGCGAGGAAGCGCGTAACTATAACGGTCCTAAGGTAG
CGAATCCTGATGCGGTATTTTCTCCTTACGCATCTGTGCGGTATTTCACACCGCATAGATCGGC
AAGTGCACAAACAATACTTAAATAAATACTACTCAGTAATAACCTATTTCTTAGCATTTTTGAC
GAAATTTGCTATTTTGTTAGAGTCTTTTACACCATTTGTCTCCACACCTCCGCTTACATCAACA
CCAATAACGCCATTTAATCTAAGCGCATCACCAACATTTTCTGGCGTCAGTCCACCAGCTAACA
TAAAATGTAAGCTTTCGGGGCTCTCTTGCCTTCCAACCCAGTCAGAAATCGAGTTCCAATCCAA
AAGTTCACCTGTCCCACCTGCTTCTGAATCAAACAAGGGAATAAACGAATGAGGTTTCTGTGAA
GCTGCACTGAGTAGTATGTTGCAGTCTTTTGGAAATACGAGTCTTTTAATAACTGGCAAACCGA
GGAACTCTTGGTATTCTTGCCACGACTCATCTCCATGCAGTTGGACGATATCAATGCCGTAATC
ATTGACCAGAGCCAAAACATCCTCCTTAAGTTGATTACGAAACACGCCAACCAAGTATTTCGGA
GTGCCTGAACTATTTTTATATGCTTTTACAAGACTTGAAATTTTCCTTGCAATAACCGGGTCAA
TTGTTCTCTTTCTATTGGGCACACATATAATACCCAGCAAGTCAGCATCGGAATCTAGAGCACA
TTCTGCGGCCTCTGTGCTCTGCAAGCCGCAAACTTTCACCAATGGACCAGAACTACCTGTGAAA
TTAATAACAGACATACTCCAAGCTGCCTTTGTGTGCTTAATCACGTATACTCACGTGCTCAATA
GTCACCAATGCCCTCCCTCTTGGCCCTCTCCTTTTCTTTTTTCGACCGAATTAATTCTTAATCG
GCAAAAAAAGAAAAGCTCCGGATCAAGATTGTACGTAAGGTGACAAGCTATTTTTCAATAAAGA
ATATCTTCCACTACTGCCATCTGGCGTCATAACTGCAAAGTACACATATATTACGATGCTGTTC
TATTAAATGCTTCCTATATTATATATATAGTAATGTCGTGATCTATGGTGCACTCTCAGTACAA

TCTGCTCTGATGCCGCATAGTTAAGCCAGCCCCGACACCCGCCAACACCCGCTGACGCGCCCTG
ACGGGCTTGTCTGCTCCCGGCATCCGCTTACAGACAAGCTGTGACCGTCTCCGGGAGCTGCATG
TGTCAGAGGTTTTCACCGTCATCACCGAAACGCGCGAGACGAAAGGGCCTCGTGATACGCCTAT
TTTTATAGGTTAATGTCATGATAATAATGGTTTCTTAGACGGATCGCTTGCCTGTAACTTACAC
GCGCCTCGTATCTTTTAATGATGGAATAATTTGGGAATTTACTCTGTGTTTATTTATTTTTATG
TTTTGTATTTGGATTTTAGAAAGTAAATAAAGAAGGTAGAAGAGTTACGGAATGAAGAAAAAAA
AATAAACAAAGGTTTAAAAAATTTCAACAAAAAGCGTACTTTACATATATATTTATTAGACAAG
AAAAGCAGATTAAATAGATATACATTCGATTAACGATAAGTAAAATGTAAAATCACAGGATTTT
CGTGTGTGGTCTTCTACACAGACAAGGTGAAACAATTCGGCATTAATACCTGAGAGCAGGAAGA
GCAAGATAAAAGGTAGTATTTGTTGGCGATCCCCCTAGAGTCTTTTACATCTTCGGAAAACAAA
AACTATTTTTTCTTTAATTTCTTTTTTTACTTTCTATTTTTAATTTATATATTTATATTAAAAA
ATTTAAATTATAATTATTTTTATAGCACGTGATGAAAAGGACCCAGGTGGCACTTTTCGGGGAA
ATGTGCGCGGAACCCCTATTTGTTTATTTTTCTAAATACATTCAAATATGTATCCGCTCATGAG
ACAATAACCCTGATAAATGCTTCAATAATATTGAAAAAGGAAGAGTATGAGTATTCAACATTTC
CGTGTCGCCCTTATTCCCTTTTTTGCGGCATTTTGCCTTCCTGTTTTTGCTCACCCAGAAACGC
TGGTGAAAGTAAAAGATGCTGAAGATCAGTTGGGACGCGTAGTCTAGACCAGCCAGGACAGAAA
TGCCTCGACTTCGCTGCTACCCAAGGTTGCCGGGTGACGCACACCGTGGAAACGGATGAAGGCA
CGAACCCAGTGGACATAAGCCTGTTCGGTTCGTAAGCTGTAATGCAAGTAGCGTATGCGCTCAC
GCAACTGGTCCAGAACCTTGACCGAACGCAGCGGTGGTAACGGCGCAGTGGCGGTTTTCATGGC
TTGTTATGACTGTTTTTTTGGGGTACAGTCTATGCCTCGGGCATCCAAGCAGCAAGCGCGTTAC
GCCGTGGGTCGATGTTTGATGTTATGGAGCAGCAACGATGTTACGCAGCAGGGCAGTCGCCCTA
AAACAAAGTTAAACATTATGAGGGAAGCGGTGATCGCCGAAGTATCGACTCAACTATCAGAGGT
AGTTGGCGCCATCGAGCGCCATCTCGAACCGACGTTGCTGGCCGTACATTTGTACGGCTCCGCA
GTGGATGGCGGCCTGAAGCCACACAGTGATATTGATTTGCTGGTTACGGTGACCGTAAGGCTTG
ATGAAACAACGCGGCGAGCTTTGATCAACGACCTTTTGGAAACTTCGGCTTCCCCTGGAGAGAG
CGAGATTCTCCGCGCTGTAGAAGTCACCATTGTTGTGCACGACGACATCATTCCGTGGCGTTAT
CCAGCTAAGCGCGAACTGCAATTTGGAGAATGGCAGCGCAATGACATTCTTGCAGGTATCTTCG
AGCCAGCCACGATCGACATTGATCTGGCTATCTTGCTGACAAAAGCAAGAGAACATAGCGTTGC
CTTGGTAGGTCCAGCGGCGGAGGAACTCTTTGATCCGGTTCCTGAACAGGATCTATTTGAGGCG
CTAAATGAAACCTTAACGCTATGGAACTCGCCGCCCGACTGGGCTGGCGATGAGCGAAATGTAG
TGCTTACGTTGTCCCGCATTTGGTACAGCGCAGTAACCGGCAAAATCGCGCCGAAGGATGTCGC
TGCCGGCTGGGCAATGGAGCGCCTGCCGGCCCAGTATCAGCCCGTCATACTTGAAGCTAGACAG
GCTTATCTTGGACAAGAAGAAGATCGCTTGGCCTCGCGCGCAGATCAGTTGGAAGAATTTGTCC
ACTACGTGAAAGGCGAGATCACCAAGGTAGTCGGCAAATAACCCTCGAGCATTCAAGGCGCCTT
GATTATTTGACGTGGTTTGATGGCCTCCACGCACGTTGTGATATGTAGATGATAATCATTATCA
CTTTACGGGTCCTTTCCGGTGATCCGACAGGTTACGGGGCGGCGACCTCGCGGGTTTTCGCTAT
TTATGAAAATTTTCCGGTTTAAGGCGTTTCCGTTCTTCTTCGTCATAACTTAATGTTTTTATTT
AAAATACCTCGCGAGTGGCAACACTGAAAATACCCATGGAGCGGCGTAACCGTCGCACAGGAAG
GACAGAGAAAGCGCGGATCTGGGAAGTGACGGACAGAACGGTCAGGACCTGGATTGGGGAGGCG
GTTGCCGCCGCTGCTGCTGACGGTGTGACGTTCTCTGTTCCGGTCACACCACATACGTTCCGCC
ATTCCTATGCGATGCACATGCTGTATGCCGGTATACCGCTGAAAGTTCTGCAAAGCCTGATGGG
ACATAAGTCCATCAGTTCAACGGAGGTCTACACGAAGGTTTTTGCGCTGGATGTGGCTGCCCGG
CACCGGGTGCAGTTTGCGATGCCGGAGTCTGATGCGGTTGCGATGCTGAAACAATTATCCTGAG
AATAAATGCCTTGGCCTTTATATGGAAATGTGGAACTGAGTGGATATGCTGTTTTTGTCTGTTA
AACAGAGAAGCTGGCTGTTATCCACTGAGAAGCGAACGAAACAGTCGGGAAAATCTCCCATTAT
CGTAGAGATCCGCATTATTAATCTCAGGAGCCTGTGTAGCGTTTATAGGAAGTAGTGTTCTGTC
ATGATGCCTGCAAGCGGTAACGAAAACGATTTGAATATGCCTTCAGGAACAATAGAAATCTTCG

TGCGGTGTTACGTTGAAGTGGAGCGGATTATGTCAGCAATGGACAGAACAACCTAATGAACACA
GAACCATGATGTGGTCTGTCCTTTTACAGCCAGTAGTGCTCGCCGCAGTCGAGCGACAGGGCGA
AGCCCTCGAGTGAGCGAGGAAGCACCAGGGAACAGCACTTATATATTCTGCTTACACACGATGC
CTGAAAAAACTTCCCTTGGGGTTATCCACTTATCCACGGGGATATTTTTATAATTATTTTTTTT
ATAGTTTTTAGATCTTCTTTTTTAGAGCGCCTTGTAGGCCTTTATCCATGCTGGTTCTAGAGAA
GGTGTTGTGACAAATTGCCCTTTCAGTGTGACAAATCACCCTCAAATGACAGTCCTGTCTGTGA
CAAATTGCCCTTAACCCTGTGACAAATTGCCCTCAGAAGAAGCTGTTTTTTCACAAAGTTATCC
CTGCTTATTGACTCTTTTTTATTTAGTGTGACAATCTAAAAACTTGGCACACTTCACATGGATC
TGTCATGGCGGAAACAGCGGTTATCAATCACAAGAAACGTAAAAATAGCCCGCGAATCGTCCAG
TCAAACGACCTCACTGAGGCGGCATATAGTCTCTCCCGGGATCAAAAACGTATGCTGTATCTGT
TCGTTGACCAGATCAGAAAATCTGATGGCACCCTACAGGAACATGACGGTATCTGCGAGATCCA
TGTTGCTAAATATGCTGAAATATTCGGATTGACCTCTGCGGAAGCCAGTAAGGATATACGGCAG
GCATTGAAGAGTTTCGCGGGGAAGGAAGTGGTTTTTTATCGCCCTGAAGAGGATGCCGGCGATG
AAAAAGGCTATGAATCTTTTCCTTGGTTTATCAAACGTGCGCACAGTCCATCCAGAGGGCTTTA
CAGTGTACATATCAACCCATATCTCATTCCCTTCTTTATCGGGTTACAGAACCGGTTTACGCAG
TTTCGGCTTAGTGAAACAAAAGAAATCACCAATCCGTATGCCATGCGTTTATACGAATCCCTGT
GTCAGTATCGTAAGCCGGATGGCTCAGGCATCGTCTCTCTGAAAATCGACTGGATCATAGAGCG
TTACCAGCTGCCTCAAAGTTACCAGCGTATGCCTGACTTCCGCCGCCGCTTCCTGCAGGTCTGT
GTTAATGAGATCAACAGCAGAACTCCAATGCGCCTCTCATACATTGAGAAAAAGAAAGGCCGCC
AGACGACTCATATCGTATTTTCCTTCCGCGATATCACTTCCATGACGACAGGATAGTCTGAGGG
TTATCTGTCACAGATTTGGGGGTGGTTCGTCACATTTGTTCTGACCTACTGAGGGTAATTTGTC
ACAGTTTTGCTGTTTCCTTCAGCCTGCATGGATTTTCTCATACTTTTTGAACTGTAATTTTTAA
GGAAGCCAAATTTGAGGGCAGTTTGTCACAGTTGATTTCCTTCTCTTTCCCTTCGTCATGTGAC
CTGATATCGGGGGTTAGTTTGTCATCATTGATGAGGGTTGATTATCACAGTTTATTACTCTGAA
TTGGCTATCCGCGTGTGTACCTCTACCTGGAGTTTTTCCCACGGTGGATATTTCTTCTTGCGCT
GAGCGTAAGAGCTATCTGACAGAACAGTTCTTCTTTGCTTCCTCGCCAGTTCGCTCGCTATGCT
CGGTTACACGGCTGCGGCGAGCGCTAGTGATAATAAGTGACTGAGGTATGTGCTCTTCTTATCT
CCTTTTGTAGTGTTGCTCTTATTTTAAACAACTTTGCGGTTTTTTGATGACTTTGCGATTTTGT
TGTTGCTTTGCAGTAAATTGCAAGATTTAATAAAAAAACGCAAAGCAATGATTAAAGGATGTTC
AGAATGAAACTCATGGAAACACTTAACCAGTGCATAAACGCTGGTCATGAAATGACGAAGGCTA
TCGCCATTGCACAGTTTAATGATGACAGCCCGGAGGCGAGGAAAATAACCCGGCGCTGGAGAAT
AGGTGAAGCAGCGGATTTAGTTGGGGTTTCTTCTCAGGCTATCAGAGATGCCGAGAAAGCAGGG
CGACTACCGCACCCGGATATGGAAATTCGAGGACGGGTTGAGCAACGTGTTGGTTATACAATTG
AACAAATTAATCATATGCGTGATGTGTTTGGTACGCGATTGCGACGTGCTGAAGACGTATTTCC
ACCGGTGATCGGGGTTGCTGCCCATAAAGGTGGCGTTTACAAAACCTCAGTTTCTGTTCATCTT
GCTCAGGATCTGGCTCTGAAGGGGCTACGTGTTTTGCTCGTGGAAGGTAACGACCCCCAGGGAA
CAGCCTCAATGTATCACGGATGGGTACCAGATCTTCATATTCATGCAGAAGACACTCTCCTGCC
TTTCTATCTTGGGGAAAAGGACGATGTCACTTATGCAATAAAGCCCACTTGCTGGCCGGGGCTT
GACATTATTCCTTCCTGTCTGGCTCTGCACCGTATTGAAACTGAGTTAATGGGCAAATTTGATG
AAGGTAAACTGCCCACCGATCCACACCTGATGCTCCGACTGGCCATTGAAACTGTTGCTCATGA
CTATGATGTCATAGTTATTGACAGCGCGCCTAACCTGGGTATCGGCACGATTAATGTCGTATGT
GCTGCTGATGTGCTGATTGTTCCCACGCCTGCTGAGTTGTTTGACTACACCTCCGCACTGCAGT
TTTTCGATATGCTTCGTGATCTGCTCAAGAACGTTGATCTTAAAGGGTTCGAGCCTGATGTACG
TATTTTGCTTACCAAATACAGCAATAGTAATGGCTCTCAGTCCCCGTGGATGGAGGAGCAAATT
CGGGATGCCTGGGGAAGCATGGTTCTAAAAAATGTTGTACGTGAAACGGATGAAGTTGGTAAAG
GTCAGATCCGGATGAGAACTGTTTTTGAACAGGCCATTGATCAACGCTCCTCAACTGGTGCCTG
GAGAAATGCTCTTTCTATTTGGGAACCTGTCTGCAATGAAATTTTCGATCGCCTGATTAAACCA

CGCTGGGAGATTAGATAATGAAGCGTGCGCCTGTTATTCCAAAACATACGCTCAATACTCAACC
GGTTGAAGATACTTCGTTATCGACACCAGCTGCCCCGATGGTGGATTCGTTAATTGCGCGCGTA
GGAGTAATGGCTCGCGGTAATGCCATTACTTTGCCTGTATGTGGTCGGGATGTGAAGTTTACTC
TTGAAGTGCTCCGGGGTGATAGTGTTGAGAAGACCTCTCGGGTATGGCCAGGTAATGAACGTGA
CCAGGAGCTGCTTACTGAGGACGCACTGGATGATCTCATCCCTTCTTTTCTACTGACTGGTCAA
CAGACACCGGCGTTCGGTCGAAGAGTATCTGGTGTCATAGAAATTGCCGATGGGAGTCGCCGTC
GTAAAGCTGCTGCACTTACCGAAAGTGATTATCGTGTTCTGGTTGGCGAGCTGGATGATGAGCA
GATGGCTGCATTATCCAGATTGGGTAACGATTATCGCCCAACAAGTGCTTATGAACGTGGTCAG
CGTTATGCAAGCCGATTGCAGAATGAATTTGCTGGAAATATTTCTGCGCTGGCTGATGCGGAAA
ATATTTCACGTAAGATTATTACCCGCTGTATCAACACCGCCAAATTGCCTAAATCAGTTGTTGC
TCTTTTTTCTCACCCCGGTGAACTATCTGCCCGGTCAGGTGATGCACTTCAAAAAGCCTTTACA
GATAAAGAGGAATTACTTAAGCAGCAGGCATCTAACCTTCATGAGCAGAAAAAAGCTGGGGTGA
TATTTGAAGCTGAAGAAGTTATCACTCTTTTAACTTCTGTGCTTAAAACGTCATCTGCATCAAG
AACTAGTTTAAGCTCACGACATCAGTTTGCTCCTGGAGCGACAGTATTGTATAAGGGCGATAAA
ATGGTGCTTAACCTGGACAGGTCTCGTGTTCCAACTGAGTGTATAGAGAAAATTGAGGCCATTC
TTAAGGAACTTGAAAAGCCAGCACCCTGATGCGACCACGTTTTAGTCTACGTTTATCTGTCTTT
ACTTAATGTCCTTTGCTACAGGCCAGAAAGCATAACTGGCCTGAATATTCTCTCTGGGCCCACT
GTTCCACTTGTATCGTCGGACTGATAATCAGACTGGGACCACGGTCCCACTCGTATCGTCGGTC
TGATTATTAGTCTGGGACCACGGTCCCACTCGTATCGTCGGTCTGATTATTAGTCTGGGACCAC
GGTCCCACTCGTATCGTCGGTCTGATAATCAGACTGGGACCACGGTCCCACTCGTATCGTCGGT
CTGATTATTAGTCTGGGACCATGGTCCCACTCGTATCGTCGGTCTGATTATTAGTCTGGGACCA
CGGTCCCACTCGTATCGTCGGTCTGATTATTAGTCTGGAACCACGGTCCCACTCGTATCGTCAG
TCTGATTATTAGTCTGGGACCACGGTCCCACTCGTATCGTCGGTCTGATTATTAGTCTGGGACC
ACGATCCCACTCGTGTTGTCGGTCTGATTATCGGTCTGGGACCACGGTCCCACTTGTATTGTCG
ATCAGACTATCAGCGTGAGACTACGATTCCATCAATGCCTGTCAAGGGCAAGTATTGACATGTC
GTCGTAACCTGTAGAACGGAGTAACCTCGGTGTGCGGTTGTATGCCTGCTGTGGATTGCTGCTG
TGTCCTGCTTATCCACAACATTTTGCGCACGGTTATGTGGACAAAATACCTGGTTACCCAGGCC
GTGCCGGCACGTTAACCGGGCTGCATCCGATGCAAGTGTGTCGCTGTCGACGGCCTCCTCACCC
GGTCACGTGAGCTCATTTAACCCACTCCACAAAAAGGCTCAACAGGTTGGTGGTTCTCACCACC
AAAAGCACCACACCCCACGCAAAAACAAGTTTTTGCTGATTTTTCTTTATAAATAGAGTGTTAT
GAAAAATTAGTTTCTCTTACTCTCTTTATGATATTTAAAAAAGCGGTGTCGGCGCGGCTACAAC
AACGCGCCGACACCGTTTTGTAGGGGTGGTACTGACTATTTTTATAAAAAACATTATTTTATAT
TAGGGGTGCTGCTAGCGGCGCGGTGTGTTTTTTTATAGGATACCGCTAGGGGCGCTGCTAGCGG
TGCGTCCCTGTTTGCATTATGAATTAGTTACGCTAGGGATAACAGGGTAATATAGAACCCGAAC
GACCGAGCGCAGCGGCGGCCGCGCTGATACCGCCGCGCCCGCTCTAGATAATACGACTCACTAT
AGGAAAAAAACTTTATATAACTCTTATATAAGTGCCCTTAGCGGGGCTCCCCGGCTACGGTCGG
ATCCCTACGGGGAGGATAGGGTGAAAACCCCTAGTGCAAGCTGACACTCATACCTCCCAAGGTC
CATGAGTCGACGCAAAGGTCCTCGAAAGCATGTTGTCCTTTCGTACAACCGAGTAGGTTCGTTG
CCTTAATTGGTGACGCTTGCAGGATGAGGATGGTCCCGACGCCTAACGGACCTTGCTGCCTTCT
TTCCCTGGATTGGCGGTGTTGTTCCCACTAATAATAAAGGAATACGCACATGTTGCTGCCTGTA
GTAGCCCGTGCGGCCGTCCCTGCTATTGAGAGTGCCATTGCGGCTACTCCTGGCCTGGTTTCCC
GAATCGCAGCCGCGATCGGTTCCAAGGTCAGCCCTTCCGCCATTTTGGCGGCGGTCAAGAGCAA
CCCGGTCGTCGCAGGTCTGACACTCGCTCAGATCGGAAGCACCGGTTATGACGCCTATCAGCAG
CTTCTGGAGAATCATCCAGAGGTCGCCGAGATGCTGAAAGACCTGTCTTTCAAAGCCGACGAAA
TCCAGCCGGATTTCATCGGTAACCTCGGTCAGTACCGCGAAGAGCTGGAACTGGTCGAAGATGC
TGCCCGCTTCGTGGGCGGCATGTCGAACCTGATTCGCCTGCGCCAGGCCCTGGAGCTTGATATC
AAGTACTACGGCCTGAAAATGCAGCTGAATGACATGGGATACCGCTCGTAATGGTTATCGGTCT

TCTGAAGTATCTCACGCCTGCCGTTAAGGTGCAGATGGCTGCTCGCGCGTTGGGCCTGTCCCCC
GCCGAAGTCGCTGCAATTGACGGCACGTTGGGTCGTGTCTCTGCGATGCCAGCGGTCGCGGTCG
TGCTGGGAGGGAAACCTCTCTCTCTGGCCACGATCGCGTCAGTTGTGTCTGATGCAAACCCCAG
TGCCACTGTTGGCGCGCTTATGCCTGCTGTACAGGGCATGGTGAGTTCCGACGAAGGCGCGAGT
GCGTTGGCTAAGACCGTGGTAGGCTTCATGGAGTCCGACCCCAACAGCGATGTCCTGGTTCAAC
TGCTCCACAAGGTGTCAAACTTGCCGATTGTCGGCTTTGGTGACACGCAGTATGCAGACCCAGC
TGACTTCTTGGCCAAGGGAGTTTTCCCTCTGATCAGGAAGCCAGAAGTAGAGGTTCAAGCTGCG
CCTTTCACCTGTCGTCAGTGTGATCATGTTGATCACATCACTGATGTACCTCAAACTTCGACCT
TTGTTCACAAATGCACTTCGTGCGGCTTTGTGCAGATGGTCCACCGTAAGGATGTTCCGTAATG
CCATTTCCTCTGGTAAAGCAAGACCCAACCTCGAAGGCTTTCACTGAAGCCAGTGAACGCTCCA
CCGGCACCCAGATCCTGGACGTCGTCAAGGCCCCTATCGGCCTGTTCGGCGACGATGCCAAACA
CGAGTTCGTGACCCGTCAGGAACAAGCCGTTTCCGTCGTCAGCTGGGCAGTTGCTGCCGGTCTG
ATCGGCGAGCTGATCGGCTACCGTGGTGCGCGTTCGGGTCGCAAAGCGATCCTGGCCAACATCC
CTTTTCTGGCCTAACTCCTCGTGTCCAAGGATAGCGCCTTCGCAGTGCAATACTCGCTGCGCGC
CCTGGGACAAAAGGTGCGGGCAGACGGGGTAGTGGGCTCTGAGACTCGTGCCGCGCTGGATGCG
CTGCCCGAGAATCAGAAGAAAGCGATTGTAGAGTTGCAAGCACTCCTACCGAAAGCACAGTCGG
TCGGCAACAACCGTGTGAGGTTCACAACAGCTGAAGTCGACTCGGCGGTGGCGCGGATCTCGCA
AAAGATAGGTGTTCCGGCTTCCTACTACCAGTTCCTGATTCCGATCGAGAACTTCGTGGTGGCC
GGTGGTTTCGAAACCACCGTTTCTGGTTCCTTCCGTGGGTTGGGCCAGTTCAACCGGCAGACGT
GGGATAGACTCCGTCGTTTAGGCCGTAACCTTCCTGCATTTGAGGAGGGTTCGGCACAACTGAA
CGCTTCTCTTTATGCAATCGGGTTCTTGTATCTTGAGAACAAGAGAGCGTACGAGGCGTCGTTC
AAAGGCCGCGTTTTCACTCACGAAATCGCGTATTTGTATCACAACCAAGGCGCTCCAGCTGCCG
AACAGTACCTGACTTCGGGTCGGCTCGTTTACCCGAAGCAAAGCGAGGCCGCTGTCGCGGCGGT
TGCGGCTGCGAGAAACCAGCATGTCAAAGAGAGTTGGGCTTAGCCCTGAACTGCATCGTGAACT
GAAAATGTTCCCAGATGTCACGAAGGGTGGCACGTTCGACATAACCATCCGGTCGACTACCGAG
AACGGTGCTTTTTGGGCGAACTACGAAGGTAGAACGTCCTTGGTCACCGTCCCGGACGTGAAGA
CAGCTATCGAGTTTTTGATTAAACTCTGCCGTCGACACAAGTTGTCCAATCAGGTGAACACGCG
AACGCTTCTCCGCGATTTGCAACGAACGTTGCAGGAATGTGAATGCCAGTCTCATCATGTGCCG
TTGTCCAGCCCCTTCATGCATCTCAGATTTGCGTAAAGCTGATCGGAAGCTATGAAAGTAAGCT
GAGCGACACGGAAGTTATTGAAGCAGCTATCAAGCTCTCATAGGCTTGGAAAGCCCGGCATCGA
TGTCGTTCCATGTCGCGCCAGACGCGGCCACCGATATGTATCTTGATCTGATCGAAATCTACTC
CCCGTCGTCAGTCGGGATACATCTCGTCCTGCCATAAGCGCTGTCTGTAGCGTGCATAAACAGA
TAGATCGCCTTTTTAGGTAACCGCGGATTGATCACCGTTCCGAGCTTGCTTGGATAAACAAGTC
CTTGTATAACAAGGCGAGACTCACTATGTGAGCGTCCAATAGGACGGCCCCTTCGGGGGCTCTC
TCTCTCCGTACTAGCATAACCCCTTGGGGCCTCTAAACGGGTCTTGAGGGGTTTTTTGGGTACC
CCCGGTAAACCAGCAATAGACATAAGCGGCTATTTAACGACCCTGCCCTGAACCGACGACCGGG
TCATCGTGGCCGGATCTTGCGGCCCCTCGGCTTGAACGAATTGTTAGACACTCAGTAATACGAC
TCACTATAGGAAAAAAACTTTATATATTTTCTACGTTGAGCTCCGTATAAAGCTCCGTGCCCGC
ACACGCCCGCTACGGCGGTATTGTCTAACCGGCGACAATAAACAGCTGCTGCTTACAAGCTTAC
AGTTGACCGGAGTCTCGGCGTGCAGCGCCTAAACACGGGAAACCGTGGTGGTGACACCCTCTGC
TGAGGGCTTATAGTGGTGATATTCCTCCCCAGGAGTTCCCTCCCATTTCGGCCACTGCGCTCTA
ACCATGAGCGCGGTCTATTTGAGAGTGTCGCTCTTCTGCCTACGCGCTCATTCGTTCCCTCGAG
TTGACGCTTCAAGCAGGTGGACACCTCCTCAACCCATAATAAGAGATCCATTCAATGGACAACA
TCCTCGATCCCCTTAAGGCTCCGTTTTCTTCGGAAGCCGCCGCGAAAACCACCGCTGCCAAAAT
CGCTGTGGTATACGCGTTGGTCGGTCTGGTTGGCGGTCTGCTGCTAACCAAGTAAGGTGTAGTA
TGCATGACACGCGACCGCTCCGAACCGAAAGACCCATGGCCAGCAAGAATACGAATGACCGGGT
TTTCGACCGGTTTCATTCTTCTCTCGTTCGGCTGTGGCATTCTGCAAACCAGCGCATGCGCGGT

TCTTTCTCCGTCGTGGTTCGGGTCGAGCACTCTTTAGTGCTCCTCATCGGCTACACGGTGGTAG
GCGCGACTGTCGCACACTTCGTGAGGTGACTATGTTAGCTTTCGTAGCGCGAGCGGTCGTACTT
TACTCTGCTGGTGTAGTCGTGGGCATCGCCTACGATCACGTCACAGGAAGGAAACGTCGCCATG
ACTAAGTGGAAGATGTACATCGCCGGCGTCGTTCTGGTCATCGTAGGGGCAGTTACTCATGCTC
CACAGCTGATGGTCCAGGGCATGACTACGCTCGCGACTCAAGCGGCCGCAGACGCGGCCGATGG
TGGAGGTGCTCAGTGAGTATCTTCTCCTCGTTGTTCAAGGTCATCAAGAAGGTAATCTCGAAGG
TGGTCGCCACCCTTAAGAAAATCTTCAAGAAGATCTGGCCGTTGCTACTTATTGTGGCAATTAT
CTACTTCGCTCCCTACCTCGCCGGGTTCTTCACTTCCGCCGGGTTCACTGGGATCGGAGGGATC
TTCTCCTCTATCGCAACCACCATCACGCCTACGCTGACGTCGTTCCTGTCGACTGCGTGGTCTG
GTGTGGGCTCTCTTGCCTCCACGGCTTGGTCTGGGTTCCAATCTCTCGGGATGGGTACTCAGCT
CGCTGTCGTGAGTGGCGCGGCTGCTCTGATTGCACCTGAGGAAACGGCTCAACTGGTTACCGAA
ATCGGTACCACCGTAGGTGATATCGCCGGTACGATTATCGGCGGTGTCGCCAAGGCACTCCCGG
GTTGGATCTGGATCGCCGCAGGCGGTCTTGCCGTCTGGGCCCTCTGGCCGTCATCTGACAGTAA
GGAGTAGCAAATGCGCTACCAAGGCATCAACGAGTGGCTGGGTGGAGCCAAGAAACTCACCACC
GCAAACGGTGAGATTGGCGCTATCTACCTCTCCGCTGCTCCTCCCACCGACGCCGCACGTGCGG
ACGCTAAGGCGGTGGATTTTACTGCTGGTTGGCCAAGCGCGATCGTTGACTGCGCTGATGCCAC
TCGTGCCAAGCAGAACTACCTGTGGGTTGGCGATAACGTTGTGCACATCGGGGCTAAACACGTT
CCACTCCTCGATCTGTGGGGCGGGACAGGTGATGCCTGGCAGCAGTTCGTTGGCTATGCCTGCC
CAATGCTCGACCTTTGTCGTGCGTGGGGCCTGGGTTATGCCAGCGCTTCTGTAACCACCGGCTC
GTTGCAGGGCTATCAGCCATCGGCGTTCTTGGACGTTGAGCAACAGCAGTTCGCGAAGGACAAT
CTCAACCTGTATGGCGATAACTGCCTTGACCTGGCCACCAGTTCGTCCGCTCAGCGGGCATTTC
TGGAGCAGTGCATGGGCTGCGCCTTGCCGGAGGATTGCATCTTCGGTTGGTATGTGAAAATGGA
TTGGGAAGGTTCGGCAGTTGCCGACGCCTACGCTGCGATCCGTGTCCAAGGGTTCGCCACTGTA
ATGGCACCTTGGCAGTCGGTTGGCGGTGCTGGCTACGTTTACGCTCGTGTGCCTCAAAAAGGCG
CGTGGATGGGTGTGAACCTGCTTGCCTATGTCCACGGCACCAGTGGCCAGCCTGCTTATGGCAT
TCCGATGACCCTCTCGGGGTTCACCGGTAACATGGGTCAGGTGGCTTCGAAGTGGCTCATGCTT
CCTCTCCTGATGATCGTCGACCCTCATGTCGTCCAGATTTTGGCCGCACTGGGGGTTAAACGTG
GGACCAAATCGGACCCACGGACGACCGACGTGTACGCTGATCCGAAGGTTCCGGCTAGCCGTAT
TTCCGGGCCGATGATCAATGGAACGGTTGCTCCTCCTGCGACGATCCCCGCTACCATTCCGGTG
CCTCTGGCGCCGCTCGGTGGCGCGGGTGGCCCTGGCGCTCAGGGTTTCCAGGTATACCCCGTTT
TCACCTGGGGTCTGCCTGAGTTCATGACCGACGTGACCATCGAAGGTACCGTCACTGCGGACTC
CAACGGTCTGCATGTCGTGGACGACGTGCGTAACTACGTCTGGAACGGTACTGCTCTTGCTGCA
ATTGAGCAGGTCAATGCCGCTGACGGTCGAGTTACGCTCACTGACTCTGAGCGTGCTCAACTCG
CCTCGTTGACTGTTCGAACCGCATCGTTGCGTCAGCAGCTGTCGGTTGGGGCAGACCCCTTGTC
CAAGACGTCGATCTGGCGTCGGGCTCAAAAGGCCGATTATGATCTGCTGTCTCAACAGATCATC
GAAGCGGACACGGTGAAAAACCTACCTGCTGTGACGTTCGCTCAGGCGAACAAAGCGGCAGGCG
GTCAATCCGAGACATTGTGGCACCAGATGTATCGGGTCAACGATATCGCTGGCGATCAAGTCAC
CGCAATCCAAATCACTGGTACGATGGCGACTGGCATTCGATGGTCGGCAACTGCTGGCGGTCTG
GTCGTCGATGCTGACGAGCAAGATGCGGTGATCGCGATTTCGTCCGGTAAGCCGGTCAAGAACA
GCTCCGACCTTCCTACGGCCGACGCTGTGAACTACTTGTTCGGTATCACTGCGGACGATATGCC
TGGTATCGTTTCCTCGCAAAAGGAAATGAACAGCGAGTTTGAAGAAGGTTTCCTTCAGAAAGCT
CGTCTTTGGAACCCACGTAAGCTCGTCGAAAACGTCCAGAATGCCTATTTCCTGATGGTGTACG
CTCGCGATCGGAAGCAATTCCACTCGTTGGTGGCATCCTCTCTGGCGATGGCCAAGCTGGGCGT
AAGTACGCGGGCCTGTAAGGAGTCGTATGGCTGCTGAACAATCCTCCGGTATGAGCGCGTTCAC
CAAAGGCACGATCGTGATCTGCCTGGTGGTGGTCGTCCTCAATCTCATCGGGAAGTGACCATGG
TACCGCTAAAAATTAGCACGCTGGAGTCCCAGCTGCAACCGCTTGTTAAGTTGGTTGCAACCGA
AACCCCCGGTGCCCTCGTAGCGTATGCTCGAGGGTTATCGAGTGCCGACCGCTCGCGGTTGTAC

AGACTGCTTCGTTCTTTGGAGCAGGCCATCCCGAAGCTGTCGTCGGCTGTCGTTTCGGCCACGA
CGTTGGCAGCGCGAGGTCTTTAATGGAAACCAACCCGCTGCTTCAGCTTGAGTCGCTGTCGTTA
CGCTTGCGAGACATGCCTCGTTCGCGCCTTTCTGCGCTGATGAAGAACATGTCGTATGAGCAGC
TGCAGTCGTTGTATAGCACCAGCGTAAAAGTTGGCGCTGTGCTCGATAGCGTTTCAATGCAGTT
GCTTGAGGCGTCACAAACCGCTCAATCGGGAACTCGACTGATGACACCGCAGGAGTACGTCGCT
GCTGGTGGAGGTCGTGTGTACGTTAAATAAGTCCTTAGATTTCTAAGGCGAGACTCGCTTTGCG
AGCATCCAATAGGATGGCCCCTTCGGGGGCTCTCTCTCTCTAGCATAACCCCTTGGGGCCTCTA
AACGGGTCTTGAGGGGTTTTTTGGGATCCATTCACCACCCTGAATTGACTGGACATCCGGTAAT
ACGACTCACTATAGTAAAAAAACTTTATATAGTCTTTTACCTGGATTCTCTGTGCAGAACTGAG
AACTGAACGCTACCCTTGCGGGGGATGCGGCCCCGGTCTACGGCCTAGGGATCCAGCGTGGCTC
ACGGGCCGCCGGAACTGACGTCCGTAACAAACGTCCTTGGGATAGGAGTACAGTAACCACTCTT
AGATACCCGATTCCCCTGTTTCTGCGTGGAAGCCTTTCGACAGCTACCCAGCTTAGATCGTCTG
GTGCCCTAAATCCCTGGAGATAACCAATGGCTACATTACAAGATGTGCATCTACGGGTGAATGA
CCGGGTAACACCGGTGTACTTCACTGCTCGCTCGTTTCTGCTCGTTTCTCCGAAACGTGCGGGG
CAAGCAACGTTCCTCGCTCGCGAGGAGGGTACTGACAATCCTGTCGTTACCTGTCATGTATCCG
ACTTTTATAAGGACGGTGTGTAATGACTTTGTACCTGGTCCCTCCGCTGGATTCGGCGGACAAA
GAGTTGCCTGCTCTGGCTTCCAAAGCTGGGGTAACGCTTCTCGAGATCGAGTTTCTTCACGAGC
TCTGGCCTCACCTCAGTGGTGGTCAGATCGTGATCGCCGCTCTCAACGCCAACAATCTGGCCAT
CCTCAACCGTCACATGTCCACTCTGTTGGTCGAGTTGCCGGTTGCTGTGATGGCCGTTCCCGGT
GCTAGCTATCGTTCCGATTGGAACATGATCGCTCACGCACTCCCGTCTGAGGATTGGATCACTT
TGTCCAACAAGATGCTGAAAAGCGGCTTGCTGGCGAACGATACCGTCCAGGGCGAGAAGCGCTC
CGGCGCTGAGCCGCTGTCGCCGAACGTGTACACCGATGCGCTCTCGCGTCTCGGTATCGCGACG
GCCCATGCTATCCCCGTTGAACCCGAACAACCGTTCGATGTCGATGAGGTAAGCGCCTGATGCC
GAGGAGAGCTCCCGCGTTCCCTCTGAGCGATATCAAGGCTCAGATGCTGTTCGCAAATAACATC
AAGGCCCAACAAGCCTCGAAGCGTAGCTTCAAAGAGGGGGCGATTGAAACGTACGAAGGGCTGC
TTTCAGTAGACCCTCGGTTTTTGAGTTTCAAGAACGAGCTCTCTCGGTATCTGACCGACCACTT
CCCGGCGAACGTCGACGAGTATGGTCGTGTTTATGGAAACGGTGTTCGTACCAACTTCTTTGGT
ATGCGCCACATGAACGGGTTTCCAATGATCCCCGCGACGTGGCCACTCGCTTCCAACCTTAAGA
AACGTGCCGACGCTGACCTAGCCGATGGCCCTGTTTCTGAGCGCGACAATCTACTCTTTCGCGC
CGCAGTCCGGCTTATGTTTTCAGATCTAGAGCCTGTTCCGCTGAAGATCCGTAAAGGATCGTCA
ACCTGCATCCCGTATTTTTCTAACGATATGGGAACGAAGATCGAGATCGCCGAGCGCGCTCTTG
AGAAAGCGGAAGAAGCTGGCAATCTGATGCTGCAAGGTAAGTTTGATGACGCCTACCAGCTCCA
CCAAATGGGTGGTGCCTATTACGTCGTGTATCGTGCACAATCGACCGATGCTATCACACTCGAC
CCTAAGACCGGAAAATTCGTGTCAAAGGATCGTATGGTCGCTGACTTCGAATACGCAGTCACGG
GCGGTGAGCAAGGCTCGCTGTTCGCTGCTTCGAAGGATGCCTCTCGTTTGAAGGAACAGTACGG
GATAGATGTCCCGGACGGGTTTTTCTGCGAGCGGCGTCGTACCGCTATGGGTGGTCCGTTCGCG
TTGAACGCTCCTATCATGGCCGTTGCGCAACCTGTGCGAAACAAAATTTACTCCAAGTACGCTT
ACACCTTTCACCATACTACTCGTCTTAATAAGGAGGAAAAGGTGAAAGAGTGGTCGTTGTGCGT
CGCTACTGACGTATCCGACCACGACACGTTCTGGCCTGGATGGCTGCGGGATCTCATCTGTGAT
GAACTGCTCAACATGGGGTACGCTCCGTGGTGGGTTAAGTTGTTCGAAACCTCGCTCAAACTGC
CCGTTTACGTGGGCGCTCCTGCTCCTGAGCAGGGCCACACGTTGTTGGGTGATCCGTCCAACCC
TGATCTCGAAGTTGGGCTCTCGTCCGGACAAGGGGCGACCGACCTCATGGGCACGTTGCTCATG
AGTATCACCTACCTGGTGATGCAACTTGATCACACCGCTCCTCACCTCAACAGTCGAATCAAGG
ACATGCCATCAGCATGCCGCTTTCTTGACTCGTATTGGCAAGGACACGAGGAGATCCGTCAGAT
CTCAAAATCTGATGATGCTATACTTGGCTGGACCAAAGGTCGTGCTTTGGTTGGTGGTCATCGT
TTGTTCGAGATGCTGAAAGAGGGTAAGGTTAACCCCTCACCTTACATGAAGATCTCCTACGAGC
ACGGTGGCGCCTTCCTTGGTGACATCCTGCTTTACGACTCGCGTCGTGAGCCTGGCTCTGCCAT

CTTCGTTGGTAACATCAACTCAATGCTGAACAACCAGTTCAGCCCTGAGTACGGTGTCCAATCG
GGCGTTCGCGACCGATCTAAGCGCAAACGGCCGTTCCCCGGTCTTGCTTGGGCGTCGATGAAAG
ATACCTACGGTGCCTGTCCGATCTACTCTGATGTGCTGGAGGCGATCGAGCGTTGCTGGTGGAA
CGCGTTCGGTGAGTCGTACCGTGCGTATCGTGAAGATATGCTTAAACGCGACACTCTCGAACTA
TCACGCTACGTTGCGTCGATGGCTCGTCAAGCCGGGCTGGCTGAACTCACTCCCATTGATTTGG
AGGTGCTTGCTGACCCGAACAAACTCCAGTATAAGTGGACCGAGGCCGATGTCTCGGCGAATAT
CCACGAGGTACTGATGCATGGCGTATCGGTCGAAAAGACTGAGCGCTTTCTCCGTTCTGTAATG
CCGAGGTAATCATGCCGATTGTCGTAACTCAAGCGCATATTGATCGTGTCGGCATCGCCGCCGA
TCTGCTCGATGCGTCTCCTGTGTCGCTTCAAGTTCTTGGTCGCCCTACCGCGATCAACACTGTC
GTCATCAAGACGTACATCGCTGCTGTTATGGAGCTCGCCTCCAAGCAAGGTGGTTCGTTGGCCG
GTGTGGATATTCGTCCTTCGGTTCTGCTGAAAGACACCGCTATCTTCACCAAGCCGAAGGCGAA
GTCCGCTGACGTCGAATCTGATGTCGACGTTCTGGACACGGGGATTTACTCCGTTCCTGGACTG
GCTCGCAAGCCTGTCACCCACCGTTGGCCATCAGAGGGTATCTACTCTGGTGTCACAGCTCTGA
TGGGCGCTACCGGTTCCGGTAAGTCGATCACGCTGAACGAAAAGCTCCGTCCAGACGTCCTGAT
TCGTTGGGGCGAGGTGGCTGAAGCTTACGATGAGCTGGATACCGCCGTCCACATCTCGACTCTG
GATGAGATGTTGATTGTGTGTATTGGCCTGGGTGCACTCGGGTTCAACGTCGCTGTTGACTCGG
TTCGTCCTCTGCTGTTCCGTCTCAAAGGCGCCGCCTCTGCGGGGGGTATTGTGGCTGTGTTCTA
CAGCCTGTTGACCGATATCTCGAACTTGTTCACACAATACGATTGCTCTGTCGTCATGGTCGTT
AACCCGATGGTTGACGCTGAGAAGATCGAGTACGTGTTCGGTCAGGTCATGGCTTCGACTGTCG
GTGCGATCTTGTGTGCTGATGGCAACGTGTCCAGAACGATGTTCCGGACCAACAAAGGTCGTAT
TTTCAACGGTGCGGCCCCTCTTGCTGCTGACACTCACATGCCTAGCATGGATCGTCCTACCAGC
ATGAAGGCCCTCGATCATACCTCGATCGCCTCTGTCGCACCGCTGGAGCGTGGCTCCGTGGATA
CCGACGATCGCAATTCCGCTCCGCGCCGTGGCGCTAACTTCTCTCTGTAAGGGTATAAGATGTT
CAACCTCAAAGTTAAAGATCTGAACGGTTCCGCTCGCGGTCTGACTCAAGCTTTCGCCATCGGC
GAATTGAAGAACCAGCTGTCCGTCGGCGCGTTGCAGTTGCCGTTGCAGTTCACGCGCACGTTCT
CCGCTTCCATGACCAGCGAGTTGCTTTGGGAAGTGGGCAAGGGCAACATCGACCCAGTGATGTA
CGCTCGTCTGTTTTTCCAGTACGCGCAAGCTGGCGGCGCTCTGTCCGTTGATGAGCTCGTAAAC
CAGTTCACTGAGTATCACCAATCCACGGCCTGTAACCCTGAAATCTGGCGCAAGCTGACTGCTT
ACATCACCGGTTCCTCGAACCGCGCGATCAAAGCTGACGCTGTAGGCAAGGTGCCTCCAACCGC
GATCCTGGAGCAGTTGCGCACTCTCGCTCCCTCGGAGCACGAGTTGTTTCACCACATCACGACC
GACTTCGTCTGCCATGTGCTGTCTCCCCTCGGTTTCATCCTGCCTGACGCTGCCTACGTGTACC
GCGTTGGTCGCACCGCTACGTACCCCAATTTCTACGCTCTTGTAGATTGCGTACGTGCGAGCGA
CCTGCGTCGTATGCTGACAGCGCTGTCGTCTGTCGATTCGAAGATGCTTCAAGCCACGTTCAAA
GCCAAAGGCGCTCTTGCCCCTGCTTTGATCTCCCAGCATCTGGCTAACGCCGCCACTACTGCTT
TCGAGCGGTCGCGCGGTAACTTCGATGCCAATGCTGTGGTGTCGTCCGTTCTGACCATTCTTGG
TCGTCTCTGGTCGCCTTCCACCCCGAAGGAGCTCGACCCGAGTGCGCGTTTGCGCAACACCAAC
GGTATCGATCAGCTGCGCAGTAACCTGGCGCTGTTCATCGCGTACCAGGATATGGTCAAGCAAC
GCGGTCGCGCCGAAGTCATCTTCTCTGACGAGGAGCTGTCGTCGACGATCATCCCTTGGTTCAT
CGAGGCGATGAGCGAAGTGTCCCCGTTCAAACTGCGTCCGATCAACGAGACTACCAGCTATATC
GGTCAGACCTCCGCGGTCGACCACATGGGCCAGCCGAGCCATGTTGTGGTCTACGAAGACTGGC
AGTTTGCCAAGGAGATCACCGCTTTCACTCCTGTCAAGCTGGCCAACAACTCGAATCAGCGTTT
CCTGGACGTTGAGCCTGGTATCTCTGATCGTATGTCGGCTACGCTGGCACCAATCGGCAACACG
TTCGCGGTTTCGGCGTTCGTCAAGAACCGCACCGCCGTTTACGAGGCTGTCTCGCAGCGTGGTA
CAGTCAACAGCAACGGCGCGGAGATGACCCTCGGGTTCCCTTCCGTTGTTGAACGCGACTACGC
TCTCGACCGTGACCCTATGGTCGCGATCGCTGCTCTGCGCACTGGTATCGTCGATGAAAGTCTC
GAGGCTCGCGCTTCGAACGATCTGAAACGGTCGATGTTCAACTACTACGCGGCTGTGATGCATT
ACGCTGTTGCTCACAATCCTGAAGTTGTTGTTTCGGAGCACCAAGGTGTTGCCGCCGAACAAGG

TTCGCTCTACCTGGTGTGGAACGTCCGCACTGAGCTGCGAATCCCTGTTGGTTACAACGCCATC
GAGGGCGGTTCGATCCGTACCCCTGAGCCGTTGGAGGCGATCGCCTACAACAAGCCGATCCAAC
CGTCCGAGGTGCTGCAAGCCAAGGTACTGGATTTGGCTAACCACACAACCTCGATTCACATCTG
GCCGTGGCATGAGGCTTCGACCGAGTTCGCGTACGAAGACGCCTACTCTGTCACCATCCGCAAC
AAACGCTACACCGCCGAAGTCAAGGAGTTCGAACTCCTCGGGCTCGGTCAACGTCGCGAACGTG
TACGGATCCTCAAGCCTACGGTAGCCCACGCTATCATCCAGATGTGGTATTCCTGGTTCGTCGA
GGACGACCGCACTTTGGCAGCTGCCCGTCGCACGTCTCGCGATGACGCCGAGAAGCTTGCCATC
GACGGTCGTCGTATGCAAAACGCTGTGACCTTGCTTCGCAAGATCGAGATGATTGGGACAACCG
GTATCGGTGCGTCTGCCGTCCACCTCGCGCAGTCGCGCATCGTGGATCAGATGGCCGGTCGAGG
GCTCATCGACGACAGCTCCGATCTCCATGTCGGTATCAACCGTCACCGTATCCGCATCTGGGCC
GGCCTCGCCGTTCTCCAGATGATGGGGCTCTTGAGCCGCTCCGAAGCGGAAGCTCTCACCAAGG
TCCTTGGTGATAGCAACGCTCTGGGCATGGTTGTCGCCACAACCGACATTGATCCATCCCTGTA
ACTCTCGTAAGCTCTCATAAACCTTTCGTTATAATTCCATAAGTCCTTAGATTTCTAAGGCGAG
ACTCGCTTTGCGAGCGTCCAATAGGACGGCCCCCTCGGGGGCTCTCTCTCTCTAGCATAACCCC
TTGGGGCCTCTAAACGGGTCTTGAGGGGTTTTTTGCATGGTCATAGCTGTTTCCTGTGTG

**>pJLC141 (CloDF13 ori, KanR, contains phi6 S cDNA segment under T7 transcriptional control)**

CCGTACTAGCATAACCCCTTGGGGCCTCTAAACGGGTCTTGAGGGGTTTTTTGGGTACCCCCGG
TAAACCAGCAATAGACATAAGCGGCTATTTAACGACCCTGCCCTGAACCGACGACCGGGTCATC
GTGGCCGGATCTTGCGGCCCCTCGGCTTGAACGAATTGTTAGACACTAAAACAATTCATCCAGT
AAAATATAATATTTTATTTTCTCCCAATCAGGCTTGATCCCCAGTAAGTCAAAAAATAGCTCGA
CATACTGTTCTTCCCCGATATCCTCCCTGATCGACCGGACGCAGAAGGCAATGTCATACCACTT
GTCCGCCCTGCCGCTTCTCCCAAGATCAATAAAGCCACTTACTTTGCCATCTTTCACAAAGATG
TTGCTGTCTCCCAGGTCGCCGTGGGAAAAGACAAGTTCCTCTTCGGGCTTTTCCGTCTTTAAAA
AATCATACAGCTCGCGCGGATCTTTAAATGGAGTGTCTTCTTCCCAGTTTTCGCAATCCACATC
GGCCAGATCGTTATTCAGTAAGTAATCCAATTCGGCTAAGCGGCTGTCTAAGCTATTCGTATAG
GGACAATCCGATATGTCGATGGAGTGAAAGAGCCTGATGCACTCCGCATACAGCTCGATAATCT
TTTCAGGGCTTTGTTCATCTTCATACTCTTCCGAGCAAAGGACGCCATCGGCCTCACTCATGAG
CAGATTGCTCCAGCCATCATGCCGTTCAAAGTGCAGGACCTTTGGAACAGGCAGCTTTCCTTCC
AGCCATAGCATCATGTCCTTTTCCCGTTCCACATCATAGGTGGTCCCTTTATACCGGCTGTCCG
TCATTTTTAAATATAGGTTTTCATTTTCTCCCACCAGCTTATATACCTTAGCAGGAGACATTCC
TTCCGTATCTTTTACGCAGCGGTATTTTTCGATCAGTTTTTTCAATTCCGGTGATATTCTCATT
TTAGCCATACTCTTCCTTTTTCAATATTATTGAAGCATTTATCAGGGTTATTGTCTCATGAGCG
GATACATATTTGAATGTATTTAGAAAAATAAACAAATAGCTAGCTCACTCGGTCGCTACGCTCC
GGGCGTGAGACTGCGGCGGGCGCTGCGGACACATACAAAGTTACCCACAGATTCCGTGGATAAG
CAGGGGACTAACATGTGAGGCAAAACAGCAGGGCCGCGCCGGTGGCGTTTTTCCATAGGCTCCG
CCCTCCTGCCAGAGTTCACATAAACAGACGCTTTTCCGGTGCATCTGTGGGAGCCGTGAGGCTC
AACCATGAATCTGACAGTACGGGCGAAACCCGACAGGACTTAAAGATCCCCACCGTTTCCGGCG
GGTCGCTCCCTCTTGCGCTCTCCTGTTCCGACCCTGCCGTTTACCGGATACCTGTTCCGCCTTT
CTCCCTTACGGGAAGTGTGGCGCTTTCTCATAGCTCACACACTGGTATCTCGGCTCGGTGTAGG
TCGTTCGCTCCAAGCTGGGCTGTAAGCAAGAACTCCCCGTTCAGCCCGACTGCTGCGCCTTATC
CGGTAACTGTTCACTTGAGTCCAACCCGGAAAAGCACGGTAAAACGCCACTGGCAGCAGCCATT
GGTAACTGGGAGTTCGCAGAGGATTTGTTTAGCTAAACACGCGGTTGCTCTTGAAGTGTGCGCC
AAAGTCCGGCTACACTGGAAGGACAGATTTGGTTGCTGTGCTCTGCGAAAGCCAGTTACCACGG
TTAAGCAGTTCCCCAACTGACTTAACCTTCGATCAAACCACCTCCCCAGGTGGTTTTTTCGTTT

ACAGGGCAAAAGATTACGCGCAGAAAAAAAGGATCTCAAGAAGATCCTTTGATCTTTTCTACTG
AACCGCTCTAGATTTCAGTGCAATTTATCTCTTCAAATGTAGCACCTGAAGTCAGCCCCATACG
ATATAAGTTGTAATTCTCATGTTAGTCATGCCCCGCGCCCACCGGAAGGAGCTGACTGGGTTGA
AGGCTCTCAAGGGCATCGGTCGAGATCCCGGTGCCTAATGAGTGAGCTAACTTACATTAATTGC
GTTGCGCCCGCTCTAGATAATACGACTCACTATAGGAAAAAAACTTTATATAACTCTTATATAA
GTGCCCTTAGCGGGGCTCCCCGGCTACGGTCGGATCCCTACGGGGAGGATAGGGTGAAAACCCC
TAGTGCAAGCTGACACTCATACCTCCCAAGGTCCATGAGTCGACGCAAAGGTCCTCGAAAGCAT
GTTGTCCTTTCGTACAACCGAGTAGGTTCGTTGCCTTAATTGGTGACGCTTGCAGGATGAGGAT
GGTCCCGACGCCTAACGGACCTTGCTGCCTTCTTTCCCTGGATTGGCGGTGTTGTTCCCACTAA
TAATAAAGGAATACGCACATGTTGCTGCCTGTAGTAGCCCGTGCGGCCGTCCCTGCTATTGAGA
GTGCCATTGCGGCTACTCCTGGCCTGGTTTCCCGAATCGCAGCCGCGATCGGTTCCAAGGTCAG
CCCTTCCGCCATTTTGGCGGCGGTCAAGAGCAACCCGGTCGTCGCAGGTCTGACACTCGCTCAG
ATCGGAAGCACCGGTTATGACGCCTATCAGCAGCTTCTGGAGAATCATCCAGAGGTCGCCGAGA
TGCTGAAAGACCTGTCTTTCAAAGCCGACGAAATCCAGCCGGATTTCATCGGTAACCTCGGTCA
GTACCGCGAAGAGCTGGAACTGGTCGAAGATGCTGCCCGCTTCGTGGGCGGCATGTCGAACCTG
ATTCGCCTGCGCCAGGCCCTGGAGCTTGATATCAAGTACTACGGCCTGAAAATGCAGCTGAATG
ACATGGGATACCGCTCGTAATGGTTATCGGTCTTCTGAAGTATCTCACGCCTGCCGTTAAGGTG
CAGATGGCTGCTCGCGCGTTGGGCCTGTCCCCCGCCGAAGTCGCTGCAATTGACGGCACGTTGG
GTCGTGTCTCTGCGATGCCAGCGGTCGCGGTCGTGCTGGGAGGGAAACCTCTCTCTCTGGCCAC
GATCGCGTCAGTTGTGTCTGATGCAAACCCCAGTGCCACTGTTGGCGCGCTTATGCCTGCTGTA
CAGGGCATGGTGAGTTCCGACGAAGGCGCGAGTGCGTTGGCTAAGACCGTGGTAGGCTTCATGG
AGTCCGACCCCAACAGCGATGTCCTGGTTCAACTGCTCCACAAGGTGTCAAACTTGCCGATTGT
CGGCTTTGGTGACACGCAGTATGCAGACCCAGCTGACTTCTTGGCCAAGGGAGTTTTCCCTCTG
ATCAGGAAGCCAGAAGTAGAGGTTCAAGCTGCGCCTTTCACCTGTCGTCAGTGTGATCATGTTG
ATCACATCACTGATGTACCTCAAACTTCGACCTTTGTTCACAAATGCACTTCGTGCGGCTTTGT
GCAGATGGTCCACCGTAAGGATGTTCCGTAATGCCATTTCCTCTGGTAAAGCAAGACCCAACCT
CGAAGGCTTTCACTGAAGCCAGTGAACGCTCCACCGGCACCCAGATCCTGGACGTCGTCAAGGC
CCCTATCGGCCTGTTCGGCGACGATGCCAAACACGAGTTCGTGACCCGTCAGGAACAAGCCGTT
TCCGTCGTCAGCTGGGCAGTTGCTGCCGGTCTGATCGGCGAGCTGATCGGCTACCGTGGTGCGC
GTTCGGGTCGCAAAGCGATCCTGGCCAACATCCCTTTTCTGGCCTAACTCCTCGTGTCCAAGGA
TAGCGCCTTCGCAGTGCAATACTCGCTGCGCGCCCTGGGACAAAAGGTGCGGGCAGACGGGGTA
GTGGGCTCTGAGACTCGTGCCGCGCTGGATGCGCTGCCCGAGAATCAGAAGAAAGCGATTGTAG
AGTTGCAAGCACTCCTACCGAAAGCACAGTCGGTCGGCAACAACCGTGTGAGGTTCACAACAGC
TGAAGTCGACTCGGCGGTGGCGCGGATCTCGCAAAAGATAGGTGTTCCGGCTTCCTACTACCAG
TTCCTGATTCCGATCGAGAACTTCGTGGTGGCCGGTGGTTTCGAAACCACCGTTTCTGGTTCCT
TCCGTGGGTTGGGCCAGTTCAACCGGCAGACGTGGGATAGACTCCGTCGTTTAGGCCGTAACCT
TCCTGCATTTGAGGAGGGTTCGGCACAACTGAACGCTTCTCTTTATGCAATCGGGTTCTTGTAT
CTTGAGAACAAGAGAGCGTACGAGGCGTCGTTCAAAGGCCGCGTTTTCACTCACGAAATCGCGT
ATTTGTATCACAACCAAGGCGCTCCAGCTGCCGAACAGTACCTGACTTCGGGTCGGCTCGTTTA
CCCGAAGCAAAGCGAGGCCGCTGTCGCGGCGGTTGCGGCTGCGAGAAACCAGCATGTCAAAGAG
AGTTGGGCTTAGCCCTGAACTGCATCGTGAACTGAAAATGTTCCCAGATGTCACGAAGGGTGGC
ACGTTCGACATAACCATCCGGTCGACTACCGAGAACGGTGCTTTTTGGGCGAACTACGAAGGTA
GAACGTCCTTGGTCACCGTCCCGGACGTGAAGACAGCTATCGAGTTTTTGATTAAACTCTGCCG
TCGACACAAGTTGTCCAATCAGGTGAACACGCGAACGCTTCTCCGCGATTTGCAACGAACGTTG
CAGGAATGTGAATGCCAGTCTCATCATGTGCCGTTGTCCAGCCCCTTCATGCATCTCAGATTTG
CGTAAAGCTGATCGGAAGCTATGAAAGTAAGCTGAGCGACACGGAAGTTATTGAAGCAGCTATC
AAGCTCTCATAGGCTTGGAAAGCCCGGCATCGATGTCGTTCCATGTCGCGCCAGACGCGGCCAC

CGATATGTATCTTGATCTGATCGAAATCTACTCCCCGTCGTCAGTCGGGATACATCTCGTCCTG
CCATAAGCGCTGTCTGTAGCGTGCATAAACAGATAGATCGCCTTTTTAGGTAACCGCGGATTGA
TCACCGTTCCGAGCTTGCTTGGATAAACAAGTCCTTGTATAACAAGGCGAGACTCACTATGTGA
GCGTCCAATAGGACGGCCCCTTCGGGGGCTCTCTCTCT

**>pJLC142 (p15A ori, SpecR, contains phi6 M cDNA segment under T7 transcriptional control)**

TTTCCATAGGCTCCGCCCCCCTGACAAGCATCACGAAATCTGACGCTCAAATCAGTGGTGGCGA
AACCCGACAGGACTATAAAGATACCAGGCGTTTCCCCCTGGCGGCTCCCTCGTGCGCTCTCCTG
TTCCTGCCTTTCGGTTTACCGGTGTCATTCCGCTGTTATGGCCGCGTTTGTCTCATTCCACGCC
TGACACTCAGTTCCGGGTAGGCAGTTCGCTCCAAGCTGGACTGTATGCACGAACCCCCCGTTCA
GTCCGACCGCTGCGCCTTATCCGGTAACTATCGTCTTGAGTCCAACCCGGAAAGACATGCAAAA
GCACCACTGGCAGCAGCCACTGGTAATTGATTTAGAGGAGTTAGTCTTGAAGTCATGCGCCGGT
TAAGGCTAAACTGAAAGGACAAGTTTTGGTGACTGCGCTCCTCCAAGCCAGTTACCTCGGTTCA
AAGAGTTGGTAGCTCAGAGAACCTTCGAAAAACCGCCCTGCAAGGCGGTTTTTTCGTTTTCAGA
GCAAGAGATTACGCGCAGACCAAAACGATCTCAAGAAGATCATCTTATTAATCAGATAAAATAT
TACTAGATTTCAGTGCAATTTATCTCTTCAAATGTAGCACCTGAAGTCAGCCCCATACGATATA
AGTTGTTACTAGTGCTTGCTAGCATAACCCCTTGGGGCCTCTAAACGGGTCTTGAGGGGTTTTT
TGATTATTTGCCGACTACCTTGGTGATCTCGCCTTTCACGTAGTGGACAAATTCTTCCAACTGA
TCTGCGCGCGAGGCCAAGCGATCTTCTTCTTGTCCAAGATAAGCCTGTCTAGCTTCAAGTATGA
CGGGCTGATACTGGGCCGGCAGGCGCTCCATTGCCCAGTCGGCAGCGACATCCTTCGGCGCGAT
TTTGCCGGTTACTGCGCTGTACCAAATGCGGGACAACGTAAGCACTACATTTCGCTCATCGCCA
GCCCAGTCGGGCGGCGAGTTCCATAGCGTTAAGGTTTCATTTAGCGCCTCAAATAGATCCTGTT
CAGGAACCGGATCAAAGAGTTCCTCCGCCGCTGGACCTACCAAGGCAACGCTATGTTCTCTTGC
TTTTGTCAGCAAGATAGCCAGATCAATGTCGATCGTGGCTGGCTCGAAGATACCTGCAAGAATG
TCATTGCGCTGCCATTCTCCAAATTGCAGTTCGCGCTTAGCTGGATAACGCCACGGAATGATGT
CGTCGTGCACAACAATGGTGACTTCTACAGCGCGGAGAATCTCGCTCTCTCCAGGGGAAGCCGA
AGTTTCCAAAAGGTCGTTGATCAAAGCTCGCCGCGTTGTTTCATCAAGCCTTACGGTCACCGTA
ACCAGCAAATCAATATCACTGTGTGGCTTCAGGCCGCCATCCACTGCGGAGCCGTACAAATGTA
CGGCCAGCAACGTCGGTTCGAGATGGCGCTCGATGACGCCAACTACCTCTGATAGTTGAGTCGA
TACTTCGGCGATCACCGCTTCCCTCATACTCTTCCTTTTTCAATATTATTGAAGCATTTATCAG
GGTTATTGTCTCATGAGCGGATACATATTTGAATGTATTTAGAAAAATAAACAAACTCAGTAAT
ACGACTCACTATAGGAAAAAAACTTTATATATTTTCTACGTTGAGCTCCGTATAAAGCTCCGTG
CCCGCACACGCCCGCTACGGCGGTATTGTCTAACCGGCGACAATAAACAGCTGCTGCTTACAAG
CTTACAGTTGACCGGAGTCTCGGCGTGCAGCGCCTAAACACGGGAAACCGTGGTGGTGACACCC
TCTGCTGAGGGCTTATAGTGGTGATATTCCTCCCCAGGAGTTCCCTCCCATTTCGGCCACTGCG
CTCTAACCATGAGCGCGGTCTATTTGAGAGTGTCGCTCTTCTGCCTACGCGCTCATTCGTTCCC
TCGAGTTGACGCTTCAAGCAGGTGGACACCTCCTCAACCCATAATAAGAGATCCATTCAATGGA
CAACATCCTCGATCCCCTTAAGGCTCCGTTTTCTTCGGAAGCCGCCGCGAAAACCACCGCTGCC
AAAATCGCTGTGGTATACGCGTTGGTCGGTCTGGTTGGCGGTCTGCTGCTCACCAAGTAAGGTG
TAGTATGCATGACACGCGACCGCTCCGAACCGAAAGACCCATGGCCAGCAAGAATACGAATGAC
CGGGTTTTCGACCGGTTTCATTCTTCTCTCGTTCGGCTGTGGCATTCTGCAAACCAGCGCATGC
GCGGTTCTTTCTCCGTCGTGGTTCGGGTCGAGCACTCTTTAGTGCTCCTCATCGGCTACACGGT
GGTAGGCGCGACTGTCGCACACTTCGTGAGGTGACTATGTTAGCTTTCGTAGCGCGAGCGGTCG
TACTTTACTCTGCTGGTGTAGTCGTGGGCATCGCCTACGATCACGTCACAGGAAGGAAACGTCG
CCATGACTAAGTGGAAGATGTACATCGCCGGCGTCGTTCTGGTCATCGTAGGGGCAGTTACTCA

TGCTCCACAGCTGATGGTCCAGGGCATGACTACGCTCGCGACTCAAGCGGCCGCAGACGCGGCC
GATGGTGGAGGTGCTCAGTGAGTATCTTCTCCTCGTTGTTCAAGGTCATCAAGAAGGTAATCTC
GAAGGTGGTCGCCACCCTTAAGAAAATCTTCAAGAAGATCTGGCCGTTGCTACTTATTGTGGCA
ATTATCTACTTCGCTCCCTACCTCGCCGGGTTCTTCACTTCCGCCGGGTTCACTGGGATCGGAG
GGATCTTCTCCTCTATCGCAACCACCATCACGCCTACGCTGACGTCGTTCCTGTCGACTGCGTG
GTCTGGTGTGGGCTCTCTTGCCTCCACGGCTTGGTCTGGGTTCCAATCTCTCGGGATGGGTACT
CAGCTCGCTGTCGTGAGTGGCGCGGCTGCTCTGATTGCACCTGAGGAAACGGCTCAACTGGTTA
CCGAAATCGGTACCACCGTAGGTGATATCGCCGGTACGATTATCGGCGGTGTCGCCAAGGCACT
CCCGGGTTGGATCTGGATCGCCGCAGGCGGTCTTGCCGTCTGGGCCCTCTGGCCGTCATCTGAC
AGTAAGGAGTAGCAAATGCGCTACCAAGGCATCAACGAGTGGCTGGGTGGAGCCAAGAAACTCA
CCACCGCAAACGGTGAGATTGGCGCTATCTACCTCTCCGCTGCTCCTCCCACCGACGCCGCACG
TGCGGACGCTAAGGCGGTGGATTTTACTGCTGGTTGGCCAAGCGCGATCGTTGACTGCGCTGAT
GCCACTCGTGCCAAGCAGAACTACCTGTGGGTTGGCGATAACGTTGTGCACATCGGGGCTAAAC
ACGTTCCACTCCTCGATCTGTGGGGCGGGACAGGTGATGCCTGGCAGCAGTTCGTTGGCTATGC
CTGCCCAATGCTCGACCTTTGTCGTGCGTGGGGCCTGGGTTATGCCAGCGCTTCTGTAACCACC
GGCTCGTTGCAGGGCTATCAGCCATCGGCGTTCTTGGACGTTGAGCAACAGCAGTTCGCGAAGG
ACAATCTCAACCTGTATGGCGATAACTGCCTTGACCTGGCCACCAGTTCGTCCGCTCAGCGGGC
ATTTCTGGAGCAGTGCATGGGCTGCGCCTTGCCGGAGGATTGCATCTTCGGTTGGTATGTGAAA
ATGGATTGGGAAGGTTCGGCAGTTGCCGACGCCTACGCTGCGATCCGTGTCCAAGGGTTCGCCA
CTGTAATGGCACCTTGGCAGTCGGTTGGCGGTGCTGGCTACGTTTACGCTCGTGTGCCTCAAAA
AGGCGCGTGGATGGGTGTGAACCTGCTTGCCTATGTCCACGGCACCAGTGGCCAGCCTGCTTAT
GGCATTCCGATGACCCTCTCGGGGTTCACCGGTAACATGGGTCAGGTGGCTTCGAAGTGGCTCA
TGCTTCCTCTCCTGATGATCGTCGACCCTCATGTCGTCCAGATTTTGGCCGCACTGGGGGTTAA
ACGTGGGACCAAATCGGACCCACGGACGACCGACGTGTACGCTGATCCGAAGGTTCCGGCTAGC
CGTATTTCCGGGCCGATGATCAATGGAACGGTTGCTCCTCCTGCGACGATCCCCGCTACCATTC
CGGTGCCTCTGGCGCCGCTCGGTGGCGCGGGTGGCCCTGGCGCTCAGGGTTTCCAGGTATACCC
CGTTTTCACCTGGGGTCTGCCTGAGTTCATGACCGACGTGACCATCGAAGGTACCGTCACTGCG
GACTCCAACGGTCTGCATGTCGTGGACGACGTGCGTAACTACGTCTGGAACGGTACTGCTCTTG
CTGCAATTGAGCAGGTCAATGCCGCTGACGGTCGAGTTACGCTCACTGACTCTGAGCGTGCTCA
ACTCGCCTCGTTGACTGTTCGAACCGCATCGTTGCGTCAGCAGCTGTCGGTTGGGGCAGACCCC
TTGTCCAAGACGTCGATCTGGCGTCGGGCTCAAAAGGCCGATTATGATCTGCTGTCTCAACAGA
TCATCGAAGCGGACACGGTGAAAAACCTACCTGCTGTGACGTTCGCTCAGGCGAACAAAGCGGC
AGGCGGTCAATCCGAGACATTGTGGCACCAGATGTATCGGGTCAACGATATCGCTGGCGATCAA
GTCACCGCAATCCAAATCACTGGTACGATGGCGACTGGCATTCGATGGTCGGCAACTGCTGGCG
GTCTGGTCGTCGATGCTGACGAGCAAGATGCGGTGATCGCGATTTCGTCCGGTAAGCCGGTCAA
GAACAGCTCCGACCTTCCTACGGCCGACGCTGTGAACTACTTGTTCGGTATCACTGCGGACGAT
ATGCCTGGTATCGTTTCCTCGCAAAAGGAAATGAACAGCGAGTTTGAAGAAGGTTTCCTTCAGA
AAGCTCGTCTTTGGAACCCACGTAAGCTCGTCGAAAACGTCCAGAATGCCTATTTCCTGATGGT
GTACGCTCGCGATCGGAAGCAATTCCACTCGTTGGTGGCATCCTCTCTGGCGATGGCCAAGCTG
GGCGTAAGTACGCGGGCCTGTAAGGAGTCGTATGGCTGCTGAACAATCCTCCGGTATGAGCGCG
TTCACCAAAGGCACGATCGTGATCTGCCTGGTGGTGGTCGTCCTCAATCTCATCGGGAAGTGAC
CATGGTACCGCTAAAAATTAGCACGCTGGAGTCCCAGCTGCAACCGCTTGTTAAGTTGGTTGCA
ACCGAAACCCCCGGTGCCCTCGTAGCGTATGCTCGAGGGTTATCGAGTGCCGACCGCTCGCGGT
TGTACAGACTGCTTCGTTCTTTGGAGCAGGCCATCCCGAAGCTGTCGTCGGCTGTCGTTTCGGC
CACGACGTTGGCAGCGCGAGGTCTTTAATGGAAACCAACCCGCTGCTTCAGCTTGAGTCGCTGT
CGTTACGCTTGCGAGACATGCCTCGTTCGCGCCTTTCTGCGCTGATGAAGAACATGTCGTATGA
GCAGCTGCAGTCGTTGTATAGCACCAGCGTAAAAGTTGGCGCTGTGCTCGATAGCGTTTCAATG

CAGTTGCTTGAGGCGTCACAAACCGCTCAATCGGGAACTCGACTGATGACACCGCAGGAGTACG
TCGCTGCTGGTGGAGGTCGTGTGTACGTTAAATAAGTCCTTAGATTTCTAAGGCGAGACTCGCT
TTGCGAGCATCCAATAGGATGGCCCCTTCGGGGGCTCTCTCTCTCTAGCATAACCCCTTGGGGC
CTCTAAACGGGTCTTGAGGGGTTTTTTGGGATCCATTCACCACCCTGAATTGACTCCCTTAACG
CTTCATCCGCCGCCCTAGACCTAGGGATATATTCCGCTTCCTCGCTCACTGACTCGCTACGCTC
GGTCGTTCGACTGCGGCGAGCGGAAATGGCTTACGAACGGGGCGGAGATTTCCTGGAAGATGCC
AGGAAGATACTTAACAGGGAAGTGAGAGGGCCGCGGCAAAGCCGTT

**>pJLC143 (pSC101 ori, CmR, contains phi6 L cDNA segment under T7 transcriptional control)**

TCTCTCTAGCATAACCCCTTGGGGCCTCTAAACGGGTCTTGAGGGGTTTTTTGCATGGTCATAG
CTGTTTCCTGTGTGAAATTGTTATCCGCTCACAATTCCACACAACATACGAGCCGGAAGCATAA
AGTGTAAAGCCTGGGGTGCCTAATGAGTGAGCTAACTCACATTAAAATATAATGACCCTCTTGA
TAACCCAAGAGGGCATTTTTTAATGCCCATGGCGTTTACGCCCCGCCCTGCCACTCATCGCAGT
ACTGTTGTAATTCATTAAGCATTCTGCCGACATGGAAGCCATCACAGACGGCATGATGAACCTG
AATCGCCAGCGGCATCAGCACCTTGTCGCCTTGCGTATAATATTTGCCCATGGTGAAAACGGGG
GCGAAGAAGTTGTCCATATTGGCCACGTTTAAATCAAAACTGGTGAAACTCACCCAGGGATTGG
CTGAGACGAAAAACATATTCTCAATAAACCCTTTAGGGAAATAGGCCAGGTTTTCACCGTAACA
CGCCACATCTTGCGAATATATGTGTAGAAACTGCCGGAAATCGTCGTGGTATTCACTCCAGAGC
GATGAAAACGTTTCAGTTTGCTCATGGAAAACGGTGTAACAAGGGTGAACACTATCCCATATCA
CCAGCTCACCGTCTTTCATTGCCATACGGAATTCCGGATGAGCATTCATCAGGCGGGCAAGAAT
GTGAATAAAGGCCGGATAAAACTTGTGCTTATTTTTCTTTACGGTCTTTAAAAAGGCCGTAATA
TCCAGCTGAACGGTCTGGTTATAGGTACATTGAGCAACTGACTGAAATGCCTCAAAATGTTCTT
TACGATGCCATTGGGATATATCAACGGTGGTATATCCAGTGATTTTTTTCTCCATTTTAGCTTC
CTTAGCTCCTGAAAATCTCGATAACTCAAAAAATACGCCCGGTAGTGATCTTATTTCATTATGG
TGAAAGTTGGAACCTCTTACGTGCCGATCAAGGCCAAATAGGCCGTTCAGATCCTTCCGTATTT
AGCCAGTATGTTCTCTAGTGTGGTTCGTTGTTTTTGCGTGAGCCATGAGAACGAACCATTGAGA
TCATGCTTACTTTGCATGTCACTCAAAAATTTTGCCTCAAAACTGGTGAGCTGAATTTTTGCAG
TTAAAGCATCGTGTAGTGTTTTTCTTAGTCCGTTACGTAGGTAGGAATCTGATGTAATGGTTGT
TGGTATTTTGTCACCATTCATTTTTATCTGGTTGTTCTCAAGTTCGGTTACGAGATCCATTTGT
CTATCTAGTTCAACTTGGAAAATCAACGTATCAGTCGGGCGGCCTCGCTTATCAACCACCAATT
TCATATTGCTGTAAGTGTTTAAATCTTTACTTATTGGTTTCAAAACCCATTGGTTAAGCCTTTT
AAACTCATGGTAGTTATTTTCAAGCATTAACATGAACTTAAATTCATCAAGGCTAATCTCTATA
TTTGCCTTGTGAGTTTTCTTTTGTGTTAGTTCTTTTAATAACCACTCATAAATCCTCATAGAGT
ATTTGTTTTCAAAAGACTTAACATGTTCCAGATTATATTTTATGAATTTTTTTAACTGGAAAAG
ATAAGGCAATATCTCTTCACTAAAAACTAATTCTAATTTTTCGCTTGAGAACTTGGCATAGTTT
GTCCACTGGAAAATCTCAAAGCCTTTAACCAAAGGATTCCTGATTTCCACAGTTCTCGTCATCA
GCTCTCTGGTTGCTTTAGCTAATACACCATAAGCATTTTCCCTACTGATGTTCATCATCTGAGC
GTATTGGTTATAAGTGAACGATACCGTCCGTTCTTTCCTTGTAGGGTTTTCAATCGTGGGGTTG
AGTAGTGCCACACAGCATAAAATTAGCTTGGTTTCATGCTCCGTTAAGTCATAGCGACTAATCG
CTAGTTCATTTGCTTTGAAAACAACTAATTCAGACATACATCTCAATTGGTCTAGGTGATTTTA
ATCACTATACCAATTGAGATGGGCTAGTCAATGATAATTACTAGTCCTTTTCCTTTGAGTTGTG
GGTATCTGTAAATTCTGCTAGACCTTTGCTGGAAAACTTGTAAATTCTGCTAGACCCTCTGTAA
ATTCCGCTAGACCTTTGTGTGTTTTTTTTGTTTATATTCAAGTGGTTATAATTTATAGAATAAA
GAAAGAATAAAAAAAGATAAAAAGAATAGATCCCAGCCCTGTGTATAACTCACTACTTTAGTCA
GTTCCGCAGTATTACAAAAGGATGTCGCAAACGCTGTTTGCTCCTCTACAAAACAGACCTTAAA

ACCCTAAAGGCTTAAGTAGCACCCTCGCAAGCTCGGGCAAATCGCTGAATATTCCTTTTGTCTC
CGACCATCAGGCACCTGAGTCGCTGTCTTTTTCGTGACATTCAGTTCGCTGCGCTCACGGCTCT
GGCAGTGAATGGGGGTAAATGGCACTACAGGCGCCTTTTATGGATTCATGCAAGGAAACTACCC
ATAATACAAGAAAAGCCCGTCACGGGCTTCTCAGGGCGTTTTATGGCGGGTCTGCTATGTGGTG
CTATCTGACTTTTTGCTGTTCAGCAGTTCCTGCCCTCTGATTTTCCAGTCTGACCACTTCGGAT
TATCCCGTGACAGGTCATTCAGACTGGCTAATGCACCCAGTAAGGCAGCGGTATCATCAACAGG
CTTACCCGTCTTACTGTCAAGAGGACATCCGGTAATACGACTCACTATAGTAAAAAAACTTTAT
ATAGTCTTTTACCTGGATTCTCTGTGCAGAACTGAGAACTGAACGCTACCCTTGCGGGGGATGC
GGCCCCGGGCTACGGCCTAGGGATCCAGCGTGGCTCACGGGCCGCCGGAACTGACGTCCGTAAC
AAACGTCCTTGGGATAGGAGTACAGTAACCACTCTTAGATACCCGATTCCCCTGTTTCTGCGTG
GAAGCCTTTCGACAGCTACCCAGCTTAGATCGTCTGGTGCCCTAAATCCCTGGAGATAACCAAT
GGCTACATTACAAGATGTGCATCTACGGGTGAATGACCGGGTAACACCGGTGTACTTCACTGCT
CGCTCGTTTCTGCTCGTTTCTCCGAAACGTGCGGGGCAAGCAACGTTCCTCGCTCGCGAGGAGG
GTACTGACAATCCTGTCGTTACCTGTCATGTATCCGACTTTTATAAGGACGGTGTGTAATGACT
TTGTACCTGGTCCCTCCGCTGGATTCGGCGGACAAAGAGTTGCCTGCTCTGGCTTCCAAAGCTG
GGGTAACGCTTCTCGAGATCGAGTTTCTTCACGAGCTCTGGCCTCACCTCAGTGGTGGTCAGAT
CGTGATCGCCGCTCTCAACGCCAACAATCTGGCCATCCTCAACCGTCACATGTCCACTCTGTTG
GTCGAGTTGCCGGTTGCTGTGATGGCCGTTCCCGGTGCTAGCTATCGTTCCGATTGGAACATGA
TCGCTCACGCACTCCCGTCTGAGGATTGGATCACTTTGTCCAACAAGATGCTGAAAAGCGGCTT
GCTGGCGAACGATACCGTCCAGGGCGAGAAGCGCTCCGGCGCTGAGCCGCTGTCGCCGAACGTG
TACACCGATGCGCTCTCGCGTCTCGGTATCGCGACGGCCCATGCTATCCCCGTTGAACCCGAAC
AACCGTTCGATGTCGATGAGGTAAGCGCCTGATGCCGAGGAGAGCTCCCGCGTTCCCTCTGAGC
GATATCAAGGCTCAGATGCTGTTCGCAAATAACATCAAGGCCCAACAAGCCTCGAAGCGTAGCT
TCAAAGAGGGGGCGATTGAAACGTACGAAGGGCTGCTTTCAGTAGACCCTCGGTTTTTGAGTTT
CAAGAACGAGCTCTCTCGGTATCTGACCGACCACTTCCCGGCGAACGTCGACGAGTATGGTCGT
GTTTATGGAAACGGTGTTCGTACCAACTTCTTTGGTATGCGCCACATGAACGGGTTTCCAATGA
TCCCCGCGACGTGGCCACTCGCTTCCAACCTTAAGAAACGTGCCGACGCTGACCTAGCCGATGG
CCCTGTTTCTGAGCGCGACAATCTACTCTTTCGCGCCGCAGTCCGGCTTATGTTTTCAGATCTA
GAGCCTGTTCCGCTGAAGATCCGTAAAGGATCGTCAACCTGCATCCCGTATTTTTCTAACGATA
TGGGAACGAAGATCGAGATCGCCGAGCGCGCTCTTGAGAAAGCGGAAGAAGCTGGCAATCTGAT
GCTGCAAGGTAAGTTTGATGACGCCTACCAGCTCCACCAAATGGGTGGTGCCTATTACGTCGTG
TATCGTGCACAATCGACCGATGCTATCACACTCGACCCTAAGACCGGAAAATTCGTGTCAAAGG
ATCGTATGGTCGCTGACTTCGAATACGCAGTCACGGGCGGTGAGCAAGGCTCGCTGTTCGCTGC
TTCGAAGGATGCCTCTCGTTTGAAGGAACAGTACGGGATAGATGTCCCGGACGGGTTTTTCTGC
GAGCGGCGTCGTACCGCTATGGGTGGTCCGTTCGCGTTGAACGCTCCTATCATGGCCGTTGCGC
AACCTGTGCGAAACAAAATTTACTCCAAGTACGCTTACACCTTTCACCATACTACTCGTCTTAA
TAAGGAGGAAAAGGTGAAAGAGTGGTCGTTGTGCGTCGCTACTGACGTATCCGACCACGACACG
TTCTGGCCTGGATGGCTGCGGGATCTCATCTGTGATGAACTGCTCAACATGGGGTACGCTCCGT
GGTGGGTTAAGTTGTTCGAAACCTCGCTCAAACTGCCCGTTTACGTGGGCGCTCCTGCTCCTGA
GCAGGGCCACACGTTGTTGGGTGATCCGTCCAACCCTGATCTCGAAGTTGGGCTCTCGTCCGGA
CAAGGGGCGACCGACCTCATGGGCACGTTGCTCATGAGTATCACCTACCTGGTGATGCAACTTG
ATCACACCGCTCCTCACCTCAACAGTCGAATCAAGGACATGCCATCAGCATGCCGCTTTCTTGA
CTCGTATTGGCAAGGACACGAGGAGATCCGTCAGATCTCAAAATCTGATGATGCTATACTTGGC
TGGACCAAAGGTCGTGCTTTGGTTGGTGGTCATCGTTTGTTCGAGATGCTGAAAGAGGGTAAGG
TTAACCCCTCACCTTACATGAAGATCTCCTACGAGCACGGTGGCGCCTTCCTTGGTGACATCCT
GCTTTACGACTCGCGTCGTGAGCCTGGCTCTGCCATCTTCGTTGGTAACATCAACTCAATGCTG
AACAACCAGTTCAGCCCTGAGTACGGTGTCCAATCGGGCGTTCGCGACCGATCTAAGCGCAAAC

GGCCGTTCCCCGGTCTTGCTTGGGCGTCGATGAAAGATACCTACGGTGCCTGTCCGATCTACTC
TGATGTGCTGGAGGCGATCGAGCGTTGCTGGTGGAACGCGTTCGGTGAGTCGTACCGTGCGTAT
CGTGAAGATATGCTTAAACGCGACACTCTCGAACTATCACGCTACGTTGCGTCGATGGCTCGTC
AAGCCGGGCTGGCTGAACTCACTCCCATTGATTTGGAGGTGCTTGCTGACCCGAACAAACTCCA
GTATAAGTGGACCGAGGCCGATGTCTCGGCGAATATCCACGAGGTACTGATGCATGGCGTATCG
GTCGAAAAGACTGAGCGCTTTCTCCGTTCTGTAATGCCGAGGTAATCATGCCGATTGTCGTAAC
TCAAGCGCATATTGATCGTGTCGGCATCGCCGCCGATCTGCTCGATGCGTCTCCTGTGTCGCTT
CAAGTTCTTGGTCGCCCTACCGCGATCAACACTGTCGTCATCAAGACGTACATCGCTGCTGTTA
TGGAGCTCGCCTCCAAGCAAGGTGGTTCGTTGGCCGGTGTGGATATTCGTCCTTCGGTTCTGCT
GAAAGACACCGCTATCTTCACCAAGCCGAAGGCGAAGTCCGCTGACGTCGAATCTGATGTCGAC
GTTCTGGACACGGGGATTTACTCCGTTCCTGGACTGGCTCGCAAGCCTGTCACCCACCGTTGGC
CATCAGAGGGTATCTACTCTGGTGTCACAGCTCTGATGGGCGCTACCGGTTCCGGTAAGTCGAT
CACGCTGAACGAAAAGCTCCGTCCAGACGTCCTGATTCGTTGGGGCGAGGTGGCTGAAGCTTAC
GATGAGCTGGATACCGCCGTCCACATCTCGACTCTGGATGAGATGTTGATTGTGTGTATTGGCC
TGGGTGCACTCGGGTTCAACGTCGCTGTTGACTCGGTTCGTCCTCTGCTGTTCCGTCTCAAAGG
CGCCGCCTCTGCGGGGGGTATTGTGGCTGTGTTCTACAGCCTGTTGACCGATATCTCGAACTTG
TTCACACAATACGATTGCTCTGTCGTCATGGTCGTTAACCCGATGGTTGACGCTGAGAAGATCG
AGTACGTGTTCGGTCAGGTCATGGCTTCGACTGTCGGTGCGATCTTGTGTGCTGATGGCAACGT
GTCCAGAACGATGTTCCGGACCAACAAAGGTCGTATTTTCAACGGTGCGGCCCCTCTTGCTGCT
GACACTCACATGCCTAGCATGGATCGTCCTACCAGCATGAAGGCCCTCGATCATACCTCGATCG
CCTCTGTCGCACCGCTGGAGCGTGGCTCCGTGGATACCGACGATCGCAATTCCGCTCCGCGCCG
TGGCGCTAACTTCTCTCTGTAAGGGTATAAGATGTTCAACCTCAAAGTTAAAGATCTGAACGGT
TCCGCTCGCGGTCTGACTCAAGCTTTCGCCATCGGCGAATTGAAGAACCAGCTGTCCGTCGGCG
CGTTGCAGTTGCCGTTGCAGTTCACGCGCACGTTCTCCGCTTCCATGACCAGCGAGTTGCTTTG
GGAAGTGGGCAAGGGCAACATCGACCCAGTGATGTACGCTCGTCTGTTTTTCCAGTACGCGCAA
GCTGGCGGCGCTCTGTCCGTTGATGAGCTCGTAAACCAGTTCACTGAGTATCACCAATCCACGG
CCTGTAACCCTGAAATCTGGCGCAAGCTGACTGCTTACATCACCGGTTCCTCGAACCGCGCGAT
CAAAGCTGACGCTGTAGGCAAGGTGCCTCCAACCGCGATCCTGGAGCAGTTGCGCACTCTCGCT
CCCTCGGAGCACGAGTTGTTTCACCACATCACGACCGACTTCGTCTGCCATGTGCTGTCTCCCC
TCGGTTTCATCCTGCCTGACGCTGCCTACGTGTACCGCGTTGGTCGCACCGCTACGTACCCCAA
TTTCTACGCTCTTGTAGATTGCGTACGTGCGAGCGACCTGCGTCGTATGCTGACAGCGCTGTCG
TCTGTCGATTCGAAGATGCTTCAAGCCACGTTCAAAGCCAAAGGCGCTCTTGCCCCTGCTTTGA
TCTCCCAGCATCTGGCTAACGCCGCCACTACTGCTTTCGAGCGGTCGCGCGGTAACTTCGATGC
CAATGCTGTGGTGTCGTCCGTTCTGACCATTCTTGGTCGTCTCTGGTCGCCTTCCACCCCGAAG
GAGCTCGACCCGAGTGCGCGTTTGCGCAACACCAACGGTATCGATCAGCTGCGCAGTAACCTGG
CGCTGTTCATCGCGTACCAGGATATGGTCAAGCAACGCGGTCGCGCCGAAGTCATCTTCTCTGA
CGAGGAGCTGTCGTCGACGATCATCCCTTGGTTCATCGAGGCGATGAGCGAAGTGTCCCCGTTC
AAACTGCGTCCGATCAACGAGACTACCAGCTATATCGGTCAGACCTCCGCGGTCGACCACATGG
GCCAGCCGAGCCATGTTGTGGTCTACGAAGACTGGCAGTTTGCCAAGGAGATCACCGCTTTCAC
TCCTGTCAAGCTGGCCAACAACTCGAATCAGCGTTTCCTGGACGTTGAGCCTGGTATCTCTGAT
CGTATGTCGGCTACGCTGGCACCAATCGGCAACACGTTCGCGGTTTCGGCGTTCGTCAAGAACC
GCACCGCCGTTTACGAGGCTGTTTCGCAGCGTGGTACAGTCAACAGCAACGGCGCGGAGATGAC
CCTCGGGTTCCCTTCCGTTGTTGAACGCGACTACGCTCTCGACCGTGACCCTATGGTCGCGATC
GCTGCTCTGCGCACTGGTATCGTCGATGAAAGTCTCGAGGCTCGCGCTTCGAACGATCTGAAAC
GGTCGATGTTCAACTACTACGCGGCTGTGATGCATTACGCTGTTGCTCACAATCCTGAAGTTGT
TGTTTCGGAGCACCAAGGTGTTGCCGCCGAACAAGGTTCGCTCTACCTGGTGTGGAACGTCCGC
ACTGAGCTGCGAATCCCTGTTGGTTACAACGCCATCGAGGGCGGTTCGATCCGTACCCCTGAGC

CGTTGGAGGCGATCGCCTACAACAAGCCGATCCAACCGTCCGAGGTGCTGCAAGCCAAGGTACT
GGATTTGGCTAACCACACAACCTCGATTCACATCTGGCCGTGGCATGAGGCTTCGACCGAGTTC
GCGTACGAAGACGCCTACTCTGTCACCATCCGCAACAAACGCTACACCGCCGAAGTCAAGGAGT
TCGAACTCCTCGGGCTCGGTCAACGTCGCGAACGTGTACGGATCCTCAAGCCTACGGTAGCCCA
CGCTATCATCCAGATGTGGTATTCCTGGTTCGTCGAGGACGACCGCACTTTGGCAGCTGCCCGT
CGCACGTCTCGCGATGACGCCGAGAAGCTTGCCATCGACGGTCGTCGTATGCAAAACGCTGTGA
CCTTGCTTCGCAAGATCGAGATGATTGGGACAACCGGTATCGGTGCGTCTGCCGTCCACCTCGC
GCAGTCGCGCATCGTGGATCAGATGGCCGGTCGAGGGCTCATCGACGACAGCTCCGATCTCCAT
GTCGGTATCAACCGTCACCGTATCCGCATCTGGGCCGGCCTCGCCGTTCTCCAGATGATGGGGC
TCTTGAGCCGCTCCGAAGCGGAAGCTCTCACCAAGGTCCTTGGTGATAGCAACGCTCTGGGCAT
GGTTGTCGCCACAACCGACATTGATCCATCCCTGTAACTCTCGTAAGCTCTCATAAACCTTTCG
TTATAATTCCATAAGTCCTTAGATTTCTAAGGCGAGACTCGCTTTGCGAGCGTCCAATAGGACG
GCCCCCTCGGGGGCTCTC
